# Prevalence and 20-year trends in meditation, yoga, guided imagery and progressive relaxation use among US adults from 2002 to 2022

**DOI:** 10.1038/s41598-024-64562-y

**Published:** 2024-07-01

**Authors:** Jonathan N. Davies, Anna Faschinger, Julieta Galante, Nicholas T. Van Dam

**Affiliations:** Contemplative Studies Centre, Melbourne School of Psychological Sciences, Faculty of Medicine, Dentistry, and Health Sciences, The University of Melbourne, Level 1, Melbourne Connect, 700 Swanston Street, Melbourne, VIC 3010 Australia

**Keywords:** Meditation, Yoga, Guided imagery, Progressive relaxation, Public health, NHIS, National health interview survey, Prevalence, Trends, CAM, Complementary and alternative medicine, Epidemiology, Risk factors, Psychiatric disorders

## Abstract

Meditation, yoga, guided imagery, and progressive relaxation are promoted as complementary approaches for health and wellbeing in the United States, but their uptake by different sociodemographic groups is unclear. This study assessed the prevalence and 20 year trends in the use of these practices in US adults between 2002–2022. We examined practice use and associations with sociodemographic and health factors in a population-weighted analysis of n = 134,959 participants across 5 cycles of the National Health Interview Survey. The overall use of meditation (18.3%, 60.53 million), yoga (16.8%, 55.78 million) and guided imagery/progressive relaxation (6.7%, 22.22 million) increased significantly from 2002 to 2022. Growth was consistent across most sociodemographic and health strata, however users of ‘Other’ race (comprising 54% Indigenous Americans, Odds Ratios; ORs = 1.28–1.70) and users with moderate (ORs = 1.19–1.29) psychological distress were overrepresented across all practices, and those with severe psychological distress were overrepresented in meditation (OR = 1.33) and guided imagery/progressive relaxation (OR = 1.42). Meditation use has accelerated over time for 65 + year olds (OR = 4.22), people not accessing mental health care (OR = 1.39), and less educated (OR = 4.02) groups, potentially reflecting unmet health needs. Health professionals should consider the extensive use of complementary practices in service and treatment planning and consider their risks and benefits.

## Introduction

The use of meditation, yoga, guided imagery, progressive relaxation and other complementary and alternative medical (CAM) practices by US adults has grown five-fold since the 1950s^1–8^. While CAM (also known as Integrative Medicine) practices include over 500 different approaches^9^, meditation, yoga, guided imagery, and progressive relaxation are among the most popular^4^, with potential benefits to mental health and wellbeing^10–12^ that are comparable in efficacy to psychotherapy and pharmacotherapy^13^. The practice of meditation, in particular, was introduced to the West through migration and cultural exchange where it was strategically adapted into secular formats to be empirically assessed, which led to greater acceptance within medical and mental health care systems^14^. These practices specifically, and other CAM approaches more generally, found broad appeal as tools for holistic health, illness prevention and health promotion^15^ and as a psychosocial adjunct for the treatment of serious illness and chronic disease^16,17^: appeals that have continued to grow alongside an increasing evidence base^18–20^ and media promotion^21^.

While meditation, yoga and guided imagery/progressive relaxation practices are broadly popular, a clear understanding of the demographic, cultural and socioeconomic determinants of their use is lacking, but likely to reflect a complex interplay between many factors. Studies internationally have consistently shown that CAM users are more likely to be female, White, middle-aged, wealthier, and better educated than non-users^22–24^ suggesting predominant use by privileged sociodemographic groups. Gender differences are reasonably well characterized, with being young or middle aged predicting CAM use in women, and older age, lower income, and being a widower associated with CAM use in men^25^. By contrast, the role of race/ethnicity in CAM use is more complex and nuanced^26^. CAM use is not consistently higher among White women than other groups when controlling for sociodemographic and health factors^26^. Other studies have found that Hispanic and Black people overall use CAM less than White people after controlling for other sociodemographic factors^26,27^, with Black CAM users generally older and less educated than White CAM users^28^. These findings suggest that racial/ethnic differences may be driven more by socioeconomic factors than cultural factors. A secondary analysis of National Health Interview Survey (NHIS) 2017 data found that the three strongest predictors of meditation use were: 1. the presence of a health problem, followed by 2. cost or access barriers to conventional medical care, then 3. individual characteristics^6^. Increases in CAM use among disadvantaged socioeconomic groups are purportedly driven by concerns about affordability of conventional medical care^27^, however these groups also cite increased care and work obligations as barriers to accessing CAM modalities^29^.

Traditional barriers to access have decreased markedly in recent years^30^ with the explosion of digital^31^ and app delivered meditation, yoga, and other digital mental health offerings. However, while current digital treatment offerings are largely low cost and broadly accessible^32,33^, how effectively they are enabling use by underrepresented sociodemographic groups in both research^34–36^ and practice^37^ is not yet clear. Meanwhile, recent data suggest significant unmet need for healthcare in the US, especially mental health services^38,39^. CAM practices have the potential to address some of the unmet need for mental health services because they ostensibly introduce new providers and offerings into the health system^38,40^, with digital offerings further reducing barriers associated with cost and access^32,33^. But no intervention is risk-free. Studies of meditation have highlighted potential harms that include prolonged adverse experiences among approximately 10% of users^41–43^. A recent cross-sectional survey found that low income and self-guided training via apps may be associated with adverse experiences, with first exposure via apps potentially associated with lasting impairment^42^ (noting that lower socioeconomic groups are also more likely to try digital mental health^44^, and also experience more acute distress and greater mental health need which itself predicts adverse experiences^43,45^). To plan appropriate public health services that maximize benefits and minimize harms, it is essential to know who is using CAM practices to understand whether they are safe, and suited to meet the needs for which they are being used.

The United States National Health Interview Survey (NHIS) has collected data on complementary practice use every 5 years since 2002. Analyses of NHIS CAM data until 2017 have reported regular increases in the use of meditation^4,5,46^, yoga^5,7,8^ and guided imagery/progressive relaxation^3,4,6^, with older, educated, White women the predominant users^47^. However, no publications have implemented population-weighted nationally-representative analyses of trends and patterns in the use of meditation^3–5,7^, yoga^8,48^, and guided imagery/progressive relaxation^3,4,6^ over a 20 year timespan using more than 3 timepoints.

The objective of this study was to report 2022 prevalence rates and 20 year trends from 2002–2022, and to determine whether and how subpopulation-specific prevalence of meditation, yoga and guided imagery/progressive relaxation use has changed over time. Such knowledge can help target culturally appropriate and accessible treatment and lifestyle assessments, health service planning, and research into the accessibility, effectiveness, and safety of these practices for different sociodemographic groups.

## Results

### Participant characteristics

Data from 134,959 participants (*M* = 49.4 years, SD = 18.4, 55.4% female) were used in weighted analyses across the 5 timepoints. Sample sizes ranged from 22,648 (2002) to 34,525 (2012). NHIS weighted data are nationally-representative of the civilian, non-institutionalized US adult population across sociodemographic characteristics including age, sex, race/ethnicity, socioeconomic status, region and urbanicity^49,50^. The ‘Other’ race/ethnicity category included a majority (54%) of Indigenous Americans (American Indian or Alaska Native, AIAN).

### Changes in prevalence of meditation, yoga, and guided imagery/progressive relaxation practices over 20 years (2002 to 2022)

**Figure 1:**
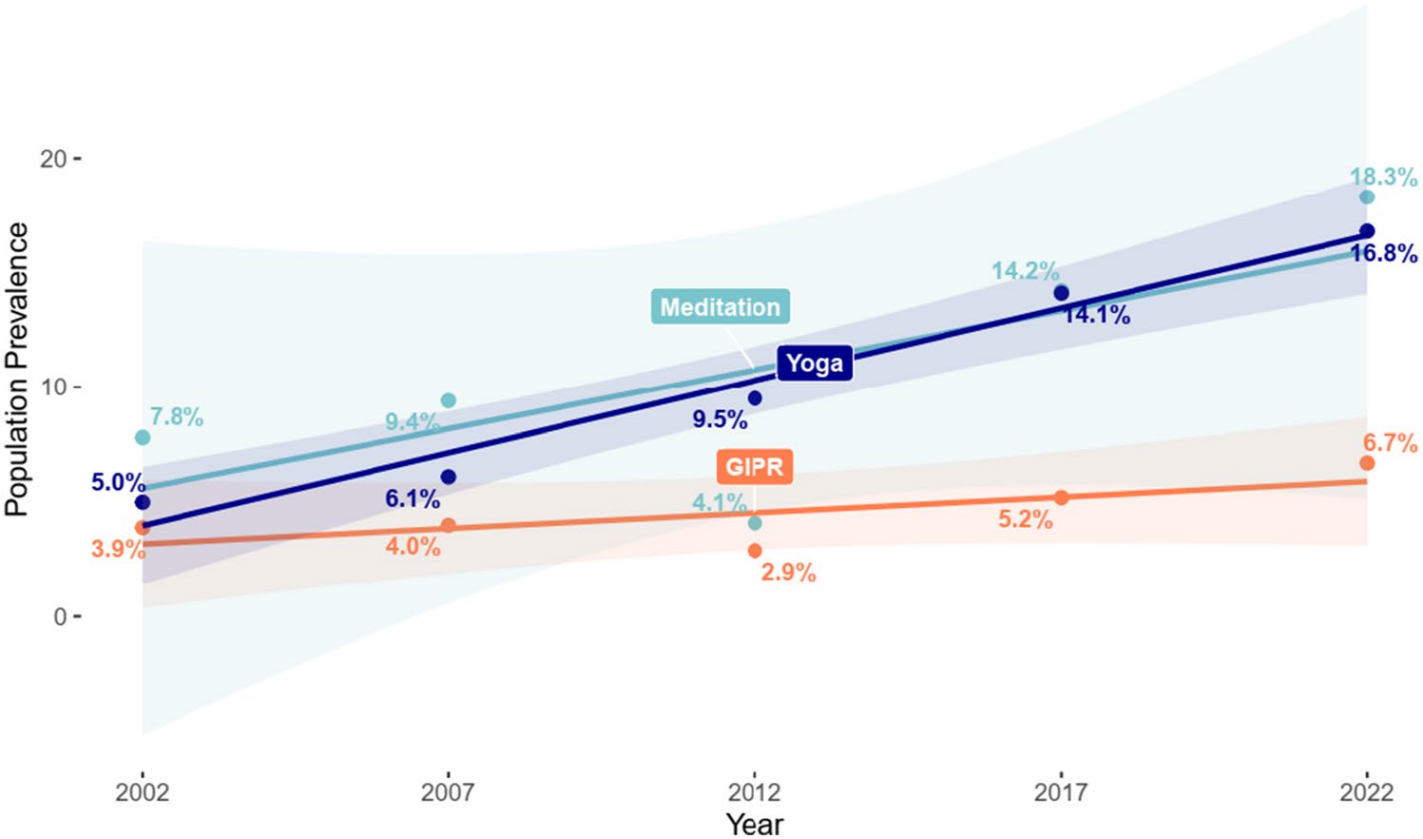
Prevalence and 20 year trends in meditation, yoga, and guided imagery/progressive relaxation between 2002–2022. Weighted population estimates (dots, percentage labels) and regressed growth rate (solid lines with error shading) of meditation, yoga, and guided imagery/progressive relaxation (GIPR) across 2002, 2007, 2012, 2017 and 2022. Error shading reflects 95% confidence intervals and is especially wide for meditation due to anomalous 2012 data. Source: NHIS Data 2002–2022.

In 2022, meditation was the most prevalent practice in the US adult population (population prevalence: 18.3%, 2020 population estimate^51^: N = 60.53 million), followed closely by yoga (16.8%, N = 55.78 million), with guided imagery/progressive relaxation used by substantially fewer people (6.7%, N = 22.22 million; Fig. 1). Across the 20-year period from 2002 to 2022, the population prevalence of all three practices increased, except for a dip in 2012, which was most prominent for meditation (Fig. 1, Figures S1–S3, Tables S1–S4). Yoga grew at the fastest rate (regressed annual growth: + 0.69%; Table S2), followed closely by meditation (+ 0.47%; Table S1). In addition to being the least prevalent practice group, guided imagery/progressive relaxation also grew at the slowest rate (+ 0.27%; Table S3).

### Meditation, yoga, and guided imagery/progressive relaxation use among sociodemographic and health subgroups in 2022

#### Meditation

The most highly represented sociodemographic subgroups among meditation users in 2022 were younger-to-middle-aged (25–34, 35–44), female, White or ‘Other’ race, not in a relationship, more educated (bachelor, master or higher), and residing in the West (Fig. 1, Table 1, Table S6). The least represented groups were young (18–24) or older (45–64, 65 +), male, Hispanic race, in a relationship, less educated (less than high school, high school) and residing in the South. Meditation use was not significantly different to the US population for Black and Asian users, and for those residing in the Northeast and Midwest.

**Table 1:** Sociodemographic and health composition of each user subpopulation in 2022 in comparison with the US Adult Population.

Characteristic	US adult population	Meditation users		Yoga users		Guided imagery/progressive relaxation users	
	%	%	Δ %	%	Δ %	%	Δ %
**Age, years**							
18–24	12.88	12.21 ***	− 0.67	13.50 ***	+ 0.62	10.21 ***	− 2.67
25–34	18.26	21.23 ***	+ 2.96	25.56 ***	+ 7.30	19.53 ***	+ 1.26
35–44	21.91	25.31 ***	+ 3.41	21.17 ***	− 0.74	19.45 ***	− 2.45
45–64	29.92	28.07 ***	− 1.85	28.47 ***	− 1.45	33.56 ***	+ 3.64
65 or above	17.03	13.18 ***	− 3.85	11.30 ***	− 5.73	17.24^ ns^	+ 0.21
**Gender**							
Male	51.36	39.30 ***	− 12.05	29.81 ***	− 21.55	33.93 ***	− 17.43
Female	48.64	60.70 ***	+ 12.05	70.19 ***	+ 21.55	66.07 ***	+ 17.43
**Race/ethnicity**							
White	62.54	66.69 ***	+ 4.15	68.17 ***	+ 5.63	71.95 ***	+ 9.41
Hispanic	17.15	12.28 ***	− 4.87	11.60 ***	− 5.55	10.47 ***	− 6.67
Black	11.46	11.40 ^ns^	− 0.05	8.84 ***	− 2.61	9.30 ***	− 2.16
Asian	6.06	6.09 ^ns^	+ 0.03	8.39 ***	+ 2.33	4.06 ***	− 1.99
Others	2.80	3.54 ***	+ 0.74	3.00 **	+ 0.20	4.21 ***	+ 1.41
**Relationship status**							
In a relationship	60.42	58.88 ***	− 1.55	61.65 ***	+ 1.23	59.03 ***	− 1.40
Not in a relationship	39.58	41.12 ***	+ 1.55	38.35 ***	− 1.23	40.97 ***	+ 1.40
**Education**							
Less than high school	10.60	4.42 ***	− 6.18	2.58 ***	− 8.02	3.63 ***	− 6.97
High school, some college	56.55	47.00 ***	− 9.55	41.25 ***	− 15.30	43.30 ***	− 13.25
Bachelor	20.33	27.19 ***	+ 6.86	32.39 ***	+ 12.06	28.64 ***	+ 8.31
Master or higher	12.52	21.39 ***	+ 8.87	23.78 ***	+ 11.27	24.42 ***	+ 11.91
**Region**							
West	23.79	28.99 ***	+ 5.20	27.04 ***	+ 3.25	30.69 ***	+ 6.90
Northeast	17.43	17.33 ^ns^	− 0.09	19.44 ***	+ 2.01	16.83 ^ns^	− 0.59
Midwest	20.77	20.79 ^ns^	+ 0.02	22.14 ***	+ 1.37	22.55 ***	+ 1.77
South	38.01	32.88 ***	− 5.12	31.37 ***	− 6.63	29.93 ***	− 8.08
**Health status**							
Excellent	21.73	22.53 ***	+ 0.80	31.00 ***	+ 9.27	22.28^ ns^	+ 0.55
Very good	34.50	39.23 ***	+ 4.74	41.54 ***	+ 7.05	36.41 ***	+ 1.91
Good	29.46	26.57 ***	− 2.89	21.42 ***	− 8.03	29.11^ ns^	− 0.34
Fair	11.04	9.37 ***	− 1.67	5.19 ***	− 5.85	10.07 ***	− 0.97
Poor	3.27	2.30 ***	− 0.98	0.84 ***	− 2.43	2.13 ***	− 1.14
**Saw mental health professional? (past 12 months)**							
Yes	12.66	26.26 ***	+ 13.61	22.73 ***	+ 10.07	34.94 ***	+ 22.28
No	87.34	73.74 ***	− 13.61	77.27 ***	− 10.07	65.06 ***	− 22.28
**Psychological distress (K6)**							
No/Mild distress	82.92	75.40 ***	− 7.52	80.96 ***	− 1.96	68.36 ***	− 14.56
Moderate distress	12.12	16.95 ***	+ 4.82	13.97 ***	+ 1.84	21.34 ***	+ 9.22
Severe distress	4.95	7.65 ***	+ 2.70	5.07^ ns^	+ 0.12	10.30 ***	+ 5.35
**Physical activity**							
Inactive	61.99	49.34 ***	− 12.65	35.88 ***	− 26.11	47.30 ***	− 14.69
Yearly exercise	1.45	1.92 ***	+ 0.48	1.65 ***	+ 0.20	1.54^ ns^	+ 0.09
Monthly exercise	6.15	7.70 ***	+ 1.55	8.62 ***	+ 2.47	7.81 ***	+ 1.66
Weekly exercise	27.24	37.39 ***	+ 10.15	49.59 ***	+ 22.35	39.59 ***	+ 12.35
Daily exercise	3.17	3.65 ***	+ 0.47	4.27 ***	+ 1.09	3.76 ***	+ 0.59
**Weight status**							
Healthy weight	31.32	35.70 ***	+ 4.38	44.70 ***	+ 13.39	36.27 ***	+ 4.95
Underweight	1.68	1.76 ^ns^	+ 0.08	1.96 ***	+ 0.28	2.02 ***	+ 0.34
Overweight	33.76	32.24 ***	− 1.52	32.15 ***	− 1.61	30.60 ***	− 3.16
Obese	33.25	30.29 ***	− 2.95	21.19 ***	− 12.05	31.11 ***	− 2.13
**Smoking status**							
Non-smoker	66.00	65.79 ^ns^	− 0.21	72.08 ***	+ 6.07	63.63 ***	− 2.37
Former smoker	22.47	24.30 ***	+ 1.83	21.31 ***	− 1.15	25.65 ***	+ 3.18
Current, some days	2.69	3.10 ***	+ 0.41	2.67^ ns^	− 0.02	3.30 ***	+ 0.61
Current, daily	8.84	6.81 ***	− 2.04	3.95 ***	− 4.90	7.42 ***	− 1.42
**Alcohol status**							
Lifetime abstainer	13.38	8.73 ***	− 4.65	8.50 ***	− 4.88	7.15 ***	− 6.23
Former drinker	17.22	16.99 ^ns^	− 0.23	9.96 ***	− 7.26	15.85 ***	− 1.38
Current drinker	69.39	74.27 ***	+ 4.88	81.54 ***	+ 12.14	77.00 ***	+ 7.61

Within each sociodemographic and health characteristic subgroup, Chi-square tests of independence compared the percentage of each of the three CAM practice subgroup populations (% Meditation users, % Yoga users, and % Guided imagery/progressive relaxation users) to non-users in that subgroup. While statistical comparisons were against non-user groups, subpopulation estimates (%) and differences between US population and user population estimates (Δ %) are provided as the basis of comparison for ease of interpretation. All estimates are weighted and age-adjusted to the standard 2000 US population^74^. Significance was set at false discovery rate (FDR) adjusted *p* < 0.05 using the Benjamini–Hochberg method. ***: *p* < 0.001; **: *p* < 0.01; *: *p* < 0.05; ^ns^: not significant at *p* < 0.05. Source: NHIS Data 2002–2022.

For health characteristics, the most represented subgroups among meditation users in 2022 had better health (very good, excellent), accessed mental health services, moderate or severe distress (indicating a probable moderate or severe mental health diagnosis), were active (exercised yearly, monthly, weekly, daily), healthy weight, and were former or current ‘some day’ smokers, and current drinkers. The least represented groups had worse health (good, fair, or poor), did not access mental health services, no/mild psychological distress, were inactive, overweight, or obese, and were current daily smokers, and lifetime abstainers from alcohol. Meditation use was not significantly different to the US population for underweight individuals, non-smokers, and former drinkers.

#### Yoga

The most highly represented sociodemographic subgroups among yoga users in 2022 were younger (18–24, 25–34), female, White, Asian, or ‘Other’ race, in a relationship, more educated (bachelor, master or higher) and residing in the West, Northeast or Midwest (Fig. 1, Table 1, Table S6). The least represented groups were middle-to-older-aged (35–44, 45–64, 65 +), male, Hispanic or Black race, not in a relationship, less educated (less than high school, high school) and residing in the South.

For health characteristics, the most represented subgroups among yoga users in 2022 had better health (excellent, very good), accessed mental health services, moderate distress, were active (exercised yearly, monthly, weekly, daily), healthy weight or were underweight, and were non-smokers and current drinkers. The least represented groups had worse health (good, fair, poor), did not access mental health care, were inactive, overweight, or obese, former or current daily smokers, and former drinkers or lifetime abstainers. Yoga use was not significantly different to the US population for users with severe psychological distress and current ‘some day’ smokers.

#### Guided imagery/progressive relaxation

The most highly represented sociodemographic subgroups among guided imagery/progressive relaxation users in 2022 were 25–34 and 45–64 years old, female, White or ‘Other’ race, not in a relationship, more educated (bachelor, master or higher), and residing in the West or Midwest (Fig. 1, Table 1, Table S6). The least represented groups were 18–24 and 35–44 years old, male, Hispanic, Black or Asian race, in a relationship, less educated (less than high school, high school) and resided in the South. Guided imagery/progressive relaxation use was not significantly different to the US population for 65 + year olds and users residing in the Northeast.

For health characteristics, the most represented subgroups among guided imagery/progressive relaxation users in 2022 had better health (very good), accessed mental health services, moderate or severe distress, were active (exercised monthly, weekly, or daily), healthy weight or were underweight, and were former or current (‘some day’ or daily) smokers, and current drinkers. The least represented groups had worse health (fair, poor), did not access mental health care, no/mild psychological distress, were inactive, overweight, or obese, were non-smokers or current daily smokers, and former drinkers or lifetime abstainers. Guided imagery/progressive relaxation use was not significantly different to the US population for users with ‘good’ health and those who exercised yearly.

### Changes in the sociodemographic and health profiles of meditation, yoga, and guided imagery/progressive relaxation users from 2002–2022

Deviation coded contrasts (Table S4) revealed broadly similar patterns across the 20 years to those observed in 2022 alone, suggesting the sociodemographic and health profiles of meditation, yoga, and guided imagery/progressive relaxation users were largely stable between 2002 and 2022 (see Tables S4–S5 for more detail).

**Figure 2:**
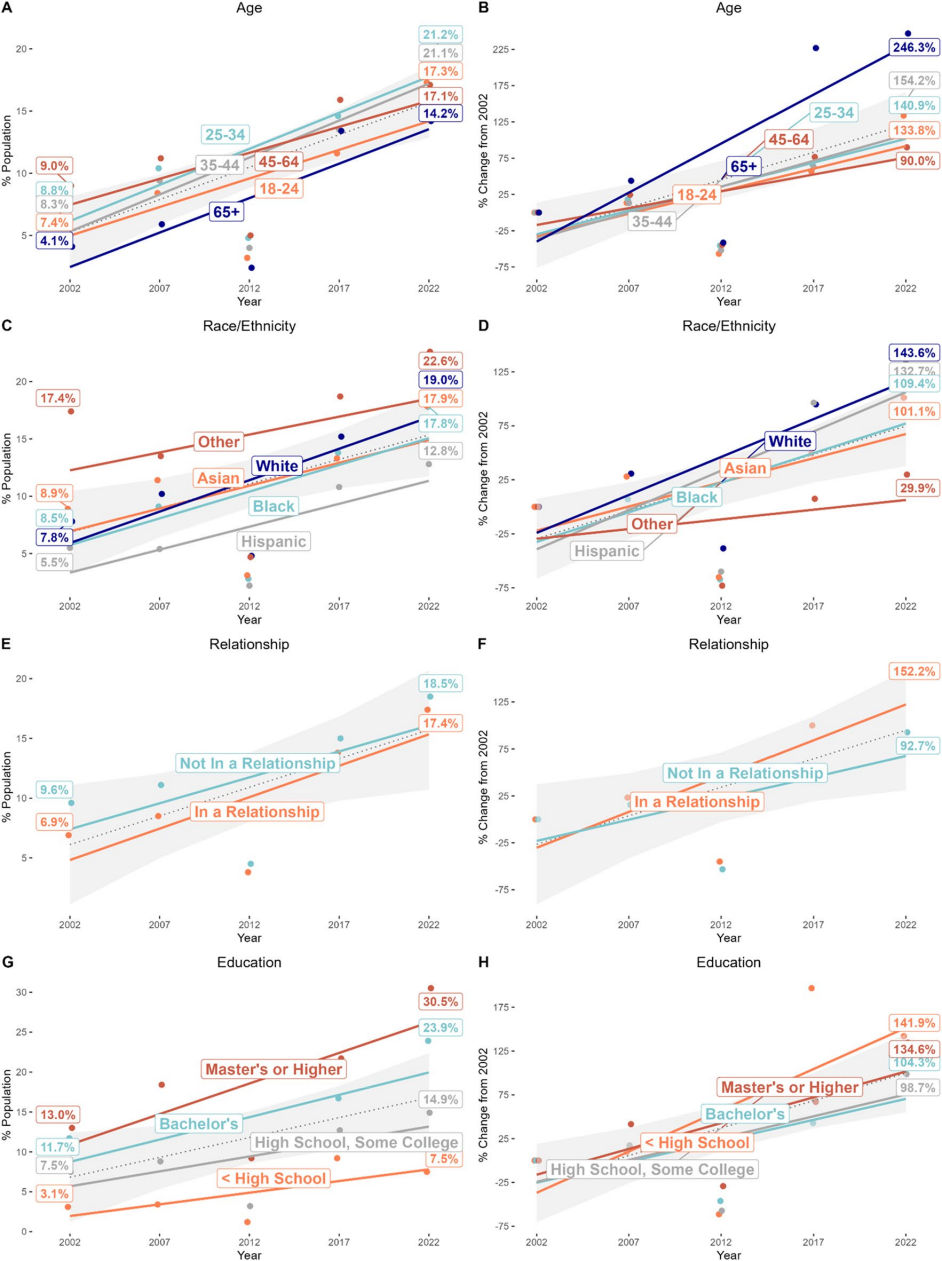
Changes in the population prevalence and rate of growth in meditation by different sociodemographic subgroups between 2002–2022. Left panels show changes in weighted population estimates (dots, percentage labels) and regressed growth rates (solid lines) of meditation by each age, race/ethnicity, relationship status and educational attainment user subgroup compared to the grand average (dashed line with error shading, representing 95% confidence intervals) between 2002 and 2022. Right panels show percent changes from 2002 at each timepoint (dots, percentage labels; 2007, 2012, 2017, 2022) and regressed rates of change in growth (solid lines) for the same user subgroups compared to the grand average (dashed line with error shading, representing 95% confidence intervals). Source: NHIS Data 2002–2022.

For meditation, characteristic x time interactions for age, relationship, education, mental health care, smoking status, and alcohol status (i.e., characteristics with a significant omnibus interaction, Table S4) revealed the subgroups showing faster than average uptake of meditation over the past 20 years were 65 + year olds (OR = 4.22), people in a relationship (OR = 1.36), people with less than high school education (OR = 4.02; Fig. 2), people who had not seen a mental health professional in the past 12 months (OR = 1.39), non-smokers (OR = 2.15), or lifetime abstainers from alcohol (OR = 2.29). Contrasts also revealed significantly slower than average uptake of meditation over the past 20 years for users who were not in a relationship (OR = 0.74), held a Bachelor level degree (OR = 0.46; Fig. 2), had seen a mental health professional in the past 12 months (OR = 0.72), or who were current ‘some day’ smokers (OR = 0.40) or current drinkers (OR = 0.58). No significant interactions were observed for gender, race/ethnicity, region, health status, psychological distress, physical activity, or weight status, indicating no notable changes in rates of use over time by any subgroups for these characteristics. Despite some significant characteristic x time interactions for yoga (Table S4), there were no significant omnibus interactions for either yoga or guided imagery/progressive relaxation (Table S4), indicating no significant shifts in sociodemographic or health profiles for these practices. Figures and Tables for all sociodemographic and health characteristics for meditation (Figure S1, Tables S1, S4), yoga (Figure S2, Tables S2, S4), and guided imagery/progressive relaxation (Figure S3, Tables S3, S4) are available in Supplemental Material.

## Discussion

Our findings suggest that interest in CAM practices has grown massively across most sociodemographic strata, with meditation and guided imagery/progressive relaxation growing two-fold, and yoga three-fold between 2002 and 2022. Approximately 1 in 5 (60.53 M) American adults used meditation and 1 in 6 (55.78 M) used yoga in 2022. Guided imagery/progressive relaxation was less popular, used by around 1 in 15 (22.22 M). While growth in meditation use has been strong over the past 20 years, our analysis using 5 timepoints revealed a more stable rate of growth than the previously most recent estimates which suggested three-fold growth between 2012–2017^5^ alone. Increases in accessibility^52^, evidence regarding health and wellbeing benefits^10–12^, and barriers to accessing conventional mental health services^38,39^ may be contributing to increases in use across all practices. The widespread accessibility of meditation apps may explain why growth in meditation use has been stronger than for yoga and guided imagery/progressive relaxation. The use of^32,33,53^, and research into^31^ meditation apps has increased sharply in recent years, with recent cross-sectional data suggesting that between 58–75% of meditators in the US have used a meditation app at least once, with 21–23% using them regularly^43,45,52^.

Women, younger-to-middle-aged people, those residing in the West and privileged groups including Whites and more educated individuals were overrepresented in all three practices in 2022, a pattern that hasn’t changed markedly across 20 years^4,5,46^. Importantly however, the user profiles presented here are somewhat different to previous reports^3–8^ that have considered the most prevalent user groups at a whole population (rather than a subgroup) level. Whole population estimates are biased toward the largest sociodemographic groups as per US Census data; i.e., older White women who are high school educated and reside in the South^51^. While it is true that there are numerically more of this type of user than other types, our report shows that 45–64 year-olds and residents of the South, while highly prevalent, are in fact slightly underrepresented among meditation and yoga users when compared to other ages and regions.

Overall, there is a lack of clear data that might explain why certain sociodemographic groups remain overrepresented across meditation, yoga, and guided imagery/progressive relaxation practices over time. A recent study using 2012 NHIS data to explore gender differences in meditation use found that women used every type of meditation practice (mantra, mindfulness, spiritual, and yoga-, tai chi-, qi gong- with meditation) more than men^54^. It also found more racial and ethnic variability in women who meditate relative to men^54^. Specifically, relative to White women, Black, Asian, and Hispanic women were less likely, and ‘Other’ race women more likely to mediate^54^. By contrast, only Hispanic men were less likely to meditate than White men^54^. It could be that current meditation (and other CAM) research^34^ and practice offerings aren’t sufficiently representative^55^, culturally appropriate^37,56^ and/or accessible^29^ to men, or people of color broadly, though this pattern of results could equally represent disparities in employment or caring duties and other life pressures in these groups^29^. For example, women with high income are more likely than men with high income to meditate^54^, and African American women cite care and work obligations as barriers to accessing meditation offerings^29^. More research is needed to understand the cultural and socioeconomic factors that drive these and other sociodemographic differences.

NHIS 2017 data suggest the two strongest predictors of meditation use are: 1. the presence of a health problem, and 2. cost or access barriers to conventional medical care^6^. The use of meditation- and other mental health apps has grown significantly in recent years^32,33^, alongside a US mental healthcare system that has been described as being ‘in crisis’ with significant levels of unmet need^38,39^. Almost a third (28.2%) of the more than 50 million (20.8%) American adults experiencing mental illness, report being unable to access the treatment they needed due to cost and access issues^57^. Consistent with this, we found several indications in the data that overrepresentation, or faster-than-average growth in use, may reflect unmet healthcare needs in some subgroups. Perhaps the strongest indicator, over the past 20 years, the number of meditators who had not seen a mental health professional in the past 12 months grew 39% faster than average (and those who did see a mental health professional 28% slower than average), despite no commensurate reductions in psychological distress. For race/ethnicity, the ‘Other’ race category, which predominantly comprises Indigenous peoples, was overrepresented (albeit from a small total population) in all 3 practices. The higher uptake of meditation and guided imagery/progressive relaxation among Indigenous Americans may reflect the identification of traditional or revitalized cultural and spiritual practices^58^ as ‘meditation’, but may also reflect disparate unmet needs for health services^59^. Similarly, for age, meditation use grew 146% faster than average for 65 + year olds which, despite controlling for demographic shifts with age adjusting, may reflect a large cohort of 45–64 year-olds getting older and continuing practices established earlier in life^60^. Older adults use more, and different, health services than younger adults, the demand for which is projected to put pressure on the capacity for the health system to deliver those services^61^. Thus the substantially faster uptake in meditation may also reflect the significant scale of unmet health needs among older adults looking for ways to manage age-related health and wellbeing^62,63^. For education, meditation use grew 41% faster than average among those with less than high school education, which may signal increasing exposure to a growing number of school-based meditation programs^64,65^, but may also reflect higher unmet health needs among those of lower socioeconomic status^66^. Given the possible links between socioeconomic disadvantage and self-guided training via apps with impairing adverse events^42^, it is critical that adequate supports are in place for vulnerable user groups such as these.

Our findings are consistent with other data suggesting that physical health, mental health, and wellbeing are leading motivators for the use of meditation, yoga, and guided imagery/progressive relaxation^67–70^. Individuals with better health status (very good or excellent), who were active, and had healthy weight were overrepresented in all three practices. However, good health may be a predictor, confounder, and/or consequence of practice. For meditation, non-smoking (115% faster), and non-drinking (i.e., lifetime abstainer; 129% faster) users grew faster than the average rate of growth over the past 20 years, which taken together with slower than average growth in meditators who were current drinkers (42% slower), may suggest that some individuals are engaging in meditation practice as an adjunct to other healthy lifestyle choices^71^. However, individuals with moderate or severe psychological distress (indicative of a probable mental health diagnosis) and those who had seen a mental health professional in the past year were also overrepresented across the three practices, suggesting a distinct group of individuals who may use CAM practices to help manage mental illness and promote mental health^38,71^. Notably, nearly as many American adults used meditation in 2022 (18.3%) as saw a mental health professional (23%)^72^, with our data indicating the rate at which meditators access mental health care is slowing substantially. The extent to which this reflects met vs. unmet need warrants further investigation.

Despite the documented benefits of meditation and yoga to mental health and wellbeing^10–13^, they encompass a wide variety of approaches that are not equivalent, and there is significant variability in the availability and quality of evidence for different practices (see e.g., Van Dam et al., 2018 for discussion of meditation^21^). Currently, there is insufficient evidence for meditation, yoga, and guided imagery/progressive relaxation to make specific recommendations about who is most likely to benefit from which practice or for which problem, how barriers to access can be reduced for underrepresented groups, how specific practices may interact with other interventions, or which practices may be contraindicated for certain individuals or groups^41–43^. Given the large proportion of the US population using meditation, yoga, and guided imagery/progressive relaxation, there is a critical need for targeted research to build this evidence, and to understand the risk–benefit ratio of these practices to inform clinical care. However, in the absence of such evidence, it is essential that clinicians ask their patients exactly what they do and how they do it, to enable treatment planning, consultation (where appropriate), and risk–benefit monitoring.

The main strength of this study is the use of logistic regression with deviation contrasts averaging across the 5 timepoints to understand prevalence and trends over 20 years. While this approach reduced the influence of anomalous 2012 data (which exaggerated previous growth estimates^5^) and increased our confidence in the robustness of the observed interactions, it was also less sensitive to short-term changes, potentially limiting investigation of recent practice trends. Another limitation, since the data are cross-sectional, we were unable to examine causality. Notably, due to changes in the NHIS CAM supplement over the past 5 cycles, we were also limited in the range of sociodemographic and health variables that we could investigate across the 20 year period, and data were not available on important variables such as socioeconomic status, urbanicity/rurality, and *how* individuals access CAM practices (e.g., in person vs. via mobile apps) that might help explain the pattern of results in more detail. Nonetheless, these findings highlight important subgroup prevalence findings, and temporal trends that have not been evident in prior publications.

In conclusion, a significant number of American adults engage in meditation and yoga with trends indicating these numbers will likely continue to grow. Growth in meditation use has been significantly faster among some underrepresented sociodemographic subgroups, potentially reflecting unmet mental health needs. Further efforts are needed to explore how meditation, yoga, and guided imagery/progressive relaxation, either as standalone interventions or adjuncts, may be adapted and utilized among underrepresented sociodemographic groups to ensure equity, cultural appropriateness, accessibility, effectiveness, and safety for the various patient subpopulations who use them.

## Methods

### Study design and data source

NHIS is an annual cross-sectional interview survey assessing sociodemographic and health characteristics of non-institutionalized civilian US residents^49^ that uses multistage probability sampling to achieve national representativity^49,50^. The present study is a secondary analysis of sociodemographic and health characteristics related to complementary practices surveyed within the CAM supplement (i.e., meditation, yoga, guided imagery, and progressive relaxation) every 5 years over the span of 20 years (2002–2022). Because this study used publicly available deidentified data, we did not preregister the study and were exempt from human ethics review.

### Variables

The NHIS *Family* and *Sample Adult* core surveys collected data on a range of sociodemographic and health characteristics^73^. Data on contemplative practices were collected in the *CAM* supplement, with inclusion of only those variables that were consistently collected in all cycles.

*Sociodemographic characteristics* included: sex, age (collapsed to recommended categories for age-adjustment weighting^74^), race/ethnicity, relationship status, education, and region of residence (collapsed to categories^75^).

*Health characteristics* included: self-perceived overall health status, mental health care access (past 12 months), psychological distress (Kessler Psychological Distress Scale—6 item, K6^76^), physical activity, weight, smoking and alcohol consumption. In 2022, the K6 was replaced by a depression measure: the Patient Health Questionnaire (PHQ8)^77^. To compare changes in psychological distress across 20-years, we converted PHQ8 scores to K6 score equivalents^78^.

*Complementary practices* included questions relating to use of meditation, yoga, guided imagery, and progressive relaxation in the past 12 months (see Supplemental Material). Prior to 2022 guided imagery and progressive relaxation were considered as separate questions, so data from the 2002–2017 CAM supplements were collapsed to enable comparison).

### Statistical analysis

All analyses were conducted between July and December 2023 using R Studio version 2023.06.1 (R base 4.2.2). Details of data preparation and analyses are provided in Supplemental Material.

*Prevalence estimates and trends.* Prevalence estimates (calculated as both percentages (%) and population (N) estimates) for the use of meditation, yoga, and guided imagery/progressive relaxation were calculated at a population level, both for sociodemographic and health characteristics (e.g., age), and user subgroups (e.g., 18–24 year olds) in each survey year. All prevalence estimates were weighted in the *survey* R package^79^ using the weights provided by NHIS, calibrated to the 2000 census-based population estimates for age, gender, and race/ethnicity, to yield standardized nationally representative prevalence estimates^74^. Weighted logistic regressions were then used to estimate growth rates in the prevalence of the use of these practices between 2002–2022 at a population level, sociodemographic or health characteristic level, as well as at a sociodemographic or health subgroup level. Regressed growth rates are expressed as both β coefficients (i.e., % growth per 5 year interval; see Supplemental Material; False Discovery Rate (FDR) adjusted significance using the Benjamini–Hochberg method set at *p* < *0.05*) and annual growth rates (i.e., β/5). All main regressions were included in one FDR adjustment. Chi-square tests of independence comparing weighted prevalence estimates were used to evaluate differences in the use of each of the three practices by each sociodemographic and health characteristic subgroup relative to non-users within that subgroup, with significance set at *p* < 0.05 following a second FDR adjustment (i.e., for all Chi-square tests only) as described above.

*Differences in growth rates over time.* For each of the three practices: 1. a set of regression models with two predictors (characteristic and time) were run to understand how belonging to that characteristic subgroup predicted engagement in the practice while controlling for time. Then, 2. for each model, a characteristic x time interaction term was added to understand how engagement changed over time. Omnibus tests were conducted for models in 1. and 2., with significance set at *p* < 0.05 following a third FDR adjustment (i.e., for all F-tests only) as described above. Upon significant omnibus results, *post hoc* analyses were undertaken to examine whether linear patterns in engagement varied as a function of subgroup membership using the *emmeans* package^80^, employing deviation contrast coding to compare subgroups to the mean across all subgroups while averaging across time. Significance for *post hoc* tests was set at *p* < 0.05 and Bonferroni corrected to account for multiple comparisons.

### Supplementary Information

Supplementary Information.

## Data Availability

Data used in this study is publicly available at https://www.cdc.gov/nchs/nhis/. R code and other materials related to this study are available on the Open Science Framework at https://osf.io/c3wyt.
