# Supplementary material for: Prevalence and 20-year trends in meditation, yoga, guided imagery and progressive relaxation use among US adults from 2002 to 2022
Source: Sci Rep. 2024 Jul 1;14:14987. doi: 10.1038/s41598-024-64562-y (PMC11217305; doi:10.1038/s41598-024-64562-y)
Supplement: Supplementary file 1 — Supplementary Information. [file 41598_2024_64562_MOESM1_ESM.docx]

**Supplemental Material for**

**Prevalence and 20-year Trends in Meditation, Yoga, Guided Imagery and Progressive Relaxation Use Among US Adults From 2002 to 2022**

**Running title (45 char):** US complementary practice trends 2002 to 2022

Jonathan N Davies, PhD^1^, Anna Faschinger, MSc ^1^, Julieta Galante, PhD ^1^ & Nicholas T Van Dam, PhD ^1^

^1^Contemplative Studies Centre, Melbourne School of Psychological Sciences, University of Melbourne

**Corresponding author:**

Nicholas T Van Dam

Contemplative Studies Centre

Melbourne School of Psychological Sciences

Faculty of Medicine, Dentistry, and Health Sciences

Level 1, Melbourne Connect

700 Swanston Street

The University of Melbourne, VIC 3010 Australia

Tel: +61 4 3447 4929

Email: nicholas.vandam@unimelb.edu.au

**Keywords:** Meditation; Yoga; Guided Imagery; Progressive Relaxation; Public health; NHIS; National Health Interview Survey; Prevalence; Trend; CAM; Complementary and Alternative Medicine;

**Data sharing statement:** Data used in this study is publicly available at <https://www.cdc.gov/nchs/nhis/>. R code and other materials related to this study are available on the Open Science Framework at <https://osf.io/c3wyt>

**Conflict of interest statement:** The authors have no conflicts of interest to declare. The manuscript from this study has not been submitted or published elsewhere.

**ORCID Records:**

Jonathan N Davies <https://orcid.org/0000-0001-7838-126X>

Anna Faschinger <https://orcid.org/0009-0002-1788-6453>

Julieta Galante <https://orcid.org/0000-0002-4108-5341>

Nicholas T Van Dam <https://orcid.org/0000-0002-1131-0739>

**Table of Contents:**

[Supplemental Methods 4](#_Toc165379110)

[Data source and preparation (see also main Methods) 4](#_Toc165379111)

[Variables (see also main Methods) 4](#_Toc165379112)

[Prevalence estimates and trends (see also main Methods) 5](#_Toc165379113)

[Differences in growth rates over time. (see also main Methods) 6](#_Toc165379114)

[Supplemental Results 7](#_Toc165379115)

[Most prevalent users of meditation, yoga, and guided imagery/progressive relaxation at a whole population level 7](#_Toc165379116)

[20-year Trends 7](#_Toc165379117)

[Supplemental Figures 11](#_Toc165379118)

[Figure S1. Changes in the population prevalence and rate of engagement in meditation by different sociodemographic and health subgroups between 2002-2022 16](#_Toc165379119)

[Figure S2. Changes in the population prevalence and rate of engagement in yoga by different sociodemographic and health subgroups between 2002-2022 21](#_Toc165379120)

[Figure S3. Changes in the population prevalence and rate of engagement in guided imagery/progressive relaxation by different sociodemographic and health subgroups between 2002-2022 26](#_Toc165379121)

[Supplemental Tables 27](#_Toc165379122)

[Table S1. Population prevalence and 20-year trend in meditation (Full Data: 2002, 2007, 2012, 2017, 2022) 28](#_Toc165379123)

[Table S2. Population prevalence and 20-year trend in yoga (Full Data: 2002, 2007, 2012, 2017, 2022) 31](#_Toc165379124)

[Table S3. Population prevalence and 20-year trend in GIPR (Full Data: 2002, 2007, 2012, 2017, 2022) 34](#_Toc165379125)

[Table S4. Deviation-coded main effects and 20-year-averaged rate contrasts for meditation, yoga and guided imagery/progressive relaxation by sociodemographic and health strata (Full Data) 37](#_Toc165379126)

[Table S5. Interactions and pairwise-contrasts for meditation, yoga, and guided imagery/progressive relaxation by sociodemographic and health strata (Full Data) 40](#_Toc165379127)

[Table S6. Chi Square results comparing total population subgroup prevalence with meditation, yoga, and guided imagery/progressive relaxation subgroup prevalence (Full Data) 43](#_Toc165379128)

## Supplemental Methods

### Data source and preparation (see also main Methods)

The annual NHIS survey core includes *Household,* *Family, Person, Adult and Child* files, with the rotating complementary and alternative medicine (CAM) core (*Adult Alternative Medicine and Child Alternative Medicine)* every 5 years*.* More information on NHIS survey composition and sampling strategy can be found at <http://www.cdc.gov/nchs/nhis/about_nhis.htm>. In 2022, NHIS interviews were completed in 28,854 households (total household response rate 49.6%), which yielded 27,651 adults (final sample adult response rate 47.7%).

The main data preprocessing steps included: 1. recoding and renaming relevant variables; 2. Merging *Person, Adult* and *Adult Alternative Medicine* files and reducing to variables of interest; 3. Recoding all “Refused”, “Don’t Know” and “Not Ascertained” responses as missing values; and 4. Recoding 12 month use (of meditation, yoga, guided imagery/progressive relaxation) variables as “No” where the relevant lifetime use variable was answered “No” (leading to missing data for the 12 month use question).

Following preprocessing, factors were created for all variables as described in the main manuscript. Specific factor creation differed slightly for the following variables: 1. age (creating age categories), 2. race/ethnicity (merging two variables), 3. relationship status; 4. psychological distress (crosswalking PHQ8 scores (2022) to K6 scores then scoring and recoding K6 scores based on clinical cutoffs); and 5. weight status (based on BMI scores). Guided imagery and progressive relaxation were merged into one variable (2002, 2007, 2012 and 2017 only); and mantra, mindfulness and spiritual meditation were also merged into one variable (2012 and 2017 only). At this point, data were saved as smaller data files for future use.

### Variables (see also main Methods)

***Contemplative practices*** included questions relating to use of meditation, yoga, guided imagery, and progressive relaxation in the past 12 months (see Supplemental Material). For meditation, respondents were asked: *“Meditation includes Mindfulness, Mantra, and Spiritual meditation. In meditation a person focuses, stills, or quiets the mind. During the past 12 months, did you use any of these types of meditation?”* (note: mindfulness, mantra and spiritual meditation were considered as separate questions in 2012 and 2017 and were collapsed to enable comparison with the other 3 CAM supplements); for yoga: *“During the past 12 months, did you practice Yoga as part of a class or on your own?”*; and for guided imagery/progressive relaxation: *“Guided imagery uses visualization to relax. Progressive relaxation uses tensing and relaxing muscle groups. During the past 12 months, did you use guided imagery or progressive relaxation?”* (note: prior to 2022 guided imagery and progressive relaxation were considered as separate questions, so data from the 2002-2017 CAM supplements were collapsed to enable comparison).

### Prevalence estimates and trends (see also main Methods)

We applied population weights provided by the National Center for Health Statistics to account for the complex sampling procedure used by NHIS. A design element for each year and assigned weights were created using the *survey* package in R^50^. NHIS also recommend age-adjustment and population standardization to ensure that changes in prevalence rates over time are due to actual increases in uptake, rather than by population growth and/or demographic shifts in the data^24^. Thus, prevalence for each practice was age-adjusted using weights (distribution #9) from Klein and Schoenborn^24^ to standardize population prevalence as a function of population growth.

A sample design for all years was created using the *svydesign* function from the *survey* package. Then, age-adjusted, weighted percentages of meditation, yoga, and guided imagery/progressive relaxation users for all years were calculated using weights from <https://www.cdc.gov/nchs/data/statnt/statnt20.pdf> using the *survey* package. Percentages were calculated per age bin, then age-adjustments were added. Finally, percentages were population standardized by multiplying each percentage by 2038.51 (as per Klein et al.^24^). Due to limitations of the *survey* package, standard errors were population weighted but not age-adjusted.

Linear regressions of prevalence estimates were plotted using *Ggplot*. Absolute percentages of users within each subgroup (e.g., 7.6% of females who meditate) were calculated using *tabfreq.svy*, while relative percentages within each sociodemographic or health characteristic (e.g., meditators are 40.1% male, 59.9% female) were calculated using *svyby*.

### Differences in growth rates over time. (see also main Methods)

Interactions were also examined as a function of linear contrast (time) by pairwise contrast (category) to examine which user categories were driving effects. These results are reported in Tables S5-S6.

## Supplemental Results

### Most prevalent users of meditation, yoga, and guided imagery/progressive relaxation at a whole population level

White, 45–64-year-old females, who were in a relationship and completed high school were the most prevalent users of meditation (Figure 2, Figure S1, Table S1), yoga (Figure 3, Figure S2, Table S2), and guided imagery/progressive relaxation (Figure S3, Table S3). The most prevalent users of these practices largely had very good self-rated overall health, did not access mental health care, had no/mild psychological distress, maintained healthy weight, and were non-smokers and current drinkers. For meditation and yoga, they resided in the South, whereas for guided imagery/progressive relaxation, they resided in the West. For meditation and guided imagery/progressive relaxation, they were most commonly inactive whereas for yoga they were weekly exercisers. Of note, the profile of the most prevalent user of these practices also matches the largest sociodemographic group in the adult population as per 2020 US Census data for age (i.e., 45-64 year olds), sex (i.e., females), race/ethnicity (i.e., non-Hispanic white), relationship status (i.e., in a relationship), educational attainment (i.e., high school, some college), and region (i.e., South) as per 2020 US Census data^31^. The profile of the most common user profiles for the 3 practices were broadly consistent across years (Tables S1-S3).

### 20-year Trends

Across the 20-year period from 2002 to 2022, most sociodemographic and health subgroups for all 3 practices generally increased, excepting 2012 (Figure 1, Figures S1-S3, Tables S1-S4). Significant growth in prevalence (as expressed by regression coefficients) was found in almost all sociodemographic and health subgroups between 2002 to 2022 for all three practices, suggesting growth in engagement across most user subgroups (Tables S1-S4).

The meditation, yoga, and guided imagery/progressive relaxation subgroups that were most prevalent at a population level in 2022 also generally showed the largest 20-year numeric increases (Tables S1-S4): i.e., whites, females, those in a relationship, with high school education, residing in the South. For meditation and yoga, they were 45-64 years old while for guided imagery/progressive relaxation they were 65 years or older. For all three practices they had very good health, no psychological distress, and were non-smokers, and current drinkers. For meditation and yoga, they did not access mental health services while for guided imagery/progressive relaxation they did. For meditation and guided imagery/progressive relaxation they were inactive and overweight or obese whereas for yoga they were weekly exercises and had healthy weight. The only subpopulations that decreased (in numeric terms) over time for meditation and guided imagery/progressive relaxation were daily exercisers and current daily smokers (Figures S1-S3, Tables S1-S4).

***Meditation Engagement.*** As shown in Table S4, omnibus tests revealed significant main effects for all meditation characteristics (Fs>84.51, ps<0.001). Deviation-coded contrasts revealed that the following subgroups were most engaged in meditation over the past 20 years: 25-34 (above average: +20%) or 45-64 (+20%) years old, female (+21%), “Other” race (+53%), not in a relationship (+10%), more educated (Master or higher: +101%; Bachelor: +46%) or residing in the West (+30%). They had very good overall health (+11%), accessed mental health care (+88%), had severe (indicating probable severe mental health issues; +33%) or moderate (indicating probable moderate severity mental health issues; +21%) psychological distress, exercised weekly (+21%), maintained healthy weight (+17%), were current ’some day‘ (+30%) smokers or current (+32%) or former (+20%) drinkers.

By contrast, those who were least engaged in meditation were 65+ years old (below average: -30%), male (-17%), Hispanic (-36%), in a relationship (-9%), less educated (High school, some college: -12%; Less than high school: -61%) or resided in the South (-24%). They did not access mental health care (-47%), had no/mild psychological distress (-38%), were inactive (-42%), current daily (-15%) or non-smokers (-15%) and lifetime abstainers from alcohol (-37%).

***Meditation Trends.*** As shown in Table S4, characteristic x time interactions for age, relationship, education, smoking status, and alcohol status (i.e., characteristics with a significant omnibus interaction) revealed the subgroups showing faster than average uptake of meditation over the past 20 years were 65+ year olds (above average: +322%), people in a relationship (+36%), with less than high school education (+302%), non-smokers (+115%), or lifetime abstainers from alcohol (+129%). Contrasts also revealed significantly slower than average uptake of meditation over the past 20 years in people who were not in a relationship (below average: -26%), held a Bachelor level degree (-54%), or who were current drinkers (-42%). No significant interactions were observed for gender, ethnicity, region, health status, mental health access, psychological distress, physical activity, or weight status, suggesting comparable rates of engagement over time. Results from pairwise contrasts are shown in Table S5.

***Yoga Engagement.*** As shown in Table S4, omnibus tests revealed significant main effects for all yoga characteristics (Fs>33.76, ps<0.001). Deviation-coded contrasts revealed that the following subgroups were most engaged in yoga over the past 20 years: younger (18-24: above average: +33%; 25-34: +68%; 35-44: +25%), female (+69%), Asian (+64%) or White (+22%), more educated (Master or higher: +66%; Bachelor: +114%), or residing in the West (+29%) or Northeast (+9%). They had excellent (+41%) or very good (+79%) overall health, accessed mental health care (+52%), had moderate psychological distress (indicating probable moderate severity mental health issues; +19%), engaged in regular physical activity (daily: +35%; weekly: +69%), maintained healthy weight (+64%) or were underweight (+36%), or were current “some day” smokers (+37%), non-smokers (+15%), former smokers (+11%) or current drinkers (+86%).

By contrast, those who were least engaged in yoga were 65+ year old (below average: -62%), male (-41%), Black (-35%) or Hispanic (-40%), less educated (High school, some college: -15%; Less than high school: -79%) or residing in the South (-29%). They had fair (-38%) or poor (-64%) overall health, did not access mental health care (-34%), had severe psychological distress (indicating probable severe mental health issues; -20%). were inactive (-60%). overweight (-13%) or obese (-48%) or were current daily smokers (-43%). former drinkers (-21%) or lifetime abstainers (-32%).

No characteristic x time interactions were observed for yoga (Table S4) suggesting that there were no subgroup rates of engagement over 20 years that were significantly higher or lower than the average. Results from pairwise contrasts are shown in Table S5.

***Guided Imagery/Progressive Relaxation Engagement.*** As shown in Table S4, omnibus tests revealed significant main effects for most guided imagery/progressive relaxation (Fs>65.26, ps<0.001) characteristics except for relationship status (F=14.68, p=0.002) and weight status (F=1.58, p=0.39). Deviation-coded contrasts revealed that the following subgroups were most engaged in guided imagery/progressive relaxation over the past 20 years: young to middle aged (25-34: +13%; 35-44: +16%; 45-64: +34%) females (+32%), White (+46%) or “Other” race (+70%), not in a relationship (+9%), more educated (Master or higher: +146%; Bachelor: +59%) or residing in the West (+41%). They accessed mental health care (+118%), had severe (indicating probable severe mental health issues; +42%) or moderate (indicating probable moderate severity mental health issues; +29%) psychological distress, were weekly exercisers (+23%), maintained healthy weight (+16%), were former smokers (+13%), and current (+51%) or former (+17%) drinkers.

By contrast, those who were least likely (below the average) to engage in meditation were 65+ year olds (-41%), male (-24%). Black (-21%) and Hispanic (-40%) people, those in a relationship (-8%), less educated (High school, some college: -12%; Less than high school: -69%) or residing in the South (-30%). They did not access mental health care (-54%), had no or mild psychological distress (indicating the likelihood of no mental health diagnosis; -45%), were inactive (-45%), non-smokers (-17%), or lifetime abstainers from alcohol (-43%).

No characteristic x time interactions were observed for guided imagery/progressive relaxation (Table S3) suggesting that there were no subgroup rates of engagement over 20 years that were significantly higher or lower than the average. Results from pairwise contrasts are shown in Table S5.

## Supplemental Figures


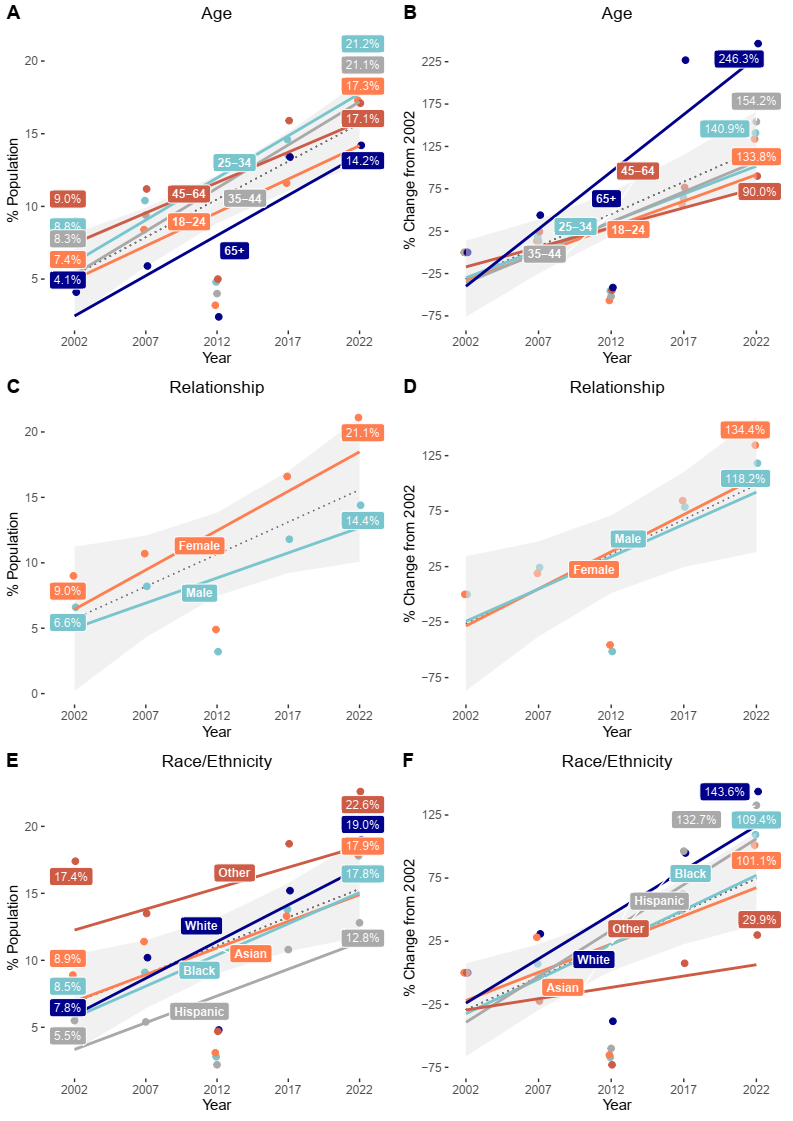


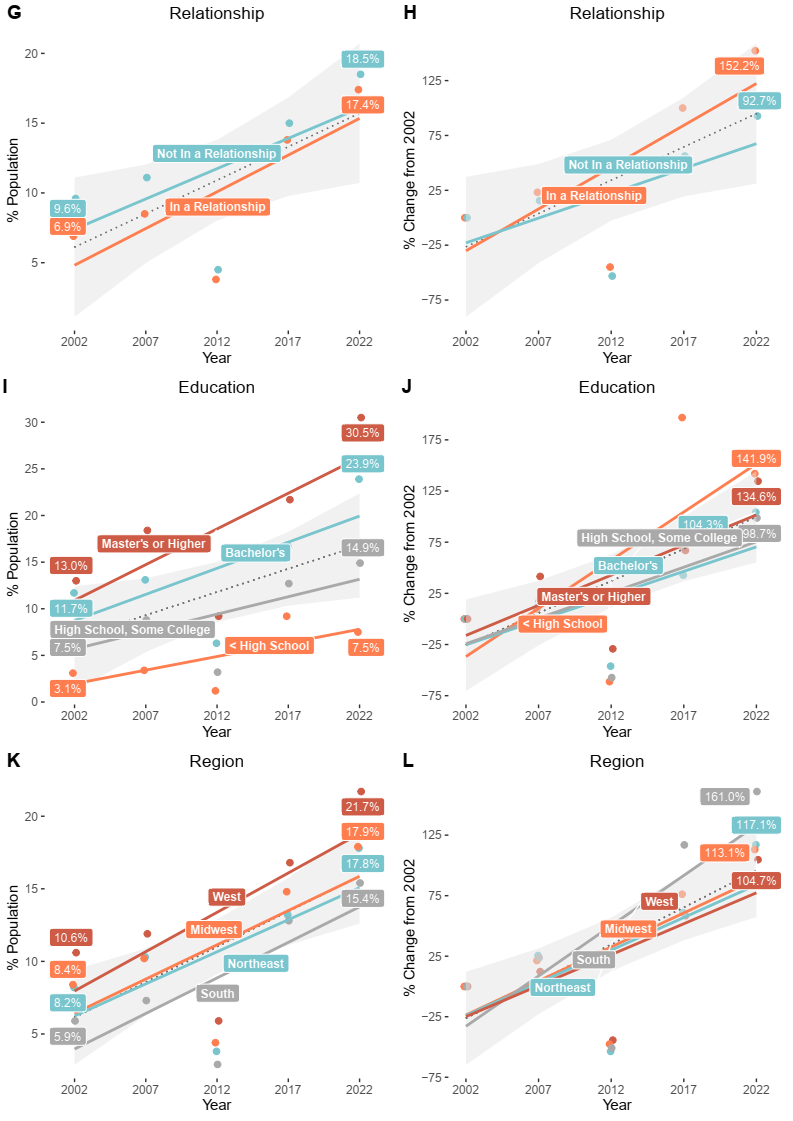


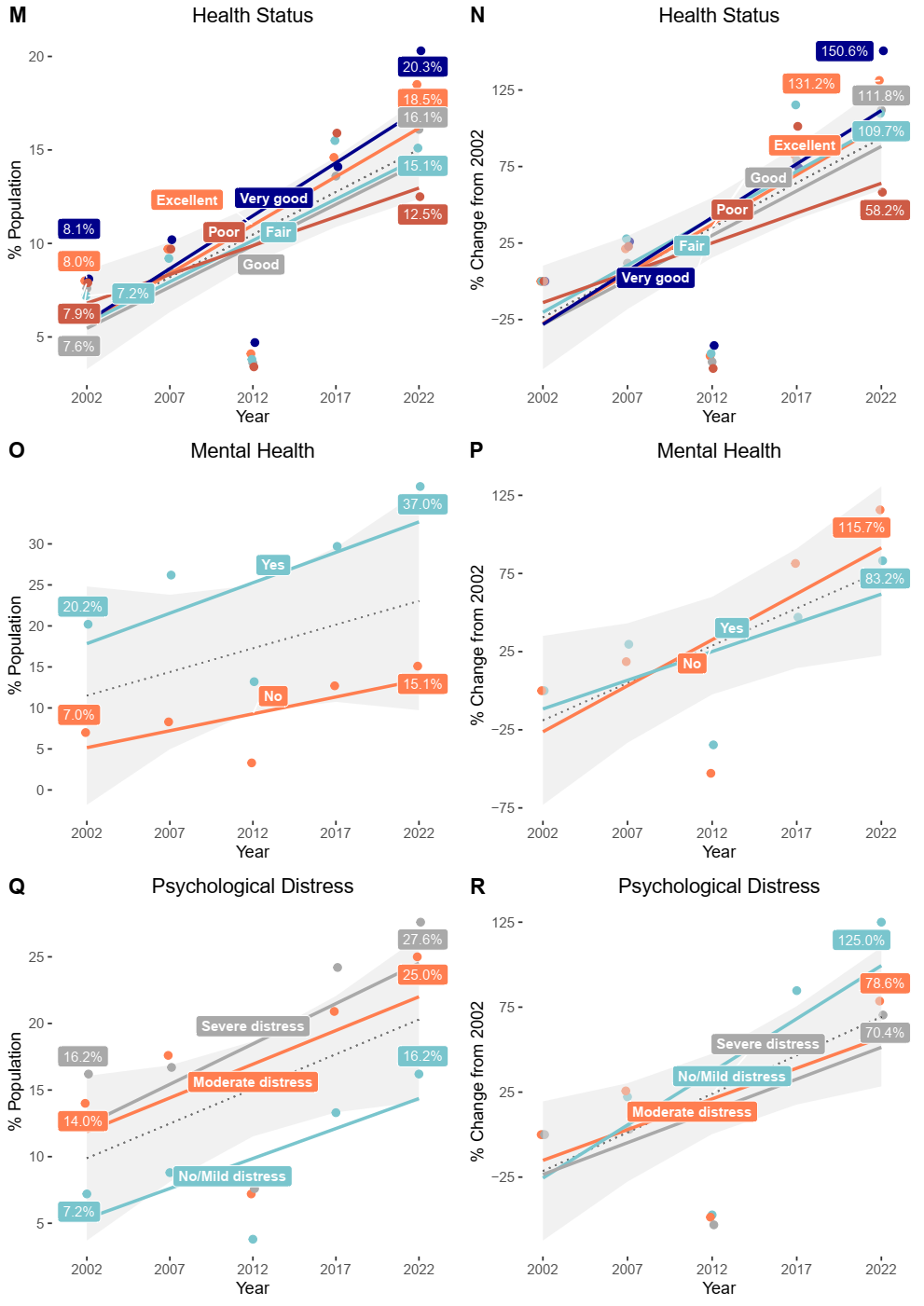


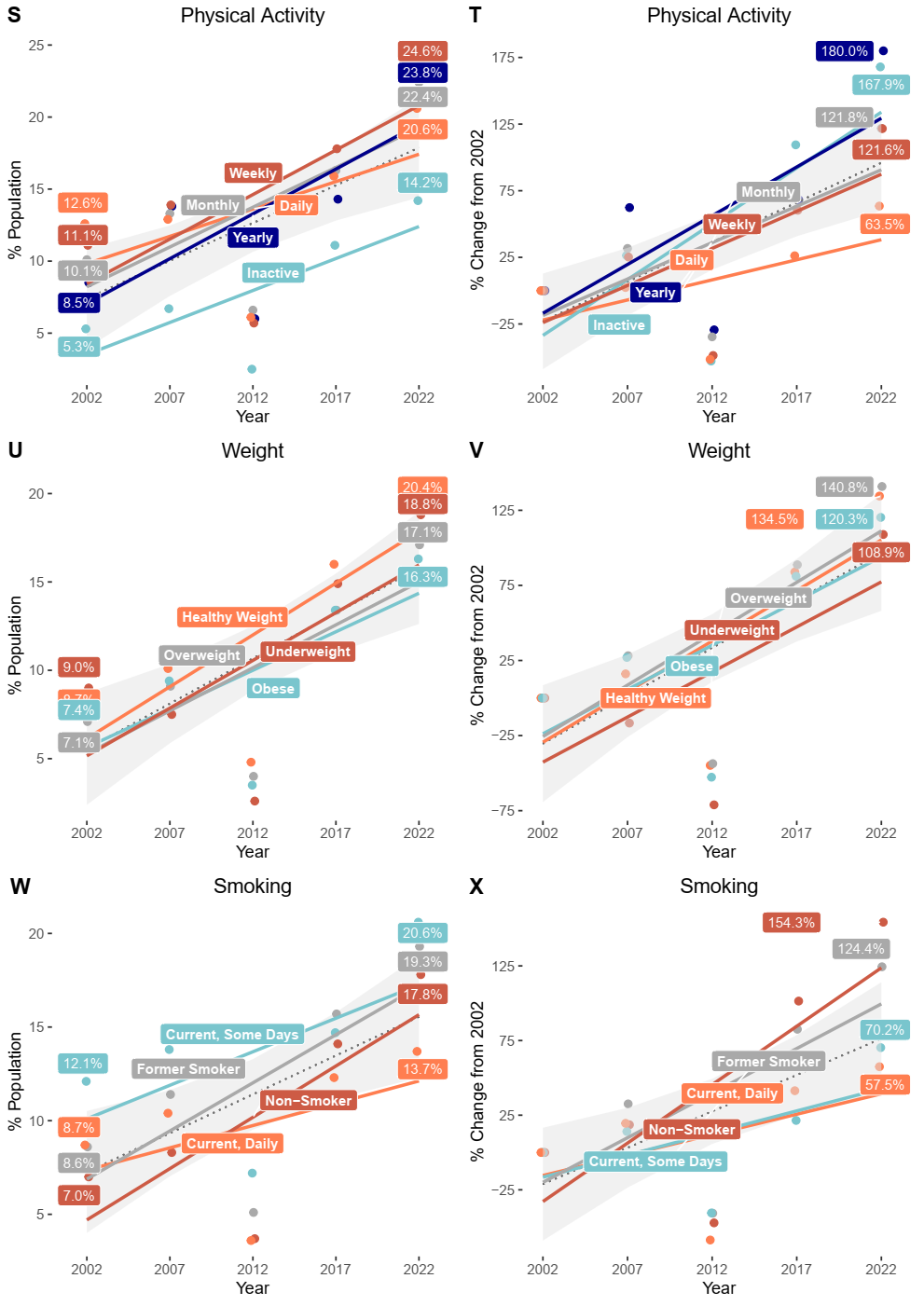


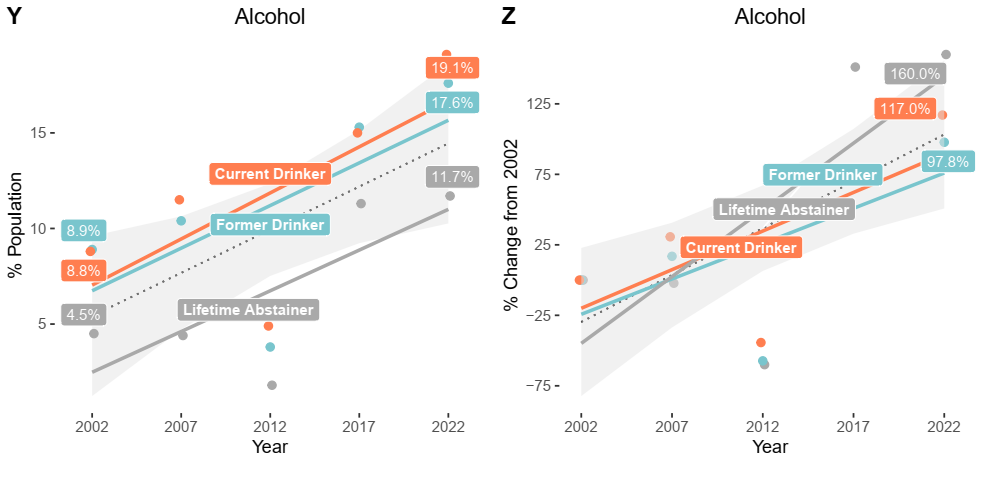


### Figure S1. Changes in the population prevalence and rate of engagement in meditation by different sociodemographic and health subgroups between 2002-2022

Left panel shows changes in the weighted population estimates (dots, percentage labels) and regressed growth rate (solid lines) of meditation by each age (A, B), sex (C, D), race/ethnicity (E, F), relationship status (G, H), educational attainment (I, J), region (K, L), health status (M, N), mental health access (O, P), psychological distress (Q, R), physical activity (S, T), weight status (U, V), smoking status (W, X) and alcohol status (Y, Z) user subgroup compared to the grand average (dashed line with error shading) between 2002 and 2022. Right panel shows change in growth rates, expressed as a percent increase from 2002 at each timepoint (dots, percentage labels; 2007, 2012, 2017, 2022) and regressed rate of change in growth solid lines) for the same user subgroups compared to the grand average (dashed line with error shading). Source: NHIS Data 2002-2022.


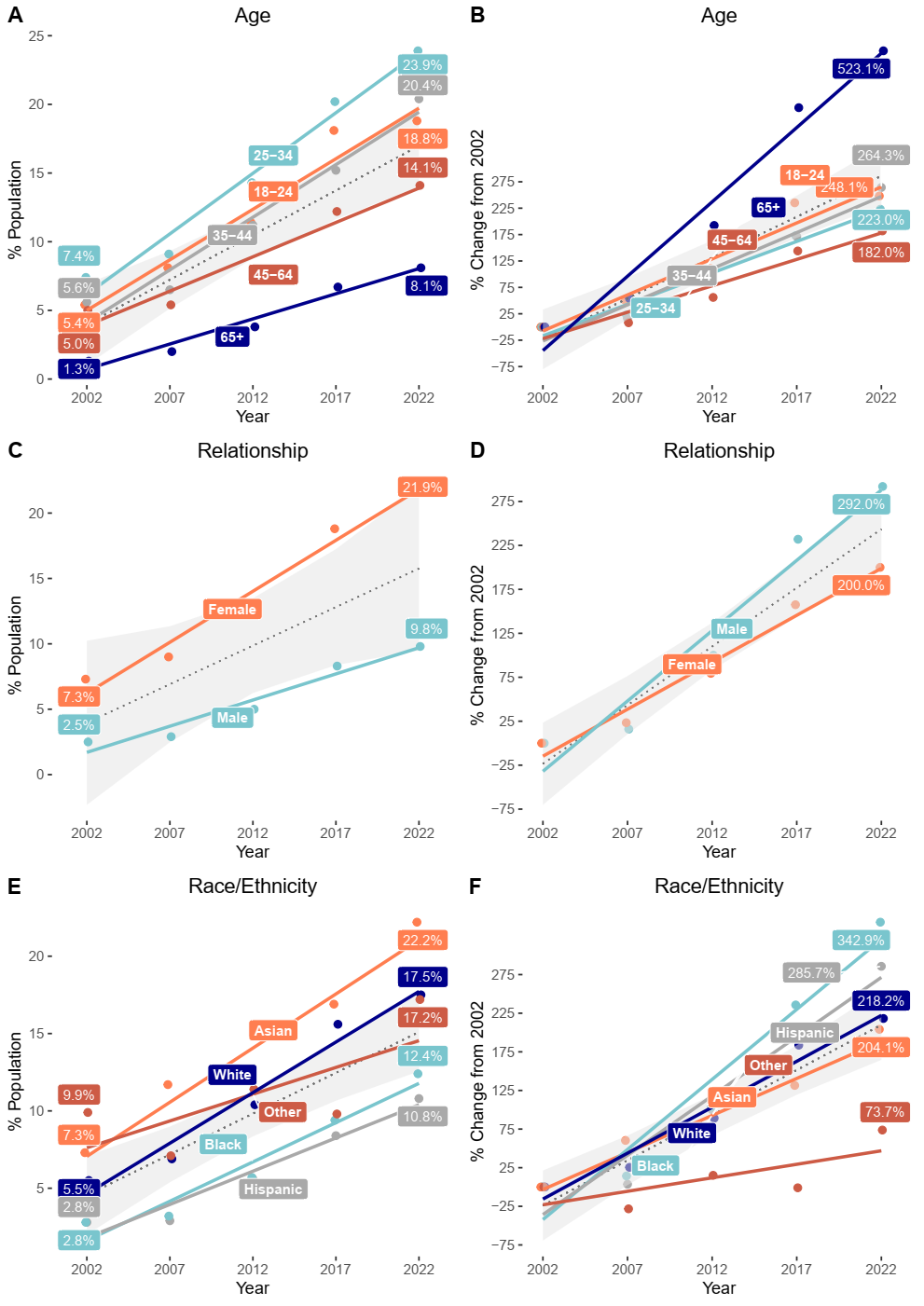


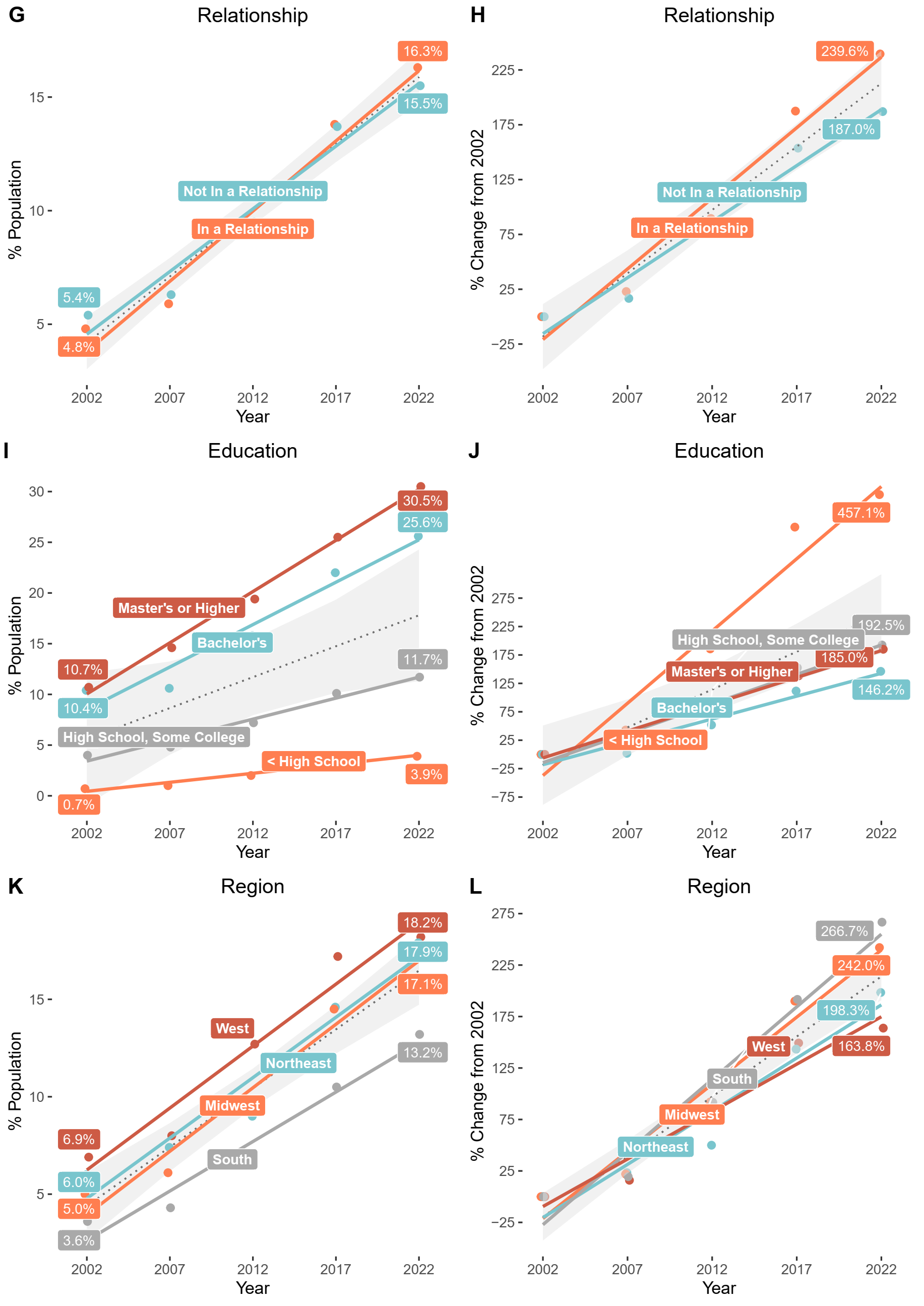


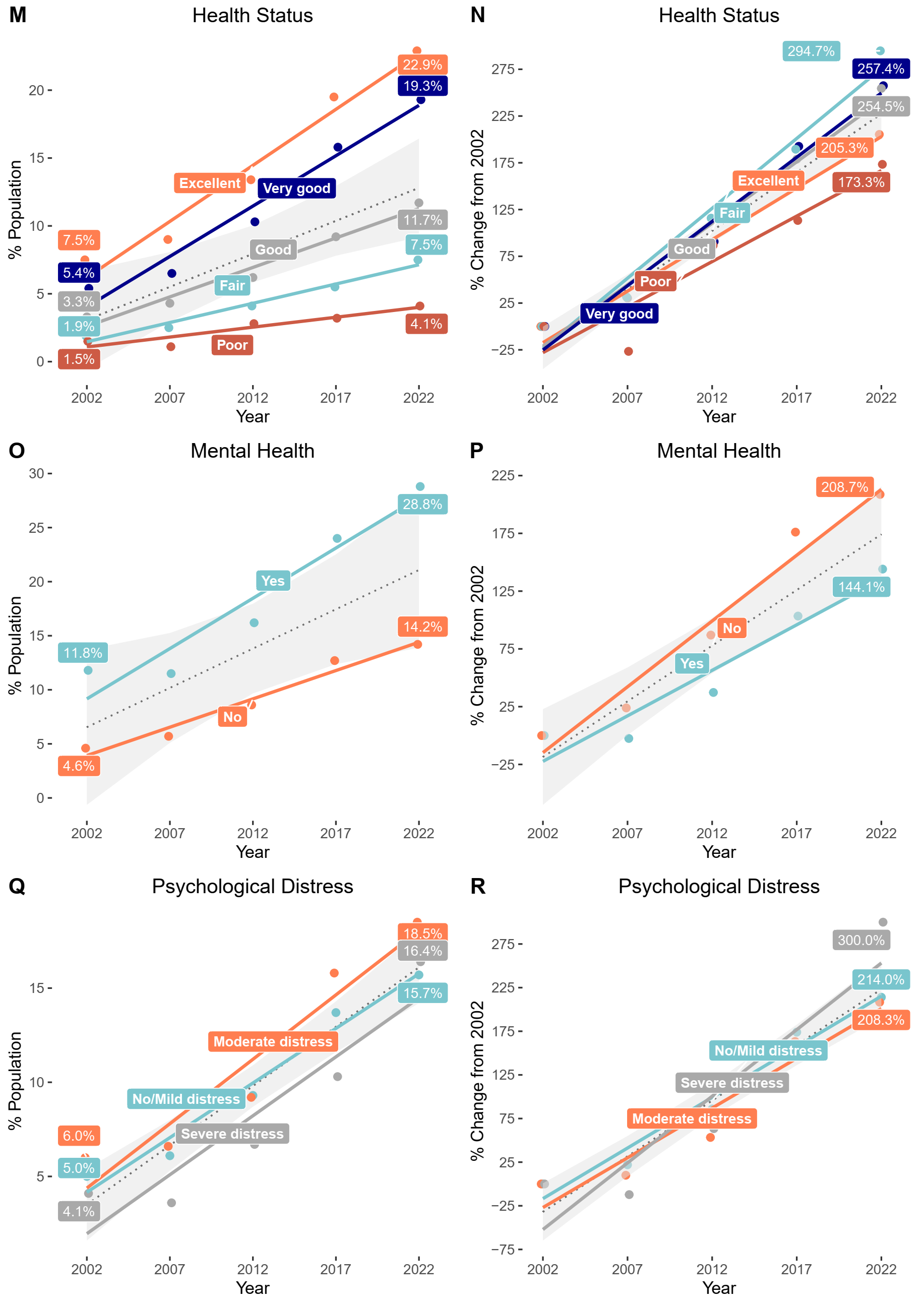


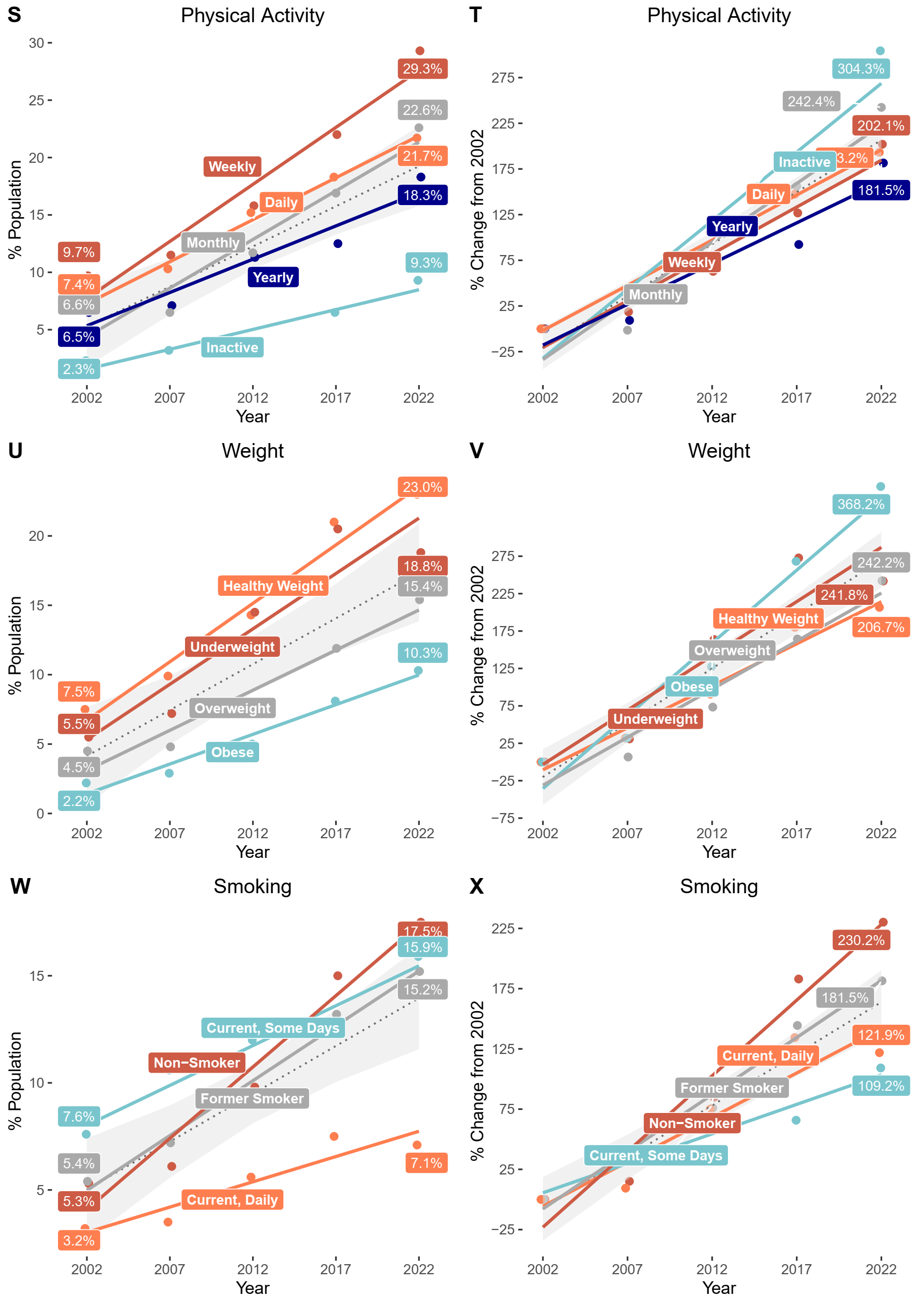


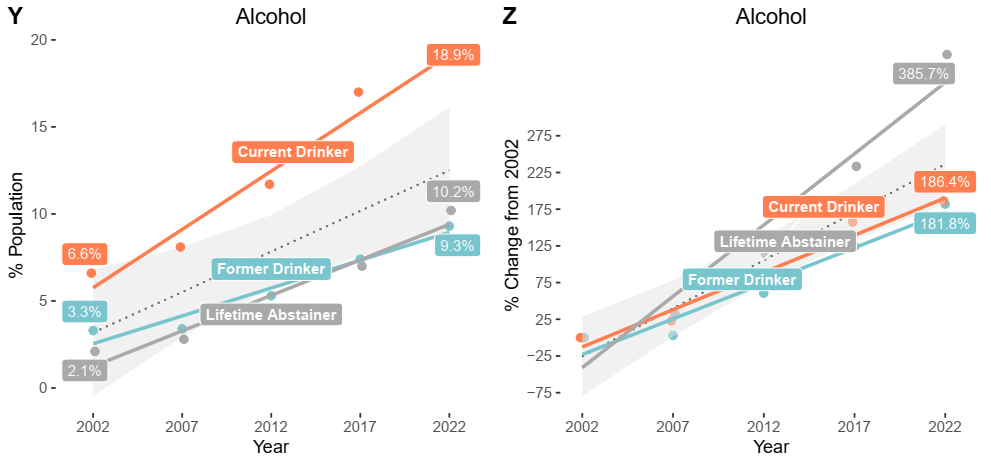


### Figure S2. Changes in the population prevalence and rate of engagement in yoga by different sociodemographic and health subgroups between 2002-2022

Left panel shows changes in the weighted population estimates (dots, percentage labels) and regressed growth rate (solid lines) of yoga by each age (A, B), sex (C, D), race/ethnicity (E, F), relationship status (G, H), educational attainment (I, J), region (K, L), health status (M, N), mental health access (O, P), psychological distress (Q, R), physical activity (S, T), weight status (U, V), smoking status (W, X) and alcohol status (Y, Z) user subgroup compared to the grand average (dashed line with error shading) between 2002 and 2022. Right panel shows change in growth rates, expressed as a percent increase from 2002 at each timepoint (dots, percentage labels; 2007, 2012, 2017, 2022) and regressed rate of change in growth solid lines) for the same user subgroups compared to the grand average (dashed line with error shading). Source: NHIS Data 2002-2022.


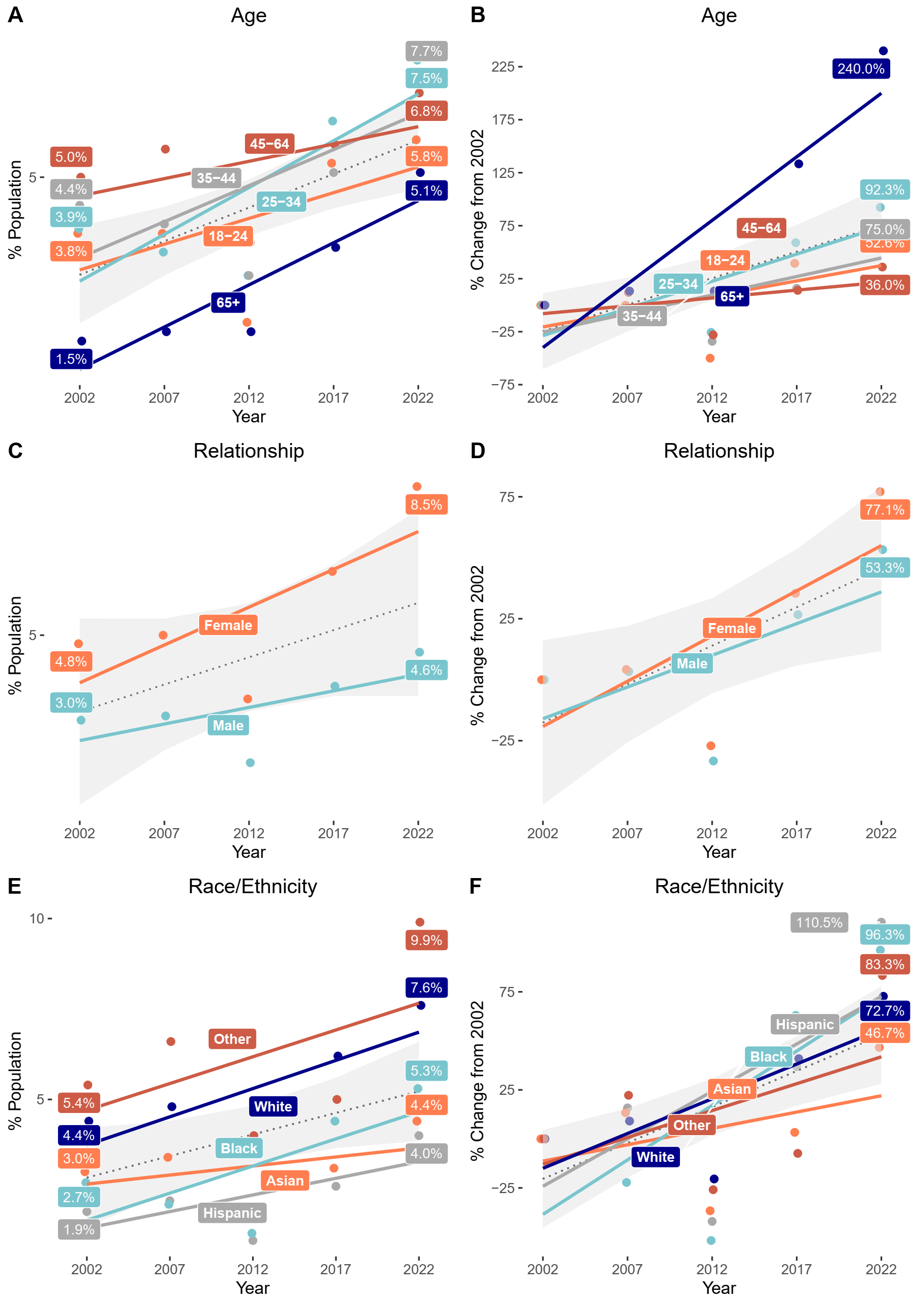


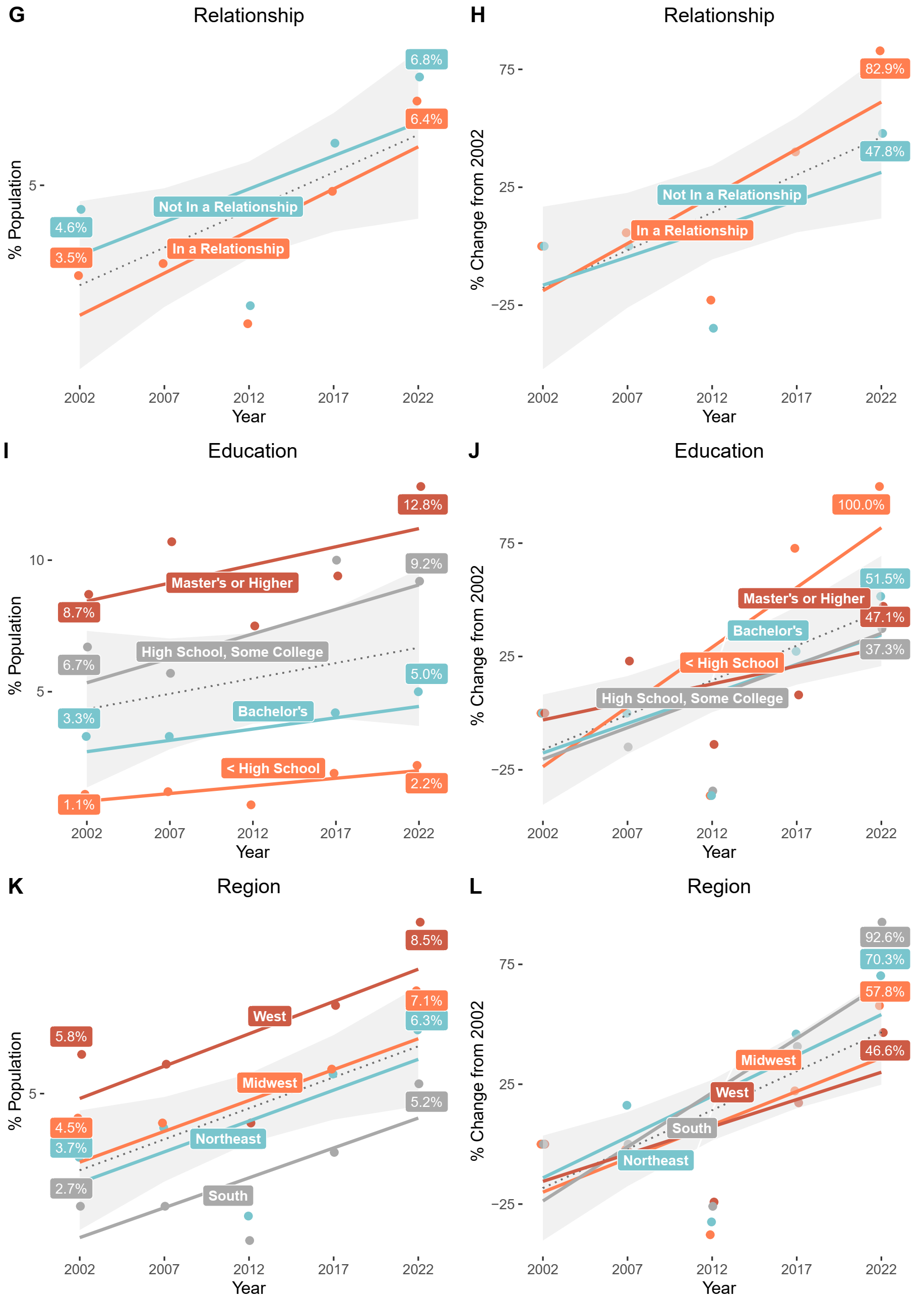


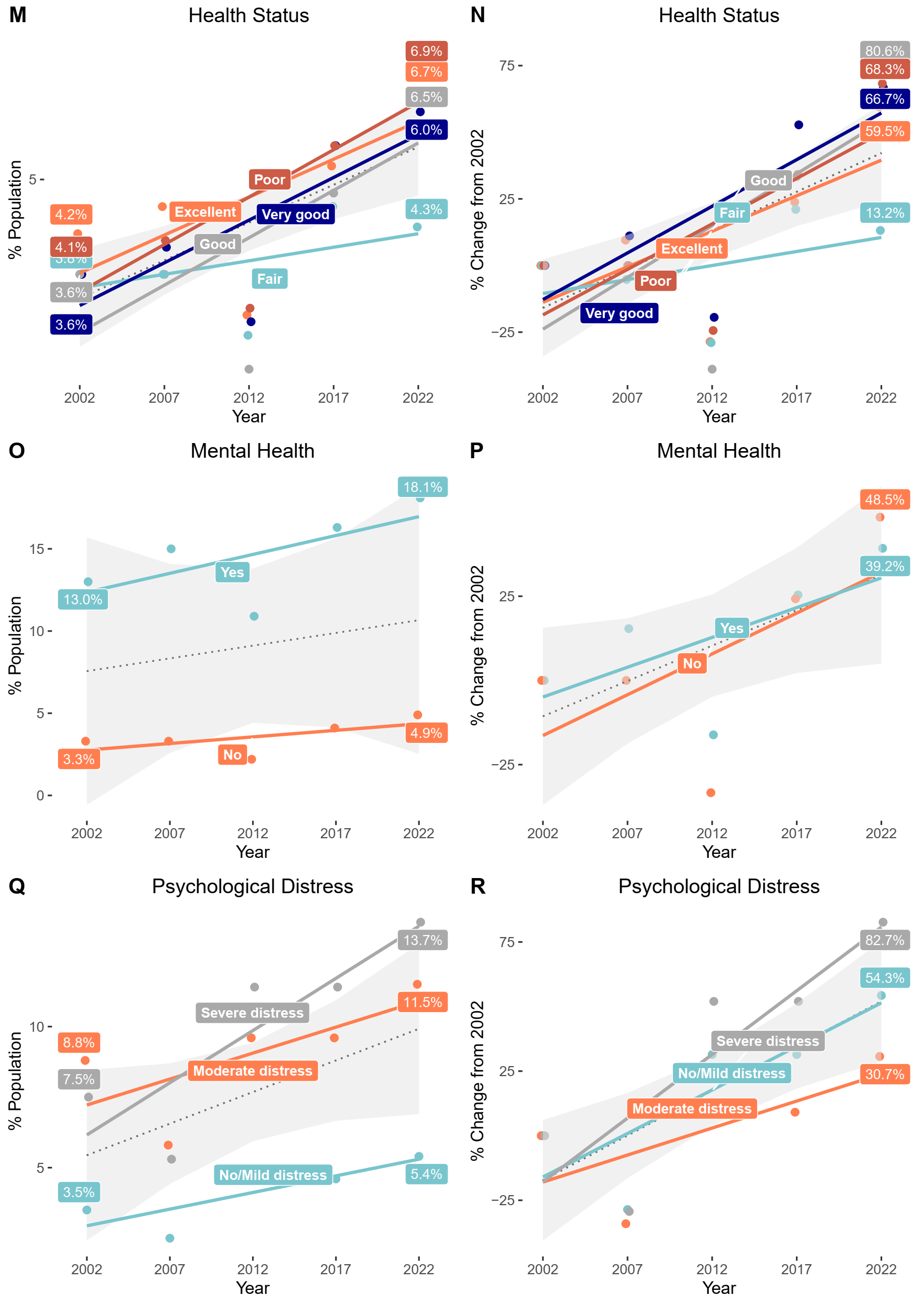


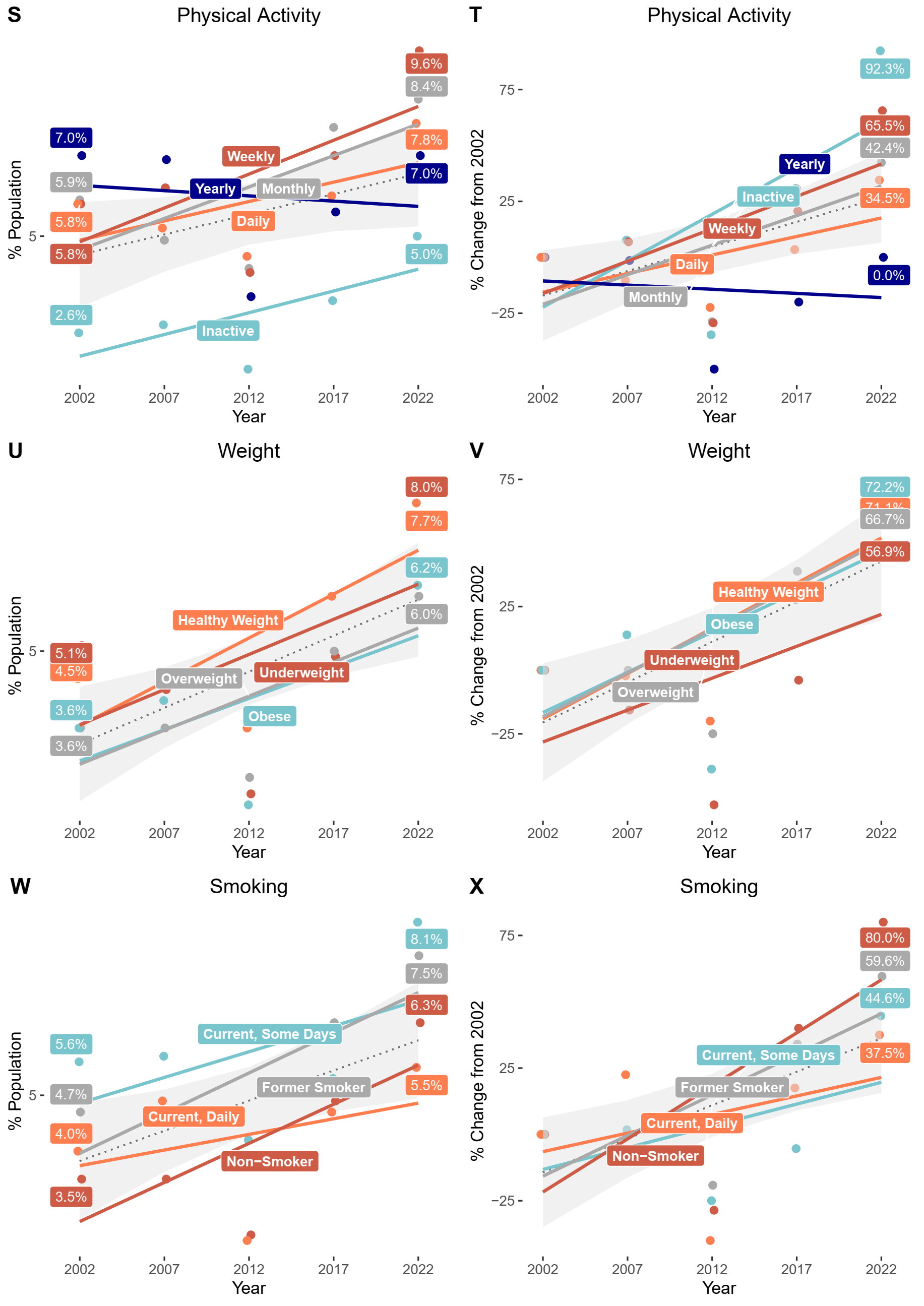


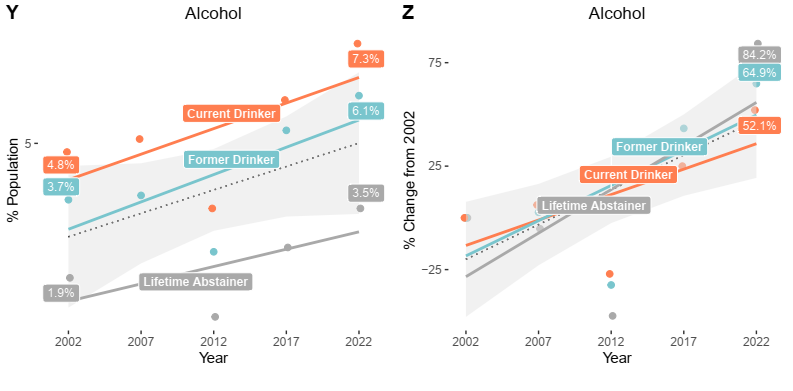


### Figure S3. Changes in the population prevalence and rate of engagement in guided imagery/progressive relaxation by different sociodemographic and health subgroups between 2002-2022

Left panel shows changes in the weighted population estimates (dots, percentage labels) and regressed growth rate (solid lines) of guided imagery/progressive relaxation by each age (A, B), sex (C, D), race/ethnicity (E, F), relationship status (G, H), educational attainment (I, J), region (K, L), health status (M, N), mental health access (O, P), psychological distress (Q, R), physical activity (S, T), weight status (U, V), smoking status (W, X) and alcohol status (Y, Z) user subgroup compared to the grand average (dashed line with error shading) between 2002 and 2022. Right panel shows change in growth rates, expressed as a percent increase from 2002 at each timepoint (dots, percentage labels; 2007, 2012, 2017, 2022) and regressed rate of change in growth solid lines) for the same user subgroups compared to the grand average (dashed line with error shading). Source: NHIS Data 2002-2022.

## Supplemental Tables

### Table S1. Population prevalence and 20-year trend in meditation (Full Data: 2002, 2007, 2012, 2017, 2022)

| **Characteristic** | **2002** |  |  | **2007** |  |  | **2012** |  |  | **2017** |  |  | **2022** |  |  | **20- year change (numeric)** |  | **5-year change (regressed)** |  |  | **1-year change (regressed)** |  |
| --- | --- | --- | --- | --- | --- | --- | --- | --- | --- | --- | --- | --- | --- | --- | --- | --- | --- | --- | --- | --- | --- | --- |
| **Meditation** | % of subgroup | % of total pop | Population  N (000's) | % of subgroup | % of total pop | Population  N (000's) | % of subgroup | % of total pop | Population  N (000's) | % of subgroup | % of total pop | Population  N (000's) | % of subgroup | % of total pop | Population  N (000's) | 20 year change (N, '000) | 20 year change (pop prevalence) | Beta (SE) | stat | p | **%** | **N (‘000)** |
| **Overall (2000 std.)** |  | 7.8% | 15,894 |  | 9.4% | 19,156 |  | 4.1% | 8,284 |  | 14.2% | 28,878 |  | 18.3% | 37,230 | 21,336 | 10.5% | 2.34 (0.1) | 23.19 | < .001 | **0.47** | **9.54** |
| **Overall (2020 std.)** |  |  |  |  |  |  |  |  |  |  |  |  |  | 18.3% | 60,533 |  |  |  |  |  |  |  |
| **Age, years** |  |  |  |  |  |  |  |  |  |  |  |  |  |  |  |  |  | **2.55 (0.11)** | **23.56** | **<.0001*** | **0.51** | **10.40** |
| 18-24 | 7.4% | 1.0% | 2,029 | 8.4% | 1.1% | 2,170 | 3.2% | 0.4% | 841 | 11.6% | 1.4% | 2,842 | 17.3% | 2.0% | 4,159 | 2,130 | 1.0% | **2.28 (0.27)** | **8.51** | **<.0001** | 0.46 | 9.30 |
| 25-34 | 8.8% | 1.6% | 3,207 | 10.4% | 1.8% | 3,770 | 4.8% | 0.8% | 1,714 | 14.6% | 2.6% | 5,244 | 21.2% | 3.7% | 7,590 | 4,384 | 2.2% | **2.43 (0.17)** | **13.98** | **<.0001** | 0.49 | 9.93 |
| 35-44 | 8.3% | 1.8% | 3,618 | 9.4% | 1.8% | 3,578 | 4.0% | 0.7% | 1,358 | 13.5% | 2.2% | 4,512 | 21.1% | 3.6% | 7,325 | 3,707 | 1.8% | **2.58 (0.19)** | **13.68** | **<.0001** | 0.52 | 10.51 |
| 45-64 | 9.0% | 2.8% | 5,711 | 11.2% | 3.8% | 7,703 | 5.0% | 1.7% | 3,498 | 15.9% | 5.3% | 10,877 | 17.1% | 5.7% | 11,531 | 5,819 | 2.9% | **1.87 (0.14)** | **12.99** | **<.0001** | 0.37 | 7.62 |
| 65 or above | 4.1% | 0.7% | 1,330 | 5.9% | 0.9% | 1,936 | 2.4% | 0.4% | 874 | 13.4% | 2.7% | 5,404 | 14.2% | 3.2% | 6,625 | 5,295 | 2.6% | **3.59 (0.21)** | **17.11** | **<.0001** | 0.72 | 14.64 |
| **Gender** |  |  |  |  |  |  |  |  |  |  |  |  |  |  |  |  |  | **2.32 (0.1)** | **22.60** | **<.0001*** | **0.46** | **9.46** |
| Male | 6.6% | 3.1% | 6,367 | 8.2% | 3.9% | 8,028 | 3.2% | 1.5% | 3,147 | 11.8% | 5.7% | 11,568 | 14.4% | 7.2% | 14,633 | 8,265 | 4.1% | **2.15 (0.14)** | **15.36** | **<.0001** | 0.43 | 8.77 |
| Female | 9.0% | 4.7% | 9,526 | 10.7% | 5.5% | 11,128 | 4.9% | 2.5% | 5,137 | 16.6% | 8.5% | 17,310 | 21.1% | 11.1% | 22,597 | 13,071 | 6.4% | **2.49 (0.11)** | **21.76** | **<.0001** | 0.50 | 10.15 |
| **Ethnicity** |  |  |  |  |  |  |  |  |  |  |  |  |  |  |  |  |  | **2.01 (0.16)** | **12.60** | **<.0001*** | **0.40** | **8.19** |
| White | 7.8% | 5.7% | 11,612 | 10.2% | 7.1% | 14,570 | 4.8% | 3.2% | 6,570 | 15.2% | 9.8% | 19,999 | 19.0% | 12.2% | 24,830 | 13,218 | 6.5% | **2.49 (0.12)** | **20.95** | **<.0001** | 0.50 | 10.14 |
| Hispanic | 5.5% | 0.6% | 1,240 | 5.4% | 0.7% | 1,503 | 2.2% | 0.3% | 663 | 10.8% | 1.7% | 3,519 | 12.8% | 2.2% | 4,571 | 3,331 | 1.6% | **2.58 (0.27)** | **9.56** | **<.0001** | 0.52 | 10.51 |
| Black | 8.5% | 0.9% | 1,918 | 9.1% | 1.1% | 2,158 | 2.8% | 0.3% | 650 | 13.8% | 1.6% | 3,357 | 17.8% | 2.1% | 4,245 | 2,328 | 1.1% | **2.16 (0.24)** | **9.00** | **<.0001** | 0.43 | 8.81 |
| Asian | 8.9% | 0.3% | 618 | 11.4% | 0.3% | 653 | 3.1% | 0.2% | 323 | 13.3% | 0.8% | 1,590 | 17.9% | 1.1% | 2,267 | 1,649 | 0.8% | **1.78 (0.41)** | **4.33** | **<.0001** | 0.36 | 7.25 |
| Others | 17.4% | 0.2% | 506 | 13.5% | 0.1% | 271 | 4.7% | 0.0% | 77 | 18.7% | 0.2% | 412 | 22.6% | 0.6% | 1,317 | 811 | 0.4% | 1.04 (0.52) | 2.00 | 0.05 | 0.21 | 4.23 |
| **Relationship Status** |  |  |  |  |  |  |  |  |  |  |  |  |  |  |  |  |  | **2.26 (0.1)** | **22.51** | **<.0001*** | **0.45** | **9.20** |
| In a Relationship | 6.9% | 4.4% | 8,871 | 8.5% | 5.3% | 10,797 | 3.8% | 2.3% | 4,693 | 13.8% | 8.3% | 16,871 | 17.4% | 10.8% | 21,920 | 13,048 | 6.4% | **2.64 (0.13)** | **20.72** | **<.0001** | 0.53 | 10.76 |
| Not in a Relationship | 9.6% | 3.4% | 7,022 | 11.1% | 4.1% | 8,359 | 4.5% | 1.8% | 3,591 | 15.0% | 5.9% | 12,007 | 18.5% | 7.5% | 15,310 | 8,288 | 4.1% | **1.88 (0.13)** | **14.77** | **<.0001** | 0.38 | 7.65 |
| **Education** |  |  |  |  |  |  |  |  |  |  |  |  |  |  |  |  |  | **2.32 (0.12)** | **19.13** | **<.0001*** | **0.46** | **9.45** |
| Less than high school | 3.1% | 0.5% | 1,034 | 3.4% | 0.5% | 1,042 | 1.2% | 0.2% | 350 | 9.2% | 1.1% | 2,202 | 7.5% | 0.8% | 1,647 | 613 | 0.3% | **2.94 (0.34)** | **8.71** | **<.0001** | 0.59 | 11.97 |
| High school, some college | 7.5% | 4.4% | 8,884 | 8.8% | 5.0% | 10,099 | 3.2% | 1.8% | 3,736 | 12.7% | 6.9% | 14,050 | 14.9% | 8.6% | 17,498 | 8,613 | 4.2% | **1.95 (0.13)** | **15.59** | **<.0001** | 0.39 | 7.97 |
| Bachelor | 11.7% | 1.9% | 3,876 | 13.1% | 2.3% | 4,651 | 6.3% | 1.1% | 2,342 | 16.7% | 3.5% | 7,160 | 23.9% | 5.0% | 10,123 | 6,247 | 3.1% | **2.01 (0.17)** | **12.05** | **<.0001** | 0.40 | 8.20 |
| Master or higher | 13.0% | 1.0% | 2,099 | 18.4% | 1.6% | 3,364 | 9.2% | 0.9% | 1,857 | 21.7% | 2.7% | 5,465 | 30.5% | 3.9% | 7,962 | 5,863 | 2.9% | **2.37 (0.21)** | **11.21** | **<.0001** | 0.47 | 9.67 |
| **Region** |  |  |  |  |  |  |  |  |  |  |  |  |  |  |  |  |  | **2.27 (0.1)** | **22.13** | **<.0001*** | **0.45** | **9.26** |
| West | 10.6% | 2.0% | 4,149 | 11.9% | 2.6% | 5,328 | 5.9% | 1.3% | 2,709 | 16.8% | 4.0% | 8,116 | 21.7% | 5.3% | 10,793 | 6,643 | 3.3% | **2.11 (0.21)** | **10.22** | **<.0001** | 0.42 | 8.60 |
| Northeast | 8.2% | 1.6% | 3,188 | 10.3% | 1.7% | 3,536 | 3.8% | 0.7% | 1,409 | 13.2% | 2.4% | 4,842 | 17.8% | 3.2% | 6,453 | 3,265 | 1.6% | **2.04 (0.23)** | **9.04** | **<.0001** | 0.41 | 8.33 |
| Midwest | 8.4% | 2.0% | 4,117 | 10.2% | 2.4% | 4,933 | 4.4% | 1.0% | 2,006 | 14.8% | 3.2% | 6,573 | 17.9% | 3.8% | 7,741 | 3,625 | 1.8% | **2.15 (0.2)** | **10.86** | **<.0001** | 0.43 | 8.76 |
| South | 5.9% | 2.2% | 4,440 | 7.3% | 2.6% | 5,359 | 2.9% | 1.1% | 2,160 | 12.8% | 4.6% | 9,348 | 15.4% | 6.0% | 12,243 | 7,803 | 3.8% | **2.78 (0.17)** | **16.11** | **<.0001** | 0.56 | 11.35 |
| **Health status** |  |  |  |  |  |  |  |  |  |  |  |  |  |  |  |  |  | **2.19 (0.13)** | **17.25** | **<.0001*** | **0.44** | **8.92** |
| Excellent | 8.0% | 2.4% | 4,948 | 9.7% | 2.8% | 5,682 | 4.1% | 1.1% | 2,335 | 14.6% | 4.1% | 8,303 | 18.5% | 4.1% | 8,389 | 3,442 | 1.7% | **2.38 (0.17)** | **14.24** | **<.0001** | 0.48 | 9.70 |
| Very good | 8.1% | 2.6% | 5,207 | 10.2% | 3.2% | 6,506 | 4.7% | 1.5% | 3,039 | 14.1% | 4.6% | 9,291 | 20.3% | 7.2% | 14,607 | 9,400 | 4.6% | **2.51 (0.14)** | **17.38** | **<.0001** | 0.50 | 10.21 |
| Good | 7.6% | 1.9% | 3,891 | 8.5% | 2.2% | 4,479 | 3.6% | 1.0% | 1,953 | 13.6% | 3.6% | 7,338 | 16.1% | 4.9% | 9,890 | 6,000 | 2.9% | **2.22 (0.18)** | **12.61** | **<.0001** | 0.44 | 9.05 |
| Fair | 7.2% | 0.7% | 1,348 | 9.2% | 0.9% | 1,826 | 3.8% | 0.4% | 743 | 15.5% | 1.5% | 3,048 | 15.1% | 1.7% | 3,488 | 2,140 | 1.0% | **2.25 (0.26)** | **8.72** | **<.0001** | 0.45 | 9.18 |
| Poor | 7.9% | 0.2% | 501 | 9.7% | 0.3% | 663 | 3.4% | 0.1% | 214 | 15.9% | 0.4% | 897 | 12.5% | 0.4% | 855 | 354 | 0.2% | **1.58 (0.43)** | **3.71** | **0.001** | 0.32 | 6.44 |
| **Saw Mental Health Professional?** |  |  |  |  |  |  |  |  |  |  |  |  |  |  |  |  |  | **2.02 (0.12)** | **17.42** | **<.0001*** | **0.40** | **8.24** |
| Yes | 20.2% | 1.3% | 2,580 | 26.2% | 1.8% | 3,590 | 13.2% | 1.0% | 2,063 | 29.7% | 2.7% | 5,429 | 37.0% | 4.8% | 9,778 | 7,198 | 3.5% | **1.86 (0.19)** | **9.63** | **<.0001** | 0.37 | 7.57 |
| No | 7.0% | 6.5% | 13,314 | 8.3% | 7.6% | 15,566 | 3.3% | 3.1% | 6,221 | 12.7% | 11.5% | 23,449 | 15.1% | 13.5% | 27,451 | 14,137 | 6.9% | **2.19 (0.11)** | **19.89** | **<.0001** | 0.44 | 8.92 |
| **Psychological Distress (K6)** |  |  |  |  |  |  |  |  |  |  |  |  |  |  |  |  |  | **1.92 (0.15)** | **12.96** | **<.0001*** | **0.38** | **7.82** |
| No/Mild distress | 7.2% | 6.5% | 13,247 | 8.8% | 8.0% | 16,258 | 3.8% | 3.4% | 6,962 | 13.3% | 11.8% | 23,980 | 16.2% | 13.8% | 28,072 | 14,825 | 7.3% | **2.31 (0.11)** | **21.50** | **<.0001** | 0.46 | 9.40 |
| Moderate distress | 14.0% | 0.8% | 1,667 | 17.6% | 1.0% | 1,987 | 7.2% | 0.4% | 865 | 20.9% | 1.6% | 3,244 | 25.0% | 3.1% | 6,309 | 4,643 | 2.3% | **1.63 (0.24)** | **6.68** | **<.0001** | 0.33 | 6.65 |
| Severe distress | 16.2% | 0.5% | 980 | 16.7% | 0.4% | 911 | 7.6% | 0.2% | 458 | 24.2% | 0.8% | 1,654 | 27.6% | 1.4% | 2,849 | 1,868 | 0.9% | **1.82 (0.32)** | **5.68** | **<.0001** | 0.36 | 7.41 |
| **Physical Activity** |  |  |  |  |  |  |  |  |  |  |  |  |  |  |  |  |  | **2.19 (0.2)** | **11.21** | **<.0001*** | **0.44** | **8.93** |
| Inactive | 5.3% | 3.0% | 6,141 | 6.7% | 4.0% | 8,190 | 2.5% | 1.3% | 2,660 | 11.1% | 5.4% | 10,963 | 14.2% | 9.0% | 18,369 | 12,228 | 6.0% | **2.72 (0.14)** | **20.19** | **<.0001** | 0.54 | 11.08 |
| Yearly Exercise | 8.5% | 0.1% | 150 | 13.8% | 0.1% | 266 | 6.0% | 0.1% | 105 | 14.3% | 0.1% | 220 | 23.8% | 0.4% | 717 | 567 | 0.3% | **2.48 (0.79)** | **3.14** | **0.001** | 0.50 | 10.10 |
| Monthly Exercise | 10.1% | 0.3% | 681 | 13.3% | 0.4% | 817 | 6.6% | 0.2% | 429 | 16.3% | 0.6% | 1,283 | 22.4% | 1.4% | 2,865 | 2,184 | 1.1% | **2.13 (0.4)** | **5.35** | **<.0001** | 0.43 | 8.69 |
| Weekly Exercise | 11.1% | 3.5% | 7,060 | 13.9% | 4.2% | 8,594 | 5.7% | 2.1% | 4,198 | 17.8% | 6.9% | 14,032 | 24.6% | 6.8% | 13,920 | 6,860 | 3.4% | **2.2 (0.13)** | **16.96** | **<.0001** | 0.44 | 8.98 |
| Daily Exercise | 12.6% | 0.9% | 1,861 | 12.9% | 0.6% | 1,289 | 6.1% | 0.4% | 892 | 15.9% | 1.2% | 2,380 | 20.6% | 0.7% | 1,358 | -503 | -0.2% | **1.42 (0.32)** | **4.38** | **<.0001** | 0.28 | 5.79 |
| **Weight status** |  |  |  |  |  |  |  |  |  |  |  |  |  |  |  |  |  | **2.39 (0.17)** | **14.30** | **<.0001*** | **0.48** | **9.76** |
| Healthy Weight | 8.7% | 3.3% | 6,659 | 10.1% | 3.5% | 7,179 | 4.8% | 1.6% | 3,315 | 16.0% | 5.1% | 10,400 | 20.4% | 6.5% | 13,290 | 6,632 | 3.3% | **2.51 (0.14)** | **17.57** | **<.0001** | 0.50 | 10.22 |
| Underweight | 9.0% | 0.2% | 351 | 7.5% | 0.1% | 265 | 2.6% | 0.0% | 91 | 14.9% | 0.3% | 537 | 18.8% | 0.3% | 657 | 305 | 0.1% | **2.47 (0.57)** | **4.36** | **<.0001** | 0.49 | 10.06 |
| Overweight | 7.1% | 2.4% | 4,835 | 9.1% | 3.0% | 6,215 | 4.0% | 1.3% | 2,700 | 13.4% | 4.4% | 9,053 | 17.1% | 5.9% | 12,004 | 7,169 | 3.5% | **2.41 (0.15)** | **16.12** | **<.0001** | 0.48 | 9.84 |
| Obese | 7.4% | 2.0% | 4,048 | 9.4% | 2.7% | 5,497 | 3.5% | 1.1% | 2,178 | 13.4% | 4.4% | 8,888 | 16.3% | 5.5% | 11,278 | 7,230 | 3.5% | **2.19 (0.16)** | **13.98** | **<.0001** | 0.44 | 8.91 |
| **Smoking Status** |  |  |  |  |  |  |  |  |  |  |  |  |  |  |  |  |  | **1.88 (0.13)** | **14.55** | **<.0001*** | **0.38** | **7.66** |
| Non-Smoker | 7.0% | 3.8% | 7,694 | 8.3% | 4.8% | 9,776 | 3.7% | 2.2% | 4,425 | 14.1% | 8.9% | 18,087 | 17.8% | 12.0% | 24,494 | 16,799 | 8.2% | **2.7 (0.13)** | **21.59** | **<.0001** | 0.54 | 11.02 |
| Former Smoker | 8.6% | 1.9% | 3,970 | 11.4% | 2.4% | 4,950 | 5.1% | 1.1% | 2,271 | 15.7% | 3.5% | 7,149 | 19.3% | 4.4% | 9,047 | 5,077 | 2.5% | **2.24 (0.15)** | **14.51** | **<.0001** | 0.45 | 9.12 |
| Current, Some Days | 12.1% | 0.5% | 996 | 13.8% | 0.6% | 1,215 | 7.2% | 0.3% | 561 | 14.7% | 0.5% | 1,036 | 20.6% | 0.6% | 1,155 | 159 | 0.1% | **1.35 (0.36)** | **3.75** | **0.001** | 0.27 | 5.48 |
| Current, Daily | 8.7% | 1.6% | 3,233 | 10.4% | 1.6% | 3,215 | 3.6% | 0.5% | 1,027 | 12.3% | 1.3% | 2,606 | 13.7% | 1.2% | 2,534 | -700 | -0.3% | **1.23 (0.22)** | **5.59** | **<.0001** | 0.25 | 5.03 |
| **Alcohol Status** |  |  |  |  |  |  |  |  |  |  |  |  |  |  |  |  |  | **2.39 (0.13)** | **18.47** | **<.0001*** | **0.48** | **9.74** |
| Lifetime Abstainer | 4.5% | 1.0% | 1,989 | 4.4% | 1.0% | 2,079 | 1.8% | 0.4% | 763 | 11.3% | 2.2% | 4,486 | 11.7% | 1.6% | 3,252 | 1,263 | 0.6% | **3.07 (0.27)** | **11.24** | **<.0001** | 0.61 | 12.51 |
| Former Drinker | 8.9% | 1.3% | 2,718 | 10.4% | 1.5% | 3,045 | 3.8% | 0.5% | 1,102 | 15.3% | 2.0% | 4,151 | 17.6% | 3.1% | 6,327 | 3,609 | 1.8% | **2.01 (0.2)** | **10.08** | **<.0001** | 0.40 | 8.18 |
| Current Drinker | 8.8% | 5.5% | 11,187 | 11.5% | 6.9% | 14,032 | 4.9% | 3.1% | 6,420 | 15.0% | 9.9% | 20,241 | 19.1% | 13.6% | 27,651 | 16,464 | 8.1% | **2.09 (0.11)** | **18.77** | **<.0001** | 0.42 | 8.52 |

Population weighted prevalence estimates were calculated for each timepoint (2002, 2007, 2012, 2017, 2022) to represent the number (N, in thousands) of individuals who used the practice in the past 12 months as well as percentage estimates at both a subgroup level (i.e., with all individuals in that group in the population as the denominator) and at a whole population level (i.e., % of individuals in the whole population as the denominator). Changes over time are represented as: (a) 20-year numeric changes (i.e., 2022 prevalence minus 2002 prevalence); (b) 5-year regressed changes (β), and (c) 1-year regressed changes (i.e., β/5). For regressions at the group level (significance set at false discovery rate (FDR) adjusted *p*<0.05) and subgroup level, significance is shown in bold, FDR adjusted significance is denoted by (*) and non-significance by (^ns^). Text in green denotes the largest, and red denotes the smallest value in the group. We used the US population standard from the year 2000^24^ in all analyses to control for the effects of population growth. However, for 2022, a population estimate using the most recent population standard (2020) was applied to estimate the total number of individuals using the practice in 2022. Source: NHIS Data 2002-2022.

### Table S2. Population prevalence and 20-year trend in yoga (Full Data: 2002, 2007, 2012, 2017, 2022)

| **Characteristic** | **2002** |  |  | **2007** |  |  | **2012** |  |  | **2017** |  |  | **2022** |  |  | **20- year change (numeric)** |  | **5-year change (regressed)** |  |  | **1-year change (regressed)** |  |
| --- | --- | --- | --- | --- | --- | --- | --- | --- | --- | --- | --- | --- | --- | --- | --- | --- | --- | --- | --- | --- | --- | --- |
| **Yoga** | % of population (or sub pop) | % of total pop | Population  N (000's) | % of population (or sub pop) | % of total pop | Population  N (000's) | % of population (or sub pop) | % of total pop | Population  N (000's) | % of population (or sub pop) | % of total pop | Population  N (000's) | % of population (or sub pop) | % of total pop | Population  N (000's) | 20 year change (N, '000) | 20 year change (pop prevalence) | Beta (SE) | stat | p | **%** | **N (‘000)** |
| **Overall (2000 std.)** | 5.0% | 5.0% | 10,192 | 6.1% | 6.1% | 12,436 | 9.5% | 9.5% | 19,313 | 14.1% | 14.1% | 28,821 | 16.8% | 16.8% | 34,307 | 24,115 | 11.8% | 3.46 (0.11) | 31.65 | <.0001 | **0.69** | **14.11** |
| **Overall (2020 std.)** |  |  |  |  |  |  |  |  |  |  |  |  |  | 16.8% | 55,781 |  |  |  |  |  |  |  |
| **Age, years** |  |  |  |  |  |  |  |  |  |  |  |  |  |  |  |  |  | **3.89 (0.12)** | **32.98** | **<.0001** | **0.78** | **15.84** |
| 18-24 | 5.4% | 0.7% | 1,478 | 8.1% | 1.0% | 2,126 | 11.4% | 1.5% | 3,066 | 18.1% | 2.2% | 4,575 | 18.8% | 2.3% | 4,631 | 3,153 | 1.5% | **3.71 (0.28)** | **13.09** | **<.0001** | 0.74 | 15.11 |
| 25-34 | 7.4% | 1.3% | 2,703 | 9.1% | 1.7% | 3,364 | 14.3% | 2.6% | 5,252 | 20.2% | 3.7% | 7,560 | 23.9% | 4.3% | 8,770 | 6,066 | 3.0% | **3.65 (0.19)** | **19.29** | **<.0001** | 0.73 | 14.88 |
| 35-44 | 5.6% | 1.2% | 2,454 | 6.5% | 1.2% | 2,528 | 11.2% | 1.9% | 3,952 | 15.2% | 2.6% | 5,260 | 20.4% | 3.6% | 7,263 | 4,808 | 2.4% | **3.85 (0.2)** | **18.90** | **<.0001** | 0.77 | 15.70 |
| 45-64 | 5.0% | 1.5% | 3,135 | 5.4% | 1.8% | 3,755 | 7.8% | 2.8% | 5,650 | 12.2% | 4.2% | 8,628 | 14.1% | 4.8% | 9,768 | 6,634 | 3.3% | **3.19 (0.18)** | **17.68** | **<.0001** | 0.64 | 12.99 |
| 65 or above | 1.3% | 0.2% | 422 | 2.0% | 0.3% | 663 | 3.8% | 0.7% | 1,394 | 6.7% | 1.4% | 2,798 | 8.1% | 1.9% | 3,875 | 3,453 | 1.7% | **5.04 (0.34)** | **15.00** | **<.0001** | 1.01 | 20.53 |
| **Gender** |  |  |  |  |  |  |  |  |  |  |  |  |  |  |  |  |  | **3.67 (0.12)** | **30.05** | **<.0001** | **0.73** | **14.98** |
| Male | 2.5% | 1.2% | 2,444 | 2.9% | 1.4% | 2,887 | 5.0% | 2.5% | 5,083 | 8.3% | 4.1% | 8,419 | 9.8% | 5.0% | 10,228 | 7,784 | 3.8% | **3.98 (0.2)** | **20.30** | **<.0001** | 0.80 | 16.21 |
| Female | 7.3% | 3.8% | 7,748 | 9.0% | 4.7% | 9,549 | 13.1% | 7.0% | 14,231 | 18.8% | 10.0% | 20,403 | 21.9% | 11.8% | 24,079 | 16,331 | 8.0% | **3.37 (0.12)** | **27.20** | **<.0001** | 0.67 | 13.75 |
| **Ethnicity** |  |  |  |  |  |  |  |  |  |  |  |  |  |  |  |  |  | **3.29 (0.18)** | **18.61** | **<.0001** | **0.66** | **13.40** |
| White | 5.5% | 4.0% | 8,141 | 6.9% | 4.9% | 10,034 | 10.4% | 7.2% | 14,627 | 15.6% | 10.4% | 21,270 | 17.5% | 11.5% | 23,388 | 15,247 | 7.5% | **3.49 (0.12)** | **28.33** | **<.0001** | 0.70 | 14.22 |
| Hispanic | 2.8% | 0.3% | 630 | 2.9% | 0.4% | 804 | 5.6% | 0.9% | 1,774 | 8.4% | 1.4% | 2,845 | 10.8% | 2.0% | 3,978 | 3,348 | 1.6% | **4.00 (0.3)** | **13.48** | **<.0001** | 0.80 | 16.30 |
| Black | 2.8% | 0.3% | 623 | 3.2% | 0.4% | 774 | 5.7% | 0.7% | 1,396 | 9.4% | 1.2% | 2,376 | 12.4% | 1.5% | 3,034 | 2,411 | 1.2% | **4.35 (0.31)** | **14.07** | **<.0001** | 0.87 | 17.73 |
| Asian | 7.3% | 0.2% | 508 | 11.7% | 0.3% | 680 | 12.2% | 0.6% | 1,322 | 16.9% | 1.0% | 2,106 | 22.2% | 1.4% | 2,878 | 2,370 | 1.2% | **2.99 (0.38)** | **7.96** | **<.0001** | 0.60 | 12.18 |
| Others | 9.9% | 0.1% | 290 | 7.1% | 0.1% | 145 | 11.4% | 0.1% | 194 | 9.8% | 0.1% | 225 | 17.2% | 0.5% | 1,029 | 739 | 0.4% | **1.61 (0.65)** | **2.47** | **0.009** | 0.32 | 6.57 |
| **Relationship Status** |  |  |  |  |  |  |  |  |  |  |  |  |  |  |  |  |  | **3.41 (0.11)** | **31.19** | **<.0001** | **0.68** | **13.90** |
| In a Relationship | 4.8% | 3.1% | 6,222 | 5.9% | 3.7% | 7,608 | 9.1% | 5.6% | 11,473 | 13.8% | 8.6% | 17,461 | 16.3% | 10.4% | 21,151 | 14,929 | 7.3% | **3.63 (0.14)** | **26.19** | **<.0001** | 0.73 | 14.80 |
| Not in a Relationship | 5.4% | 1.9% | 3,970 | 6.3% | 2.4% | 4,828 | 9.5% | 3.8% | 7,840 | 13.7% | 5.6% | 11,360 | 15.5% | 6.5% | 13,156 | 9,186 | 4.5% | **3.19 (0.15)** | **21.23** | **<.0001** | 0.64 | 12.99 |
| **Education** |  |  |  |  |  |  |  |  |  |  |  |  |  |  |  |  |  | **3.54 (0.17)** | **20.35** | **<.0001** | **0.71** | **14.44** |
| Less than high school | 0.7% | 0.1% | 242 | 1.0% | 0.2% | 308 | 2.0% | 0.3% | 597 | 3.5% | 0.4% | 866 | 3.9% | 0.4% | 887 | 645 | 0.3% | **4.75 (0.61)** | **7.74** | **<.0001** | 0.95 | 19.36 |
| High school, some college | 4.0% | 2.3% | 4,774 | 4.8% | 2.7% | 5,597 | 7.2% | 4.2% | 8,595 | 10.1% | 5.7% | 11,538 | 11.7% | 6.9% | 14,150 | 9,376 | 4.6% | **3.10 (0.15)** | **20.71** | **<.0001** | 0.62 | 12.65 |
| Bachelor | 10.4% | 1.7% | 3,437 | 10.6% | 1.9% | 3,816 | 15.8% | 3.0% | 6,075 | 22.0% | 4.8% | 9,760 | 25.6% | 5.5% | 11,110 | 7,674 | 3.8% | **3.04 (0.17)** | **18.29** | **<.0001** | 0.61 | 12.40 |
| Master or higher | 10.7% | 0.9% | 1,740 | 14.6% | 1.3% | 2,716 | 19.4% | 2.0% | 4,046 | 25.5% | 3.3% | 6,658 | 30.5% | 4.0% | 8,160 | 6,420 | 3.1% | **3.28 (0.22)** | **15.09** | **<.0001** | 0.66 | 13.36 |
| **Region** |  |  |  |  |  |  |  |  |  |  |  |  |  |  |  |  |  | **3.44 (0.11)** | **31.65** | **<.0001** | **0.69** | **14.02** |
| West | 6.9% | 1.3% | 2,688 | 8.0% | 1.8% | 3,632 | 12.7% | 3.0% | 6,036 | 17.2% | 4.2% | 8,632 | 18.2% | 4.6% | 9,278 | 6,591 | 3.2% | **3.09 (0.23)** | **13.34** | **<.0001** | 0.62 | 12.58 |
| Northeast | 6.0% | 1.1% | 2,333 | 7.4% | 1.3% | 2,559 | 9.0% | 1.7% | 3,428 | 14.6% | 2.7% | 5,557 | 17.9% | 3.3% | 6,668 | 4,335 | 2.1% | **3.22 (0.21)** | **15.51** | **<.0001** | 0.64 | 13.12 |
| Midwest | 5.0% | 1.2% | 2,422 | 6.1% | 1.5% | 2,994 | 9.6% | 2.2% | 4,579 | 14.5% | 3.3% | 6,697 | 17.1% | 3.7% | 7,597 | 5,175 | 2.5% | **3.71 (0.22)** | **16.53** | **<.0001** | 0.74 | 15.12 |
| South | 3.6% | 1.3% | 2,750 | 4.3% | 1.6% | 3,251 | 6.9% | 2.6% | 5,271 | 10.5% | 3.9% | 7,935 | 13.2% | 5.3% | 10,763 | 8,014 | 3.9% | **3.74 (0.2)** | **18.55** | **<.0001** | 0.75 | 15.26 |
| **Health status** |  |  |  |  |  |  |  |  |  |  |  |  |  |  |  |  |  | **3.55 (0.21)** | **17.18** | **<.0001** | **0.71** | **14.47** |
| Excellent | 7.5% | 2.3% | 4,607 | 9.0% | 2.6% | 5,356 | 13.4% | 3.9% | 7,900 | 19.5% | 5.6% | 11,512 | 22.9% | 5.2% | 10,635 | 6,028 | 3.0% | **3.49 (0.16)** | **21.73** | **<.0001** | 0.70 | 14.24 |
| Very good | 5.4% | 1.7% | 3,464 | 6.5% | 2.1% | 4,202 | 10.3% | 3.4% | 6,934 | 15.8% | 5.3% | 10,830 | 19.3% | 7.0% | 14,252 | 10,789 | 5.3% | **3.88 (0.17)** | **23.02** | **<.0001** | 0.78 | 15.82 |
| Good | 3.3% | 0.8% | 1,669 | 4.3% | 1.1% | 2,306 | 6.2% | 1.7% | 3,473 | 9.2% | 2.5% | 5,174 | 11.7% | 3.6% | 7,350 | 5,681 | 2.8% | **3.54 (0.23)** | **15.47** | **<.0001** | 0.71 | 14.43 |
| Fair | 1.9% | 0.2% | 357 | 2.5% | 0.2% | 499 | 4.1% | 0.4% | 825 | 5.5% | 0.5% | 1,118 | 7.5% | 0.9% | 1,782 | 1,425 | 0.7% | **3.68 (0.42)** | **8.87** | **<.0001** | 0.74 | 15.01 |
| Poor | 1.5% | 0.0% | 96 | 1.1% | 0.0% | 74 | 2.8% | 0.1% | 182 | 3.2% | 0.1% | 186 | 4.1% | 0.1% | 288 | 191 | 0.1% | **3.15 (0.82)** | **3.85** | **0.001** | 0.63 | 12.85 |
| **Saw Mental Health Professional?** |  |  |  |  |  |  |  |  |  |  |  |  |  |  |  |  |  | **3.22 (0.15)** | **22.00** | **<.0001** | **0.64** | **13.14** |
| Yes | 11.8% | 0.7% | 1,509 | 11.5% | 0.8% | 1,596 | 16.2% | 1.3% | 2,630 | 24.0% | 2.2% | 4,551 | 28.8% | 3.8% | 7,796 | 6,287 | 3.1% | **3.09 (0.25)** | **12.27** | **<.0001** | 0.62 | 12.61 |
| No | 4.6% | 4.3% | 8,683 | 5.7% | 5.3% | 10,840 | 8.6% | 8.2% | 16,683 | 12.7% | 11.9% | 24,270 | 14.2% | 13.0% | 26,510 | 17,827 | 8.7% | **3.35 (0.11)** | **29.53** | **<.0001** | 0.67 | 13.66 |
| **Psychological Distress (K6)** |  |  |  |  |  |  |  |  |  |  |  |  |  |  |  |  |  | **3.71 (0.22)** | **16.61** | **<.0001** | **0.74** | **15.14** |
| No/Mild distress | 5.0% | 4.5% | 9,235 | 6.1% | 5.6% | 11,489 | 9.3% | 8.7% | 17,753 | 13.7% | 12.5% | 25,536 | 15.7% | 13.6% | 27,776 | 18,541 | 9.1% | **3.41 (0.11)** | **30.38** | **<.0001** | 0.68 | 13.91 |
| Moderate distress | 6.0% | 0.3% | 713 | 6.6% | 0.4% | 749 | 9.2% | 0.6% | 1,139 | 15.8% | 1.3% | 2,555 | 18.5% | 2.4% | 4,792 | 4,079 | 2.0% | **3.53 (0.31)** | **11.49** | **<.0001** | 0.71 | 14.38 |
| Severe distress | 4.1% | 0.1% | 245 | 3.6% | 0.1% | 198 | 6.7% | 0.2% | 422 | 10.3% | 0.4% | 730 | 16.4% | 0.9% | 1,739 | 1,494 | 0.7% | **4.20 (0.57)** | **7.37** | **<.0001** | 0.84 | 17.14 |
| **Physical Activity** |  |  |  |  |  |  |  |  |  |  |  |  |  |  |  |  |  | **3.45 (0.22)** | **15.73** | **<.0001** | **0.69** | **14.06** |
| Inactive | 2.3% | 1.3% | 2,578 | 3.2% | 1.9% | 3,853 | 3.8% | 2.0% | 4,145 | 6.5% | 3.2% | 6,613 | 9.3% | 6.0% | 12,308 | 9,730 | 4.8% | **3.72 (0.17)** | **21.57** | **<.0001** | 0.74 | 15.15 |
| Yearly Exercise | 6.5% | 0.1% | 113 | 7.1% | 0.1% | 137 | 11.3% | 0.1% | 204 | 12.5% | 0.1% | 196 | 18.3% | 0.3% | 566 | 453 | 0.2% | **2.95 (0.86)** | **3.42** | **0.001** | 0.59 | 12.04 |
| Monthly Exercise | 6.6% | 0.2% | 434 | 6.5% | 0.2% | 400 | 11.7% | 0.4% | 777 | 16.9% | 0.7% | 1,370 | 22.6% | 1.4% | 2,956 | 2,521 | 1.2% | **3.92 (0.46)** | **8.53** | **<.0001** | 0.78 | 15.99 |
| Weekly Exercise | 9.7% | 2.9% | 5,995 | 11.5% | 3.4% | 7,029 | 15.8% | 5.8% | 11,910 | 22.0% | 8.7% | 17,835 | 29.3% | 8.3% | 17,013 | 11,018 | 5.4% | **3.49 (0.14)** | **25.36** | **<.0001** | 0.70 | 14.24 |
| Daily Exercise | 7.4% | 0.5% | 1,072 | 10.3% | 0.5% | 1,017 | 15.2% | 1.1% | 2,278 | 18.3% | 1.4% | 2,806 | 21.7% | 0.7% | 1,465 | 393 | 0.2% | **3.16 (0.33)** | **9.64** | **<.0001** | 0.63 | 12.86 |
| **Weight status** |  |  |  |  |  |  |  |  |  |  |  |  |  |  |  |  |  | **3.86 (0.18)** | **20.97** | **<.0001** | **0.77** | **15.75** |
| Healthy Weight | 7.5% | 2.8% | 5,740 | 9.9% | 3.5% | 7,132 | 14.3% | 5.0% | 10,103 | 21.0% | 6.9% | 14,089 | 23.0% | 7.5% | 15,335 | 9,596 | 4.7% | **3.48 (0.15)** | **23.29** | **<.0001** | 0.70 | 14.19 |
| Underweight | 5.5% | 0.1% | 213 | 7.2% | 0.1% | 260 | 14.5% | 0.3% | 514 | 20.5% | 0.4% | 767 | 18.8% | 0.3% | 671 | 458 | 0.2% | **3.96 (0.65)** | **6.08** | **<.0001** | 0.79 | 16.13 |
| Overweight | 4.5% | 1.5% | 3,025 | 4.8% | 1.6% | 3,298 | 7.8% | 2.7% | 5,505 | 11.9% | 4.1% | 8,398 | 15.4% | 5.4% | 11,030 | 8,005 | 3.9% | **3.72 (0.16)** | **23.25** | **<.0001** | 0.74 | 15.15 |
| Obese | 2.2% | 0.6% | 1,215 | 2.9% | 0.9% | 1,747 | 5.0% | 1.6% | 3,191 | 8.1% | 2.7% | 5,567 | 10.3% | 3.6% | 7,271 | 6,056 | 3.0% | **4.30 (0.22)** | **19.94** | **<.0001** | 0.86 | 17.53 |
| **Smoking Status** |  |  |  |  |  |  |  |  |  |  |  |  |  |  |  |  |  | **2.74 (0.16)** | **17.54** | **<.0001** | **0.55** | **11.18** |
| Non-Smoker | 5.3% | 2.9% | 5,842 | 6.1% | 3.6% | 7,253 | 9.8% | 6.0% | 12,284 | 15.0% | 9.8% | 20,027 | 17.5% | 12.1% | 24,727 | 18,885 | 9.3% | **3.67 (0.13)** | **28.11** | **<.0001** | 0.73 | 14.94 |
| Former Smoker | 5.4% | 1.2% | 2,513 | 7.2% | 1.5% | 3,147 | 9.5% | 2.2% | 4,409 | 13.2% | 3.1% | 6,230 | 15.2% | 3.6% | 7,312 | 4,799 | 2.4% | **2.96 (0.19)** | **16.01** | **<.0001** | 0.59 | 12.06 |
| Current, Some Days | 7.6% | 0.3% | 624 | 10.6% | 0.5% | 946 | 12.0% | 0.5% | 969 | 12.6% | 0.5% | 924 | 15.9% | 0.4% | 915 | 290 | 0.1% | **1.87 (0.43)** | **4.38** | **<.0001** | 0.37 | 7.63 |
| Current, Daily | 3.2% | 0.6% | 1,213 | 3.5% | 0.5% | 1,090 | 5.6% | 0.8% | 1,651 | 7.5% | 0.8% | 1,639 | 7.1% | 0.7% | 1,353 | 140 | 0.1% | **2.47 (0.31)** | **7.97** | **<.0001** | 0.49 | 10.08 |
| **Alcohol Status** |  |  |  |  |  |  |  |  |  |  |  |  |  |  |  |  |  | **3.53 (0.16)** | **22.15** | **<.0001** | **0.71** | **14.40** |
| Lifetime Abstainer | 2.1% | 0.4% | 916 | 2.8% | 0.7% | 1,338 | 4.5% | 1.0% | 1,969 | 7.0% | 1.4% | 2,881 | 10.2% | 1.4% | 2,917 | 2,001 | 1.0% | **4.32 (0.33)** | **13.06** | **<.0001** | 0.86 | 17.60 |
| Former Drinker | 3.3% | 0.5% | 990 | 3.4% | 0.5% | 1,021 | 5.3% | 0.8% | 1,611 | 7.4% | 1.0% | 2,075 | 9.3% | 1.7% | 3,417 | 2,427 | 1.2% | **3.04 (0.3)** | **10.28** | **<.0001** | 0.61 | 12.40 |
| Current Drinker | 6.6% | 4.1% | 8,286 | 8.1% | 4.9% | 10,077 | 11.7% | 7.7% | 15,733 | 17.0% | 11.7% | 23,865 | 18.9% | 13.7% | 27,973 | 19,687 | 9.7% | **3.24 (0.12)** | **27.85** | **<.0001** | 0.65 | 13.22 |

Population weighted prevalence estimates were calculated for each timepoint (2002, 2007, 2012, 2017, 2022) to represent the number (N, in thousands) of individuals who used the practice in the past 12 months as well as percentage estimates at both a subgroup level (i.e., with all individuals in that group in the population as the denominator) and at a whole population level (i.e., % of individuals in the whole population as the denominator). Changes over time are represented as: (a) 20-year numeric changes (i.e., 2022 prevalence minus 2002 prevalence); (b) 5-year regressed changes (β), and (c) 1-year regressed changes (i.e., β/5). For regressions at the group level (significance set at false discovery rate (FDR) adjusted *p*<0.05) and subgroup level, significance is shown in bold, FDR adjusted significance is denoted by (*) and non-significance by (^ns^). Text in green denotes the largest, and red denotes the smallest value in the group. We used the US population standard from the year 2000^24^ in all analyses to control for the effects of population growth. However, for 2022, a population estimate using the most recent population standard (2020) was applied to estimate the total number of individuals using the practice in 2022. Source: NHIS Data 2002-2022.

### Table S3. Population prevalence and 20-year trend in GIPR (Full Data: 2002, 2007, 2012, 2017, 2022)

| **Characteristic** | **2002** |  |  | **2007** |  |  | **2012** |  |  | **2017** |  |  | **2022** |  |  | **20- year change (numeric)** |  | **5-year change (regressed)** |  |  | **1-year change (regressed)** |  |
| --- | --- | --- | --- | --- | --- | --- | --- | --- | --- | --- | --- | --- | --- | --- | --- | --- | --- | --- | --- | --- | --- | --- |
| **Guided Imagery / Progressive Relaxation** | % of population (or sub pop) | % of total pop | Population  N (000's) | % of population (or sub pop) | % of total pop | Population  N (000's) | % of population (or sub pop) | % of total pop | Population  N (000's) | % of population (or sub pop) | % of total pop | Population  N (000's) | % of population (or sub pop) | % of total pop | Population  N (000's) | 20 year change (N, '000) | 20 year change (pop prevalence) | Beta (SE) | stat | p | **%** | **N (‘000)** |
| **Overall (2000 std.)** | 3.9% | 3.9% | 7,854 | 4.0% | 4.0% | 8,089 | 2.8% | 2.8% | 5,678 | 5.2% | 5.2% | 10,632 | 6.7% | 6.7% | 13,664 | 5,810 | 2.9% | **1.34 (0.14)** | **9.52** | **<.0001** | **0.27** | **5.46** |
| **Overall (2020 std.)** |  |  |  |  |  |  |  |  |  |  |  |  |  | 6.7% | 22,217 |  |  |  |  |  |  |  |
| **Age, years** |  |  |  |  |  |  |  |  |  |  |  |  |  |  |  |  |  | **1.73 (0.15)** | **11.52** | **<.0001** | **0.35** | **7.05** |
| 18-24 | 3.8% | 0.5% | 1,034 | 3.8% | 0.5% | 985 | 1.9% | 0.4% | 901 | 5.3% | 0.6% | 1,322 | 5.8% | 0.7% | 1,395 | 362 | 0.2% | **1.22 (0.4)** | **3.05** | **0.002** | 0.24 | 4.96 |
| 25-34 | 3.9% | 0.7% | 1,380 | 3.4% | 0.6% | 1,205 | 2.9% | 0.8% | 1,544 | 6.2% | 1.1% | 2,245 | 7.5% | 1.3% | 2,668 | 1,288 | 0.6% | **2.04 (0.26)** | **7.89** | **<.0001** | 0.41 | 8.32 |
| 35-44 | 4.4% | 0.9% | 1,880 | 4.0% | 0.7% | 1,521 | 2.9% | 0.6% | 1,162 | 5.1% | 0.8% | 1,716 | 7.7% | 1.3% | 2,658 | 778 | 0.4% | **1.43 (0.25)** | **5.67** | **<.0001** | 0.29 | 5.84 |
| 45-64 | 5.0% | 1.5% | 3,090 | 5.6% | 1.9% | 3,828 | 3.6% | 0.8% | 1,661 | 5.7% | 1.9% | 3,909 | 6.8% | 2.2% | 4,586 | 1,496 | 0.7% | **0.68 (0.21)** | **3.22** | **0.001** | 0.14 | 2.77 |
| 65 or above | 1.5% | 0.2% | 470 | 1.7% | 0.3% | 550 | 1.7% | 0.2% | 410 | 3.5% | 0.7% | 1,441 | 5.1% | 1.2% | 2,356 | 1,886 | 0.9% | **3.28 (0.36)** | **9.14** | **<.0001** | 0.66 | 13.38 |
| **Gender** |  |  |  |  |  |  |  |  |  |  |  |  |  |  |  |  |  | **1.29 (0.15)** | **8.93** | **<.0001** | **0.26** | **5.27** |
| Male | 3.0% | 1.4% | 2,905 | 3.1% | 1.4% | 2,939 | 2.0% | 0.7% | 1,494 | 3.8% | 1.8% | 3,762 | 4.6% | 2.3% | 4,636 | 1,730 | 0.8% | **1.08 (0.21)** | **5.21** | **<.0001** | 0.22 | 4.40 |
| Female | 4.8% | 2.4% | 4,948 | 5.0% | 2.5% | 5,150 | 3.5% | 2.1% | 4,183 | 6.5% | 3.4% | 6,870 | 8.5% | 4.4% | 9,029 | 4,080 | 2.0% | **1.51 (0.16)** | **9.19** | **<.0001** | 0.30 | 6.14 |
| **Ethnicity** |  |  |  |  |  |  |  |  |  |  |  |  |  |  |  |  |  | **1.4 (0.25)** | **5.65** | **<.0001** | **0.28** | **5.70** |
| White | 4.4% | 3.2% | 6,467 | 4.8% | 3.3% | 6,704 | 3.5% | 2.1% | 4,300 | 6.2% | 4.0% | 8,213 | 7.6% | 4.8% | 9,832 | 3,364 | 1.7% | **1.4 (0.16)** | **9.01** | **<.0001** | 0.28 | 5.71 |
| Hispanic | 1.9% | 0.2% | 428 | 2.2% | 0.3% | 584 | 1.1% | 0.3% | 522 | 2.6% | 0.4% | 844 | 4.0% | 0.7% | 1,431 | 1,003 | 0.5% | **1.67 (0.4)** | **4.19** | **<.0001** | 0.33 | 6.82 |
| Black | 2.7% | 0.3% | 599 | 2.1% | 0.2% | 483 | 1.3% | 0.2% | 411 | 4.4% | 0.5% | 1,091 | 5.3% | 0.6% | 1,271 | 672 | 0.3% | **2.21 (0.42)** | **5.31** | **<.0001** | 0.44 | 8.99 |
| Asian | 3.0% | 0.1% | 204 | 3.4% | 0.1% | 189 | 1.9% | 0.2% | 389 | 3.1% | 0.2% | 374 | 4.4% | 0.3% | 555 | 352 | 0.2% | 0.72 (0.67) | 1.07 | 0.280 | 0.14 | 2.92 |
| Others | 5.4% | 0.1% | 156 | 6.6% | 0.1% | 130 | 4.0% | 0.0% | 57 | 5.0% | 0.1% | 112 | 9.9% | 0.3% | 575 | 419 | 0.2% | 0.99 (0.8) | 1.24 | 0.216 | 0.20 | 4.05 |
| **Relationship Status** |  |  |  |  |  |  |  |  |  |  |  |  |  |  |  |  |  | **1.28 (0.14)** | **9.12** | **<.0001** | **0.26** | **5.21** |
| In a Relationship | 3.5% | 2.2% | 4,500 | 3.7% | 2.3% | 4,650 | 2.7% | 1.7% | 3,373 | 4.9% | 2.9% | 6,003 | 6.4% | 4.0% | 8,066 | 3,566 | 1.7% | **1.53 (0.18)** | **8.33** | **<.0001** | 0.31 | 6.23 |
| Not in a Relationship | 4.6% | 1.6% | 3,354 | 4.6% | 1.7% | 3,438 | 3.0% | 1.1% | 2,305 | 5.7% | 2.3% | 4,629 | 6.8% | 2.7% | 5,598 | 2,244 | 1.1% | **1.03 (0.19)** | **5.55** | **<.0001** | 0.21 | 4.20 |
| **Education** |  |  |  |  |  |  |  |  |  |  |  |  |  |  |  |  |  | **1.15 (0.18)** | **6.27** | **<.0001** | **0.23** | **4.68** |
| Less than high school | 1.1% | 0.2% | 377 | 1.2% | 0.2% | 383 | 0.7% | 0.1% | 176 | 1.9% | 0.2% | 470 | 2.2% | 0.2% | 496 | 119 | 0.1% | **1.83 (0.58)** | **3.14** | **0.001** | 0.37 | 7.47 |
| High school, some college | 6.7% | 1.9% | 3,906 | 5.7% | 1.9% | 3,772 | 4.4% | 1.2% | 2,527 | 10.0% | 2.3% | 4,672 | 9.2% | 2.9% | 5,917 | 2,011 | 1.0% | **1.1 (0.19)** | **5.72** | **<.0001** | 0.22 | 4.46 |
| Bachelor | 3.3% | 1.1% | 2,182 | 3.3% | 1.0% | 2,007 | 2.1% | 0.9% | 1,786 | 4.2% | 1.5% | 3,090 | 5.0% | 1.9% | 3,914 | 1,731 | 0.8% | **0.94 (0.22)** | **4.36** | **<.0001** | 0.19 | 3.82 |
| Master or higher | 8.7% | 0.7% | 1,388 | 10.7% | 0.9% | 1,927 | 7.5% | 0.6% | 1,189 | 9.4% | 1.2% | 2,401 | 12.8% | 1.6% | 3,337 | 1,949 | 1.0% | **0.73 (0.25)** | **2.89** | **0.001** | 0.15 | 2.96 |
| **Region** |  |  |  |  |  |  |  |  |  |  |  |  |  |  |  |  |  | **1.32 (0.14)** | **9.58** | **<.0001** | **0.26** | **5.36** |
| West | 5.8% | 1.1% | 2,244 | 5.6% | 1.2% | 2,487 | 4.4% | 0.9% | 1,774 | 6.8% | 1.6% | 3,347 | 8.5% | 2.1% | 4,193 | 1,950 | 1.0% | **1.02 (0.29)** | **3.52** | **0.001** | 0.20 | 4.17 |
| Northeast | 3.7% | 0.7% | 1,429 | 4.3% | 0.7% | 1,466 | 2.5% | 0.5% | 1,008 | 5.4% | 1.0% | 2,014 | 6.3% | 1.1% | 2,300 | 871 | 0.4% | **1.35 (0.28)** | **4.87** | **<.0001** | 0.27 | 5.51 |
| Midwest | 4.5% | 1.1% | 2,153 | 4.4% | 1.0% | 2,133 | 2.8% | 0.7% | 1,346 | 5.5% | 1.2% | 2,461 | 7.1% | 1.5% | 3,081 | 928 | 0.5% | **1.21 (0.29)** | **4.11** | **<.0001** | 0.24 | 4.92 |
| South | 2.7% | 1.0% | 2,028 | 2.7% | 1.0% | 2,002 | 2.0% | 0.8% | 1,549 | 3.8% | 1.4% | 2,811 | 5.2% | 2.0% | 4,090 | 2,062 | 1.0% | **1.68 (0.25)** | **6.65** | **<.0001** | 0.34 | 6.84 |
| **Health status** |  |  |  |  |  |  |  |  |  |  |  |  |  |  |  |  |  | **1.21 (0.18)** | **6.87** | **<.0001** | **0.24** | **4.93** |
| Excellent | 4.2% | 1.3% | 2,557 | 4.6% | 1.3% | 2,639 | 3.0% | 1.1% | 2,322 | 5.2% | 1.5% | 2,981 | 6.7% | 1.5% | 3,044 | 487 | 0.2% | **1.13 (0.23)** | **4.93** | **<.0001** | 0.23 | 4.60 |
| Very good | 3.6% | 1.3% | 2,589 | 4.0% | 1.3% | 2,575 | 2.9% | 1.0% | 2,038 | 5.5% | 1.8% | 3,667 | 6.0% | 2.4% | 4,975 | 2,386 | 1.2% | **1.44 (0.22)** | **6.55** | **<.0001** | 0.29 | 5.88 |
| Good | 3.6% | 0.9% | 1,811 | 3.6% | 0.9% | 1,851 | 2.2% | 0.5% | 1,021 | 4.8% | 1.3% | 2,634 | 6.5% | 2.0% | 3,978 | 2,167 | 1.1% | **1.56 (0.24)** | **6.49** | **<.0001** | 0.31 | 6.38 |
| Fair | 3.8% | 0.3% | 662 | 3.6% | 0.4% | 783 | 2.7% | 0.1% | 242 | 4.6% | 0.5% | 1,090 | 4.3% | 0.7% | 1,375 | 714 | 0.4% | **1.4 (0.38)** | **3.73** | **0.001** | 0.28 | 5.70 |
| Poor | 4.1% | 0.1% | 235 | 4.1% | 0.1% | 241 | 3.1% | 0.0% | 53 | 5.5% | 0.1% | 261 | 6.9% | 0.1% | 292 | 57 | 0.0% | 0.52 (0.59) | 0.88 | 0.380 | 0.10 | 2.11 |
| **Saw Mental Health Professional?** |  |  |  |  |  |  |  |  |  |  |  |  |  |  |  |  |  | **0.96 (0.16)** | **5.91** | **<.0001** | **0.19** | **3.92** |
| Yes | 13.0% | 0.8% | 1,630 | 15.0% | 1.0% | 2,021 | 10.9% | 0.4% | 773 | 16.3% | 1.5% | 3,015 | 18.1% | 2.3% | 4,774 | 3,144 | 1.5% | **0.89 (0.27)** | **3.35** | **0.001** | 0.18 | 3.64 |
| No | 3.3% | 3.1% | 6,223 | 3.3% | 3.0% | 6,068 | 2.2% | 2.4% | 4,904 | 4.1% | 3.7% | 7,618 | 4.9% | 4.4% | 8,890 | 2,667 | 1.3% | **1.03 (0.15)** | **6.71** | **<.0001** | 0.21 | 4.20 |
| **Psychological Distress (K6)** |  |  |  |  |  |  |  |  |  |  |  |  |  |  |  |  |  | **1.13 (0.21)** | **5.40** | **<.0001** | **0.23** | **4.59** |
| No/Mild distress | 3.5% | 3.1% | 6,384 | 2.5% | 3.3% | 6,625 | 4.6% | 2.6% | 5,219 | 4.6% | 4.1% | 8,323 | 5.4% | 4.6% | 9,340 | 2,957 | 1.5% | **1.15 (0.15)** | **7.46** | **<.0001** | 0.23 | 4.68 |
| Moderate distress | 8.8% | 0.5% | 1,023 | 5.8% | 0.4% | 878 | 9.6% | 0.2% | 335 | 9.6% | 0.7% | 1,517 | 11.5% | 1.4% | 2,916 | 1,892 | 0.9% | 0.84 (0.32) | 2.60 | 0.010 | 0.17 | 3.42 |
| Severe distress | 7.5% | 0.2% | 447 | 5.3% | 0.3% | 586 | 11.4% | 0.1% | 124 | 11.4% | 0.4% | 792 | 13.7% | 0.7% | 1,408 | 961 | 0.5% | **1.4 (0.48)** | **2.93** | **0.003** | 0.28 | 5.69 |
| **Physical Activity** |  |  |  |  |  |  |  |  |  |  |  |  |  |  |  |  |  | **0.92 (0.27)** | **3.41** | **0.00** | **0.18** | **3.77** |
| Inactive | 2.6% | 1.4% | 2,949 | 2.8% | 1.6% | 3,359 | 1.7% | 0.6% | 1,218 | 3.4% | 1.7% | 3,435 | 5.0% | 3.2% | 6,463 | 3,514 | 1.7% | **1.57 (0.2)** | **7.87** | **<.0001** | 0.31 | 6.40 |
| Yearly Exercise | 7.0% | 0.1% | 121 | 6.9% | 0.1% | 132 | 3.5% | 0.0% | 60 | 5.6% | 0.0% | 87 | 7.0% | 0.1% | 211 | 90 | 0.0% | -0.22 (1.06) | -0.21 | 0.830 | -0.04 | -0.91 |
| Monthly Exercise | 5.9% | 0.2% | 388 | 4.9% | 0.1% | 298 | 4.2% | 0.1% | 228 | 7.7% | 0.3% | 612 | 8.4% | 0.5% | 1,067 | 679 | 0.3% | 1.24 (0.6) | 2.08 | 0.0379 | 0.25 | 5.08 |
| Weekly Exercise | 5.8% | 1.7% | 3,563 | 6.2% | 1.9% | 3,791 | 4.1% | 1.7% | 3,501 | 7.0% | 2.7% | 5,595 | 9.6% | 2.7% | 5,409 | 1,846 | 0.9% | **1.23 (0.19)** | **6.54** | **<.0001** | 0.25 | 5.01 |
| Daily Exercise | 5.8% | 0.4% | 832 | 5.2% | 0.3% | 510 | 4.5% | 0.3% | 670 | 6.0% | 0.4% | 904 | 7.8% | 0.3% | 514 | -318 | 0.0% | 0.8 (0.49) | 1.65 | 0.099 | 0.16 | 3.27 |
| **Weight status** |  |  |  |  |  |  |  |  |  |  |  |  |  |  |  |  |  | **1.31 (0.24)** | **5.53** | **<.0001** | **0.26** | **5.35** |
| Healthy Weight | 4.5% | 1.6% | 3,345 | 4.4% | 1.5% | 3,090 | 3.6% | 1.5% | 2,970 | 6.0% | 1.9% | 3,946 | 7.7% | 2.4% | 4,955 | 1,610 | 0.8% | **1.49 (0.19)** | **7.73** | **<.0001** | 0.30 | 6.06 |
| Underweight | 5.1% | 0.1% | 197 | 4.3% | 0.1% | 150 | 2.4% | 0.1% | 151 | 4.9% | 0.1% | 178 | 8.0% | 0.1% | 276 | 79 | 0.0% | 1.08 (0.81) | 1.33 | 0.180 | 0.22 | 4.38 |
| Overweight | 3.6% | 1.2% | 2,404 | 3.6% | 1.2% | 2,464 | 2.7% | 0.8% | 1,618 | 5.0% | 1.7% | 3,445 | 6.0% | 2.1% | 4,181 | 1,777 | 0.9% | **1.41 (0.23)** | **6.04** | **<.0001** | 0.28 | 5.75 |
| Obese | 3.6% | 0.9% | 1,907 | 4.1% | 1.2% | 2,384 | 2.2% | 0.5% | 938 | 4.6% | 1.5% | 3,063 | 6.2% | 2.1% | 4,251 | 2,344 | 1.1% | **1.28 (0.23)** | **5.47** | **<.0001** | 0.26 | 5.22 |
| **Smoking Status** |  |  |  |  |  |  |  |  |  |  |  |  |  |  |  |  |  | **1.06 (0.18)** | **5.99** | **0.001** | **0.21** | **4.32** |
| Non-Smoker | 3.5% | 1.9% | 3,783 | 3.5% | 2.0% | 4,078 | 2.5% | 1.8% | 3,611 | 4.9% | 3.1% | 6,333 | 6.3% | 4.3% | 8,695 | 4,912 | 2.4% | **1.59 (0.18)** | **8.73** | **<.0001** | 0.32 | 6.50 |
| Former Smoker | 4.7% | 1.1% | 2,141 | 4.7% | 1.0% | 1,999 | 3.8% | 0.6% | 1,296 | 6.3% | 1.4% | 2,916 | 7.5% | 1.7% | 3,504 | 1,363 | 0.7% | **1.32 (0.22)** | **5.92** | **<.0001** | 0.26 | 5.38 |
| Current, Some Days | 5.6% | 0.2% | 450 | 5.7% | 0.2% | 500 | 4.2% | 0.1% | 285 | 5.3% | 0.2% | 378 | 8.1% | 0.2% | 450 | 0 | 0.0% | 0.72 (0.52) | 1.39 | 0.164 | 0.14 | 2.93 |
| Current, Daily | 4.0% | 0.7% | 1,479 | 4.9% | 0.7% | 1,512 | 2.4% | 0.2% | 485 | 4.7% | 0.5% | 1,005 | 5.5% | 0.5% | 1,015 | -465 | -0.2% | 0.61 (0.33) | 1.85 | 0.060 | 0.12 | 2.51 |
| **Alcohol Status** |  |  |  |  |  |  |  |  |  |  |  |  |  |  |  |  |  | **1.38 (0.21)** | **6.42** | **<.0001** | **0.28** | **5.61** |
| Lifetime Abstainer | 1.9% | 0.4% | 813 | 1.8% | 0.4% | 840 | 1.0% | 0.3% | 579 | 2.6% | 0.5% | 1,044 | 3.5% | 0.5% | 977 | 164 | 0.1% | **1.67 (0.47)** | **3.58** | **0.001** | 0.33 | 6.79 |
| Former Drinker | 3.7% | 0.5% | 1,105 | 3.8% | 0.5% | 1,104 | 2.5% | 0.2% | 474 | 5.3% | 0.7% | 1,455 | 6.1% | 1.1% | 2,165 | 1,060 | 0.5% | **1.39 (0.3)** | **4.61** | **<.0001** | 0.28 | 5.67 |
| Current Drinker | 4.8% | 2.9% | 5,936 | 5.1% | 3.0% | 6,144 | 3.5% | 2.3% | 4,625 | 6.0% | 4.0% | 8,133 | 7.3% | 5.2% | 10,521 | 4,586 | 2.2% | **1.07 (0.14)** | **7.47** | **<.0001** | 0.21 | 4.37 |

Population weighted prevalence estimates were calculated for each timepoint (2002, 2007, 2012, 2017, 2022) to represent the number (N, in thousands) of individuals who used the practice in the past 12 months as well as percentage estimates at both a subgroup level (i.e., with all individuals in that group in the population as the denominator) and at a whole population level (i.e., % of individuals in the whole population as the denominator). Changes over time are represented as: (a) 20-year numeric changes (i.e., 2022 prevalence minus 2002 prevalence); (b) 5-year regressed changes (β), and (c) 1-year regressed changes (i.e., β/5). For regressions at the group level (significance set at false discovery rate (FDR) adjusted *p*<0.05) and subgroup level, significance is shown in bold, FDR adjusted significance is denoted by (*) and non-significance by (^ns^). Text in green denotes the largest, and red denotes the smallest value in the group. We used the US population standard from the year 2000^24^ in all analyses to control for the effects of population growth. However, for 2022, a population estimate using the most recent population standard (2020) was applied to estimate the total number of individuals using the practice in 2022. Source: NHIS Data 2002-2022.

### Table S4. Deviation-coded main effects and 20-year-averaged rate contrasts for meditation, yoga and guided imagery/progressive relaxation by sociodemographic and health strata (Full Data)

|  | **Meditation** | | | | | | **Yoga** | | | | | | **Guided Imagery / Progressive Relaxation** | | | | | |
| --- | --- | --- | --- | --- | --- | --- | --- | --- | --- | --- | --- | --- | --- | --- | --- | --- | --- | --- |
|  | *Category Main effect* |  |  | *Category x Time Interaction* |  |  | *Category Main effect* |  |  | *Category x Time Interaction* |  |  | *Category Main effect* |  |  | *Category x Time Interaction* |  |  |
|  | OR (SE) | *z-ratio* | *p* | OR (SE) | *z-ratio* | *p* | OR (SE) |  | *p* | OR (SE) |  | *p* | OR (SE) |  | *p* | OR (SE) |  | *p* |
| **Age, years** | **F=84.51** |  | **<.001*** | **F=50.68** |  | **<.001*** | **F=1920.14** |  | **< .001*** | F=11.5 |  | .082^ns^ | **F=65.26** |  | **< .001*** | F=11.81 |  | .065^ns^ |
| 18-24 | 0.92 (0.03) | -2.51 | 0.012 | 0.68 (0.17) | -1.54 | 0.124 | **1.33 (0.04)** | **9.08** | **<.0001** | 1.19 (0.29) | 0.72 | 0.474 | 0.98 (0.05) | -0.42 | 0.672 | 1.13 (0.42) | 0.31 | 0.755 |
| 25-34 | **1.20 (0.03)** | **7.49** | **<.0001** | 0.67 (0.12) | -2.27 | 0.023 | **1.68 (0.04)** | **21.13** | **<.0001** | 0.77 (0.15) | -1.38 | 0.167 | **1.13 (0.04)** | **3.54** | **0.001** | 1.21 (0.31) | 0.75 | 0.456 |
| 35-44 | **1.09 (0.03)** | **3.24** | **0.001** | 0.75 (0.14) | -1.51 | 0.132 | **1.25 (0.03)** | **8.56** | **<.0001** | 0.70 (0.13) | -1.95 | 0.052 | **1.16 (0.04)** | **4.13** | **0.001** | 0.58 (0.16) | -2.02 | 0.043 |
| 45-64 | **1.20 (0.03)** | **8.46** | **<.0001** | 0.70 (0.10) | -2.49 | 0.013 | **0.94 (0.02)** | **-2.97** | **0.003** | 0.58 (0.10) | -3.07 | **0.002** | **1.34 (0.04)** | **9.27** | **<.0001** | 0.43 (0.09) | -4.06 | **<.0001** |
| 65 or above | **0.70 (0.02)** | **-12.02** | **<.0001** | **4.22 (0.91)** | **6.66** | **<.0001** | **0.38 (0.01)** | **-25.23** | **<.0001** | 2.71 (0.79) | 3.42 | **0.001** | **0.59 (0.03)** | **-12.54** | **<.0001** | 2.94 (0.92) | 3.44 | **0.001** |
| **Gender** | **F=499.09** |  | **<.001*** | F=3.96 |  | .604^ns^ | **F=2965.63** |  | **< .001*** | F=8.34 |  | .242^ns^ | **F=458.25** |  | **< .001*** | F=0.82 |  | .969^ns^ |
| Male | **0.83 (0.01)** | **-15.72** | **<.0001** | 0.96 (0.08) | -0.49 | 0.626 | **0.59 (0.01)** | **-35.35** | **<.0001** | 1.26 (0.14) | 2.09 | 0.037 | **0.76 (0.01)** | **-15.20** | **<.0001** | 0.89 (0.12) | -0.90 | 0.369 |
| Female | **1.21 (0.01)** | **15.72** | **<.0001** | 1.04 (0.09) | 0.49 | 0.626 | **1.69 (0.03)** | **35.35** | **<.0001** | 0.79 (0.09) | -2.09 | 0.037 | **1.32 (0.02)** | **15.20** | **<.0001** | 1.13 (0.15) | 0.90 | 0.369 |
| **Ethnicity** | **F=495.79** |  | **<.001*** | **F=61.14** |  | **.004*** | **F=1267.75** |  | **< .001*** | F=38.77 |  | .057^ns^ | **F=640.59** |  | **< .001*** | F=47.59 |  | **.035^ns^** |
| White | **1.10 (0.03)** | **3.12** | **0.002** | 1.19 (0.24) | 0.87 | 0.386 | **1.22 (0.04)** | **6.73** | **<.0001** | 1.70 (0.38) | 2.38 | 0.017 | **1.46 (0.06)** | **9.22** | **<.0001** | 1.08 (0.31) | 0.28 | 0.781 |
| Hispanic | **0.64 (0.03)** | **-10.99** | **<.0001** | 1.69 (0.5) | 1.79 | 0.073 | **0.6 (0.02)** | **-12.25** | **<.0001** | 1.75 (0.58) | 1.71 | 0.087 | **0.60 (0.04)** | **-7.84** | **<.0001** | 1.40 (0.65) | 0.73 | 0.468 |
| Black | 0.93 (0.04) | -1.86 | 0.062 | 1.29 (0.37) | 0.89 | 0.371 | **0.65 (0.03)** | **-10.68** | **<.0001** | 2.64 (0.88) | 2.93 | **0.003** | **0.79 (0.05)** | **-4.04** | **<.0001** | 2.84 (1.21) | 2.44 | 0.015 |
| Asian | 1.01 (0.06) | 0.14 | 0.888 | 0.93 (0.36) | -0.20 | 0.840 | **1.64 (0.08)** | **9.74** | **<.0001** | 1.13 (0.41) | 0.33 | 0.739 | 0.85 (0.07) | -2.04 | 0.042 | 0.57 (0.35) | -0.92 | 0.358 |
| Others | **1.53 (0.13)** | **5.16** | **<.0001** | 0.41 (0.22) | -1.69 | 0.091 | **1.28 (0.12)** | **2.70** | **0.007** | 0.11 (0.08) | -3.12 | **0.002** | **1.70 (0.19)** | **4.74** | **<.0001** | 0.41 (0.31) | -1.20 | 0.232 |
| **Relationship Status** | **F=328.05** |  | **< .001*** | **F=35.11** |  | **<.001*** | **F=80.16** |  | **< .001*** | F=8.61 |  | .174^ns^ | **F=14.68** |  | **0.002*** | F=7.40 |  | .276^ns^ |
| In a Relationship | **0.91 (0.01)** | **-8.17** | **<.0001** | **1.36 (0.12)** | **3.48** | **0.001** | 0.98 (0.01) | -1.31 | 0.190 | 1.19 (0.12) | 1.72 | 0.086 | **0.92 (0.02)** | **-4.90** | **<.0001** | 1.12 (0.15) | 0.87 | 0.384 |
| Not in a Relationship | **1.10 (0.01)** | **8.17** | **<.0001** | **0.74 (0.06)** | **-3.48** | **0.001** | 1.02 (0.01) | 1.31 | 0.190 | 0.84 (0.08) | -1.72 | 0.086 | **1.09 (0.02)** | **4.90** | **<.0001** | 0.89 (0.12) | -0.87 | 0.384 |
| **Education** | **F=1888.18** |  | **<.001*** | **F=97.64** |  | **<.001*** | **F=4157.66** |  | **< .001*** | F=17.35 |  | .528^ns^ | **F=1381.64** |  | **< .001*** | F=31.15 |  | .055^ns^ |
| Less than high school | **0.39 (0.02)** | **-22.98** | **<.0001** | **4.02 (1.12)** | **5.00** | **<.0001** | **0.21 (0.01)** | **-24.70** | **<.0001** | 2.87 (1.33) | 2.29 | 0.022 | **0.31 (0.02)** | **-16.91** | **<.0001** | 2.73 (1.31) | 2.10 | 0.036 |
| High school, some college | **0.88 (0.02)** | **-6.41** | **<.0001** | 0.84 (0.12) | -1.21 | 0.228 | **0.85 (0.02)** | **-6.25** | **<.0001** | 0.71 (0.14) | -1.73 | 0.083 | **0.82 (0.03)** | **-6.08** | **<.0001** | 1.07 (0.24) | 0.30 | 0.763 |
| Bachelor | **1.46 (0.03)** | **16.13** | **<.0001** | **0.46 (0.08)** | **-4.70** | **<.0001** | **2.14 (0.06)** | **26.68** | **<.0001** | 0.61 (0.13) | -2.34 | 0.019 | **1.59 (0.05)** | **13.43** | **<.0001** | 0.64 (0.16) | -1.84 | 0.066 |
| Master or higher | **2.01 (0.05)** | **25.98** | **<.0001** | 0.64 (0.13) | -2.21 | 0.027 | **2.66 (0.09)** | **30.42** | **<.0001** | 0.81 (0.19) | -0.90 | 0.370 | **2.46 (0.09)** | **23.89** | **<.0001** | 0.53 (0.14) | -2.44 | 0.015 |
| **Region** | **F=405.35** |  | **<.001*** | F=37.44 |  | .083^ns^ | **F=604.13** |  | **< .001*** | **F=40.77** |  | **.020*** | **F=376.62** |  | **< .001*** | F=18.79 |  | .646^ns^ |
| West | **1.3 (0.03)** | **10.13** | **<.0001** | 0.69 (0.14) | -1.84 | 0.065 | **1.29 (0.04)** | **9.12** | **<.0001** | 0.78 (0.16) | -1.26 | 0.206 | **1.41 (0.05)** | **10.13** | **<.0001** | 0.66 (0.17) | -1.60 | 0.109 |
| Northeast | 0.98 (0.03) | -0.88 | 0.377 | 0.82 (0.17) | -0.96 | 0.335 | **1.09 (0.03)** | **3.36** | **0.001** | 0.92 (0.17) | -0.45 | 0.654 | 0.96 (0.04) | -1.00 | 0.315 | 1.39 (0.37) | 1.22 | 0.221 |
| Midwest | 1.03 (0.03) | 1.23 | 0.218 | 0.95 (0.18) | -0.30 | 0.767 | 1.01 (0.03) | 0.19 | 0.853 | 1.23 (0.25) | 1.03 | 0.302 | 1.06 (0.04) | 1.43 | 0.153 | 0.92 (0.22) | -0.36 | 0.720 |
| South | **0.76 (0.02)** | **-9.99** | **<.0001** | 1.89 (0.36) | 3.35 | **0.001** | **0.71 (0.02)** | **-12.44** | **<.0001** | 1.14 (0.22) | 0.71 | 0.480 | **0.70 (0.03)** | **-9.63** | **<.0001** | 1.20 (0.31) | 0.70 | 0.482 |

| **Health status** | **F=115.5** |  | **<.001*** | **F=42.26** |  | **.025*** | **F=33.76** |  | **<.001*** | F=14.92 |  | .809^ns^ | **F=105.62** |  | **< .001*** | F=21.00 |  | .559^ns^ |
| --- | --- | --- | --- | --- | --- | --- | --- | --- | --- | --- | --- | --- | --- | --- | --- | --- | --- | --- |
| Excellent | 1.05 (0.03) | 2.01 | 0.045 | 0.93 (0.17) | -0.39 | 0.694 | **2.41 (0.08)** | **26.15** | **<.0001** | 1.17 (0.30) | 0.62 | 0.537 | **1.10 (0.04)** | **2.78** | **0.005** | 0.85 (0.21) | -0.67 | 0.506 |
| Very good | **1.11 (0.03)** | **4.62** | **<.0001** | 0.76 (0.13) | -1.61 | 0.107 | **1.79 (0.05)** | **19.61** | **<.0001** | 1.52 (0.35) | 1.80 | 0.071 | **1.09 (0.04)** | **2.69** | **0.007** | 1.03 (0.25) | 0.13 | 0.895 |
| Good | 0.94 (0.03) | -2.19 | 0.029 | 0.89 (0.17) | -0.63 | 0.530 | 1.04 (0.04) | 1.20 | 0.229 | 1.19 (0.33) | 0.61 | 0.542 | 0.93 (0.03) | -1.98 | 0.048 | 1.29 (0.35) | 0.95 | 0.343 |
| Fair | 0.97 (0.03) | -0.95 | 0.341 | 1.33 (0.33) | 1.17 | 0.241 | **0.62 (0.03)** | **-9.31** | **<.0001** | 1.07 (0.42) | 0.18 | 0.857 | 1.01 (0.05) | 0.29 | 0.774 | 1.31 (0.45) | 0.79 | 0.429 |
| Poor | 0.94 (0.05) | -1.05 | 0.295 | 1.19 (0.46) | 0.46 | 0.646 | **0.36 (0.03)** | **-11.27** | **<.0001** | 0.44 (0.32) | -1.12 | 0.263 | 0.88 (0.07) | -1.64 | 0.101 | 0.68 (0.36) | -0.74 | 0.461 |
| **Saw Mental Health Professional?** | **F=1518.7** |  | **<.001*** | **F=15.05** |  | **.029*** | **F=435.59** |  | **< .001*** | F=6.29 |  | .357^ns^ | **F=1276.33** |  | **< .001*** | F=9.92 |  | .223^ns^ |
| Yes | **1.88 (0.03)** | **40.57** | **<.0001** | **0.72 (0.09)** | **-2.65** | **0.008** | **1.52 (0.03)** | **23.05** | **<.0001** | 0.82 (0.11) | -1.45 | 0.147 | **2.18 (0.05)** | **37.71** | **<.0001** | 0.95 (0.15) | -0.36 | 0.723 |
| No | **0.53 (0.01)** | **-40.57** | **<.0001** | **1.39 (0.17)** | **2.65** | **0.008** | **0.66 (0.01)** | **-23.05** | **<.0001** | 1.22 (0.16) | 1.45 | 0.147 | **0.46 (0.01)** | **-37.71** | **<.0001** | 1.06 (0.17) | 0.36 | 0.723 |
| **Psychological Distress (K6)** | **F=309.27** |  | **<.001*** | F=17.26 |  | .207^ns^ | **F=35.68** |  | **< .001*** | F=6.49 |  | .801^ns^ | **F=300.43** |  | **< .001*** | F=17.87 |  | .219^ns^ |
| No/Mild distress | **0.62 (0.01)** | **-21.03** | **<.0001** | 1.38 (0.23) | 1.96 | 0.050 | 1.05 (0.03) | 1.61 | 0.107 | 0.94 (0.21) | -0.26 | 0.793 | **0.55 (0.02)** | **-19.82** | **<.0001** | 0.93 (0.21) | -0.35 | 0.728 |
| Moderate distress | **1.21 (0.04)** | **5.95** | **<.0001** | 0.79 (0.18) | -1.04 | 0.301 | **1.19 (0.05)** | **4.50** | **<.0001** | 1.11 (0.32) | 0.37 | 0.710 | **1.29 (0.05)** | **6.16** | **<.0001** | 0.63 (0.18) | -1.58 | 0.113 |
| Severe distress | **1.33 (0.05)** | **7.62** | **<.0001** | 0.92 (0.24) | -0.34 | 0.737 | **0.80 (0.04)** | **-4.14** | **<.0001** | 0.95 (0.38) | -0.12 | 0.906 | **1.42 (0.07)** | **7.08** | **<.0001** | 1.72 (0.59) | 1.56 | 0.118 |
| **Physical Activity** | **F=682.79** |  | **<.001*** | F=19.91 |  | .720^ns^ | **F=2276.18** |  | **< .001*** | F=36.61 |  | .104^ns^ | **F=290.81** |  | **< .001*** | F=17.71 |  | .814^ns^ |
| Inactive | **0.58 (0.02)** | **-15.40** | **<.0001** | 2.28 (0.57) | 3.32 | **0.001** | **0.40 (0.01)** | **-26.30** | **<.0001** | 1.59 (0.43) | 1.73 | 0.084 | **0.55 (0.03)** | **-12.32** | **<.0001** | 1.80 (0.61) | 1.72 | 0.085 |
| Yearly Exercise | 1.08 (0.13) | 0.64 | 0.520 | 0.98 (0.79) | -0.02 | 0.982 | 1.00 (0.10) | -0.05 | 0.964 | 0.49 (0.36) | -0.96 | 0.338 | 1.14 (0.16) | 0.93 | 0.354 | 0.45 (0.46) | -0.78 | 0.433 |
| Monthly Exercise | 1.15 (0.07) | 2.22 | 0.026 | 0.84 (0.36) | -0.42 | 0.677 | 1.12 (0.06) | 1.92 | 0.054 | 1.30 (0.56) | 0.62 | 0.538 | 1.17 (0.12) | 1.60 | 0.110 | 1.40 (0.72) | 0.66 | 0.507 |
| Weekly Exercise | **1.21 (0.04)** | **5.28** | **<.0001** | 1.09 (0.27) | 0.34 | 0.735 | **1.69 (0.06)** | **14.95** | **<.0001** | 1.07 (0.26) | 0.27 | 0.786 | **1.23 (0.06)** | **4.48** | **<.0001** | 1.29 (0.44) | 0.74 | 0.461 |
| Daily Exercise | **1.15 (0.06)** | **2.94** | **0.003** | 0.49 (0.16) | -2.16 | 0.031 | **1.35 (0.06)** | **6.83** | **<.0001** | 0.92 (0.31) | -0.26 | 0.794 | 1.11 (0.07) | 1.63 | 0.103 | 0.68 (0.31) | -0.85 | 0.396 |
| **Weight status** | F=11.24 |  | .062^ns^ | F=23.24 |  | .182^ns^ | **F=973.77** |  | **< .001*** | **F=32.77** |  | **.029*** | F=1.58 |  | .812^ns^ | F=20.22 |  | .393^ns^ |
| Healthy Weight | 1.17 (0.03) | 5.87 | **<.0001** | 0.92 (0.18) | -0.42 | 0.677 | **1.64 (0.05)** | **17.47** | **<.0001** | 0.82 (0.17) | -0.94 | 0.348 | 1.16 (0.05) | 3.79 | **<.0001** | 1.03 (0.27) | 0.12 | 0.908 |
| Underweight | 0.94 (0.06) | -0.95 | 0.345 | 1.23 (0.59) | 0.43 | 0.670 | **1.36 (0.09)** | **4.53** | **<.0001** | 1.29 (0.64) | 0.53 | 0.599 | 1.05 (0.10) | 0.57 | 0.572 | 0.69 (0.44) | -0.59 | 0.554 |
| Overweight | 0.96 (0.03) | -1.28 | 0.199 | 0.93 (0.19) | -0.36 | 0.717 | **0.87 (0.02)** | **-5.01** | **<.0001** | 0.72 (0.15) | -1.61 | 0.108 | 0.92 (0.04) | -1.91 | 0.056 | 1.19 (0.33) | 0.62 | 0.533 |
| Obese | 0.94 (0.03) | -2.00 | 0.046 | 0.95 (0.20) | -0.25 | 0.805 | **0.52 (0.02)** | **-21.26** | **<.0001** | 1.32 (0.31) | 1.19 | 0.236 | 0.89 (0.04) | -2.97 | **0.003** | 1.19 (0.34) | 0.59 | 0.554 |
| **Smoking Status** | **F=330.98** |  | **<.001*** | **F=64.4** |  | **<.001*** | **F=385.98** |  | **< .001*** | F=25.44 |  | .161^ns^ | **F=253.17** |  | **< .001*** | F=22.80 |  | .271^ns^ |
| Non-Smoker | **0.85 (0.02)** | **-7.90** | **<.0001** | **2.15 (0.30)** | **5.44** | **<.0001** | **1.15 (0.03)** | **6.17** | **<.0001** | 2.02 (0.33) | 4.34 | **<.0001** | **0.83 (0.03)** | **-6.11** | **<.0001** | 1.6 (0.33) | 2.25 | 0.024 |
| Former Smoker | **1.07 (0.03)** | **2.73** | **0.006** | 1.44 (0.25) | 2.15 | 0.032 | **1.11 (0.03)** | **4.02** | **<.0001** | 1.28 (0.25) | 1.25 | 0.213 | **1.13 (0.04)** | **3.59** | **<.0001** | 1.21 (0.30) | 0.77 | 0.440 |
| Current, Some Days | **1.30 (0.06)** | **6.04** | **<.0001** | **0.40 (0.12)** | **-3.01** | **0.003** | **1.37 (0.06)** | **7.17** | **<.0001** | **0.44 (0.14)** | **-2.51** | **0.012** | **1.21 (0.07)** | **3.19** | **0.001** | 0.50 (0.22) | -1.54 | 0.123 |
| Current, Daily | **0.85 (0.02)** | **-5.62** | **<.0001** | 0.82 (0.17) | -0.96 | 0.336 | **0.57 (0.02)** | **-15.42** | **<.0001** | 0.89 (0.24) | -0.43 | 0.668 | **0.88 (0.04)** | **-2.95** | **0.003** | 1.03 (0.32) | 0.09 | 0.929 |
| **Alcohol Status** | **F=281.97** |  | **<.001*** | **F=48.02** |  | **<.001*** | **F=793.20** |  | **< .001*** | F=15.93 |  | .256^ns^ | **F=175.95** |  | **< .001*** | F=7.99 |  | .765^ns^ |
| Lifetime Abstainer | **0.63 (0.02)** | **-16.03** | **<.0001** | **2.29 (0.45)** | **4.16** | **<.0001** | **0.68 (0.02)** | **-11.30** | **<.0001** | 1.69 (0.42) | 2.13 | 0.034 | **0.57 (0.02)** | **-12.98** | **<.0001** | 1.29 (0.42) | 0.78 | 0.438 |
| Former Drinker | **1.20 (0.03)** | **7.47** | **<.0001** | 0.76 (0.13) | -1.65 | 0.099 | **0.79 (0.03)** | **-6.85** | **<.0001** | 0.61 (0.15) | -2.07 | 0.039 | **1.17 (0.04)** | **4.37** | **<.0001** | 1.12 (0.29) | 0.44 | 0.661 |
| Current Drinker | **1.32 (0.03)** | **14.15** | **<.0001** | **0.58 (0.08)** | **-4.13** | **<.0001** | **1.86 (0.04)** | **27.13** | **<.0001** | 0.97 (0.16) | -0.20 | 0.841 | **1.51 (0.04)** | **13.88** | **<.0001** | 0.69 (0.15) | -1.67 | 0.096 |

Omnibus test results for each category (F-value, p-value) and deviation-coded main effects and 20-year-averaged rate contrasts (both expressed as odds ratio with standard errors, OR (SE), z-ratio, p-value) are shown for meditation, yoga, and guided imagery/progressive relaxation by sociodemographic and health group and subgroup. For all F-tests, significance was set at false discovery rate (FDR) adjusted *p*<0.05 using the Benjamini-Hochberg method, with unadjusted significance shown in bold, FDR adjusted significance is denoted by (*) and non-significance by (^ns^). For all *post hoc* deviation contrasts, significant p-values following Bonferroni correction are shown in bold. Source: NHIS Data 2002-2022.

### Table S5. Interactions and pairwise-contrasts for meditation, yoga, and guided imagery/progressive relaxation by sociodemographic and health strata (Full Data)

|  | **Meditation** |  |  | **Yoga** |  |  | **Guided Imagery / Progressive Relaxation** |  |  |
| --- | --- | --- | --- | --- | --- | --- | --- | --- | --- |
|  | *Category x Time Interaction* |  |  | *Category x Time Interaction* |  |  | *Category x Time Interaction* |  |  |
|  | OR (SE) | *z-ratio* | *p* | OR (SE) |  | *p* | OR (SE) |  | *p* |
| **Age, years** | **F=50.68** |  | **< .001** | F=11.50 |  | 0.082 | F=11.81 |  | 0.065 |
| (18-24) - (25-34) | -0.15 (0.3) | -0.52 | 0.604 | 0.06 (0.34) | 0.16 | 0.871 | -0.82 (0.46) | -1.80 | 0.072 |
| (18-24) - (35-44) | -0.3 (0.31) | -0.95 | 0.345 | -0.14 (0.34) | -0.43 | 0.670 | -0.21 (0.47) | -0.46 | 0.646 |
| (18-24) - (45-64) | 0.41 (0.28) | 1.46 | 0.145 | 0.52 (0.33) | 1.57 | 0.116 | 0.54 (0.43) | 1.25 | 0.211 |
| (18-24) – 65+ | **-1.31 (0.32)** | **-4.05** | **0.000** | **-1.33 (0.43)** | **-3.13** | **0.002** | **-2.07 (0.52)** | **-3.95** | **0.000** |
| (25-34) - (35-44) | -0.14 (0.24) | -0.58 | 0.559 | -0.2 (0.27) | -0.75 | 0.454 | 0.61 (0.35) | 1.74 | 0.083 |
| (25-34) - (45-64) | **0.57 (0.21)** | **2.68** | **0.007** | 0.47 (0.25) | 1.89 | 0.059 | **1.36 (0.31)** | **4.33** | **0.000** |
| (25-34) – 65+ | **-1.16 (0.26)** | **-4.51** | **0.000** | **-1.39 (0.39)** | **-3.53** | **0.000** | **-1.24 (0.45)** | **-2.78** | **0.005** |
| (35-44) - (45-64) | **0.71 (0.22)** | **3.25** | **0.001** | **0.66 (0.25)** | **2.67** | **0.008** | 0.75 (0.31) | 2.41 | 0.016 |
| (35-44) – 65+ | **-1.01 (0.27)** | **-3.73** | **0.000** | **-1.19 (0.39)** | **-3.02** | **0.003** | **-1.85 (0.43)** | **-4.27** | **0.000** |
| (45-64) – 65+ | **-1.72 (0.24)** | **-7.24** | **0.000** | **-1.85 (0.38)** | **-4.91** | **0.000** | **-2.6 (0.4)** | **-6.49** | **0.000** |
| **Gender** | F=3.96 |  | 0.604 | F=8.34 |  | 0.242 | F=0.82 |  | 0.969 |
| Male - Female | -0.34 (0.15) | -2.21 | 0.027 | **0.6 (0.22)** | **2.76** | **0.006** | -0.43 (0.24) | -1.81 | 0.070 |
| **Ethnicity** | **F=61.14** |  | **0.004** | F=38.77 |  | 0.057 | F=47.59 |  | 0.036 |
| (Non-Hispanic White) - Hispanic | -0.09 (0.29) | -0.31 | 0.757 | -0.51 (0.31) | -1.65 | 0.099 | -0.27 (0.41) | -0.66 | 0.506 |
| (Non-Hispanic White) - (Non-Hispanic Black) | 0.33 (0.26) | 1.26 | 0.209 | **-0.86 (0.33)** | **-2.61** | **0.009** | -0.8 (0.44) | -1.82 | 0.069 |
| (Non-Hispanic White) - (Non-Hispanic Other) | **1.45 (0.53)** | **2.72** | **0.007** | **1.88 (0.66)** | **2.86** | **0.004** | 0.41 (0.82) | 0.50 | 0.619 |
| (Non-Hispanic White) - (Non-Hispanic Asian) | 0.71 (0.42) | 1.67 | 0.094 | 0.5 (0.39) | 1.28 | 0.200 | 0.68 (0.68) | 1.00 | 0.318 |
| Hispanic - (Non-Hispanic Black) | 0.42 (0.36) | 1.16 | 0.246 | -0.35 (0.42) | -0.83 | 0.407 | -0.53 (0.58) | -0.92 | 0.358 |
| Hispanic - (Non-Hispanic Other) | **1.54 (0.58)** | **2.66** | **0.008** | **2.39 (0.71)** | **3.35** | **0.001** | 0.68 (0.88) | 0.77 | 0.441 |
| Hispanic - (Non-Hispanic Asian) | 0.8 (0.48) | 1.67 | 0.095 | 1.01 (0.48) | 2.09 | 0.036 | 0.95 (0.78) | 1.22 | 0.223 |
| (Non-Hispanic Black) - (Non-Hispanic Other) | 1.12 (0.57) | 1.98 | 0.048 | **2.74 (0.72)** | **3.80** | **0.000** | 1.21 (0.9) | 1.35 | 0.177 |
| (Non-Hispanic Black) - (Non-Hispanic Asian) | 0.38 (0.47) | 0.81 | 0.416 | **1.36 (0.48)** | **2.83** | **0.005** | 1.49 (0.8) | 1.87 | 0.062 |
| (Non-Hispanic Other) - (Non-Hispanic Asian) | -0.74 (0.65) | -1.14 | 0.253 | -1.38 (0.77) | -1.79 | 0.073 | 0.28 (1.03) | 0.27 | 0.788 |
| **Relationship Status** | **F=35.11** |  | **< .001** | F=8.61 |  | 0.174 | F=7.40 |  | 0.276 |
| In a relationship - Not in a relationship | **0.76 (0.16)** | **4.87** | **0.000** | 0.44 (0.19) | 2.35 | 0.019 | 0.5 (0.24) | 2.08 | 0.038 |
| **Education** | **F=97.64** |  | **< .001** | F=17.35 |  | 0.528 | F=31.15 |  | 0.055 |
| Less than high school - High school, some college | **0.98 (0.36)** | **2.75** | **0.006** | **1.64 (0.63)** | **2.62** | **0.009** | 0.74 (0.61) | 1.21 | 0.226 |
| Less than high school - Bachelor | 0.93 (0.37) | 2.52 | 0.012 | **1.71 (0.64)** | **2.67** | **0.008** | 0.89 (0.62) | 1.44 | 0.151 |
| Less than high school - Master or higher | 0.56 (0.39) | 1.44 | 0.150 | 1.47 (0.65) | 2.28 | 0.023 | 1.11 (0.63) | 1.76 | 0.078 |
| High school, some college - Bachelor | -0.06 (0.19) | -0.30 | 0.762 | 0.06 (0.2) | 0.30 | 0.761 | 0.16 (0.27) | 0.58 | 0.562 |
| High school, some college - Master or higher | -0.42 (0.24) | -1.78 | 0.076 | -0.17 (0.27) | -0.65 | 0.516 | 0.37 (0.29) | 1.26 | 0.208 |
| Bachelor - Master or higher | -0.36 (0.26) | -1.39 | 0.163 | -0.24 (0.27) | -0.87 | 0.383 | 0.21 (0.31) | 0.68 | 0.494 |
| **Region** | F=37.44 |  | 0.083 | F=40.77 |  | 0.019 | F=18.79 |  | 0.646 |
| West - Northeast | 0.07 (0.31) | 0.21 | 0.832 | -0.13 (0.31) | -0.43 | 0.667 | -0.33 (0.4) | -0.82 | 0.410 |
| West - Midwest | -0.04 (0.29) | -0.14 | 0.891 | -0.62 (0.32) | -1.97 | 0.049 | -0.18 (0.41) | -0.45 | 0.656 |
| West - South | -0.68 (0.27) | -2.54 | 0.011 | -0.66 (0.31) | -2.15 | 0.031 | -0.66 (0.39) | -1.67 | 0.094 |
| Northeast - Midwest | -0.1 (0.29) | -0.36 | 0.722 | -0.49 (0.31) | -1.59 | 0.112 | 0.15 (0.4) | 0.36 | 0.716 |
| Northeast - South | **-0.74 (0.28)** | **-2.64** | **0.008** | -0.53 (0.29) | -1.80 | 0.071 | -0.33 (0.39) | -0.84 | 0.399 |
| Midwest - South | -0.64 (0.26) | -2.42 | 0.015 | -0.03 (0.3) | -0.11 | 0.909 | -0.47 (0.39) | -1.22 | 0.222 |
| Health status | F= 42.26 |  | 0.025 | F=14.92 |  | 0.809 | F=21.00 |  | 0.559 |
| Excellent - Very good | -0.13 (0.19) | -0.65 | 0.518 | -0.39 (0.23) | -1.71 | 0.087 | -0.32 (0.3) | -1.04 | 0.300 |
| Excellent - Good | 0.16 (0.23) | 0.69 | 0.492 | -0.05 (0.28) | -0.17 | 0.867 | -0.44 (0.32) | -1.37 | 0.170 |
| Excellent - Fair | 0.13 (0.3) | 0.42 | 0.672 | -0.19 (0.44) | -0.42 | 0.672 | -0.27 (0.43) | -0.63 | 0.530 |
| Excellent - Poor | 0.8 (0.45) | 1.79 | 0.074 | 0.34 (0.83) | 0.41 | 0.681 | 0.61 (0.62) | 0.98 | 0.327 |
| Very good - Good | 0.29 (0.21) | 1.37 | 0.171 | 0.34 (0.27) | 1.27 | 0.206 | -0.12 (0.3) | -0.40 | 0.686 |
| Very good - Fair | 0.25 (0.28) | 0.90 | 0.368 | 0.2 (0.43) | 0.46 | 0.642 | 0.04 (0.41) | 0.11 | 0.914 |
| Very good - Poor | 0.93 (0.43) | 2.13 | 0.033 | 0.73 (0.82) | 0.89 | 0.375 | 0.93 (0.62) | 1.50 | 0.133 |
| Good - Fair | -0.03 (0.3) | -0.11 | 0.912 | -0.14 (0.46) | -0.31 | 0.760 | 0.17 (0.42) | 0.39 | 0.696 |
| Good - Poor | 0.64 (0.46) | 1.40 | 0.160 | 0.39 (0.84) | 0.46 | 0.645 | 1.05 (0.62) | 1.68 | 0.093 |
| Fair - Poor | 0.67 (0.49) | 1.36 | 0.173 | 0.53 (0.9) | 0.59 | 0.554 | 0.88 (0.69) | 1.28 | 0.199 |
| **Saw Mental Health Professional?** | F=15.05 |  | 0.029 | F=6.29 |  | 0.358 | F=9.92 |  | 0.223 |
| Saw professional for MH - Didn't see a professional for MH | -0.33 (0.21) | -1.57 | 0.117 | -0.26 (0.26) | -0.99 | 0.323 | -0.14 (0.29) | -0.48 | 0.629 |
| **Psychological Distress (K6)** | F=17.26 |  | 0.207 | F=6.49 |  | 0.801 | F=17.87 |  | 0.219 |
| (No/mild mental distress) - Moderate mental distress | **0.68 (0.25)** | **2.69** | **0.007** | -0.11 (0.31) | -0.37 | 0.714 | 0.31 (0.34) | 0.91 | 0.363 |
| (No/mild mental distress) - Serious mental distress | 0.49 (0.33) | 1.50 | 0.135 | -0.79 (0.57) | -1.38 | 0.168 | -0.25 (0.49) | -0.51 | 0.608 |
| Moderate mental distress - Serious mental distress | -0.19 (0.39) | -0.48 | 0.634 | -0.68 (0.65) | -1.04 | 0.297 | -0.56 (0.57) | -0.99 | 0.325 |
| **Physical Activity** | F=19.91 |  | 0.719 | F=36.61 |  | 0.104 | F=17.71 |  | 0.814 |
| Inactive - Yearly exercise | 0.24 (0.8) | 0.30 | 0.764 | 0.76 (0.88) | 0.87 | 0.383 | 1.79 (1.09) | 1.65 | 0.099 |
| Inactive - Monthly exercise | 0.59 (0.41) | 1.43 | 0.153 | -0.21 (0.49) | -0.43 | 0.671 | 0.32 (0.62) | 0.53 | 0.598 |
| Inactive - Weekly exercise | **0.51 (0.17)** | **3.10** | **0.002** | 0.22 (0.21) | 1.05 | 0.294 | 0.34 (0.25) | 1.35 | 0.177 |
| Inactive - Daily exercise | **1.3 (0.34)** | **3.80** | **0.000** | 0.56 (0.37) | 1.52 | 0.128 | 0.77 (0.51) | 1.51 | 0.131 |
| Yearly exercise - Monthly exercise | 0.35 (0.89) | 0.39 | 0.696 | -0.97 (0.95) | -1.02 | 0.306 | -1.47 (1.22) | -1.20 | 0.229 |
| Yearly exercise - Weekly exercise | 0.27 (0.8) | 0.34 | 0.731 | -0.54 (0.87) | -0.62 | 0.533 | -1.45 (1.09) | -1.34 | 0.182 |
| Yearly exercise - Daily exercise | 1.06 (0.86) | 1.23 | 0.218 | -0.2 (0.94) | -0.22 | 0.829 | -1.02 (1.17) | -0.88 | 0.380 |
| Monthly exercise - Weekly exercise | -0.07 (0.4) | -0.18 | 0.856 | 0.43 (0.47) | 0.91 | 0.362 | 0.01 (0.6) | 0.02 | 0.980 |
| Monthly exercise - Daily exercise | 0.71 (0.52) | 1.36 | 0.174 | 0.77 (0.56) | 1.38 | 0.168 | 0.44 (0.78) | 0.57 | 0.568 |
| Weekly exercise - Daily exercise | 0.78 (0.34) | 2.32 | 0.020 | 0.34 (0.35) | 0.97 | 0.330 | 0.43 (0.52) | 0.83 | 0.409 |
| **Smoking Status** | **F=64.40** |  | **< .001** | F=25.44 |  | 0.161 | F=22.80 |  | 0.271 |
| (Non-smoker) - Former smoker | **0.47 (0.18)** | **2.58** | **0.010** | **0.71 (0.21)** | **3.38** | **0.001** | 0.28 (0.27) | 1.03 | 0.303 |
| (Non-smoker) - Current some day smoker | **1.36 (0.37)** | **3.69** | **0.000** | **1.79 (0.43)** | **4.21** | **0.000** | 0.88 (0.54) | 1.62 | 0.104 |
| (Non-smoker) - Current daily smoker | **1.47 (0.24)** | **6.20** | **0.000** | **1.19 (0.33)** | **3.64** | **0.000** | **0.98 (0.36)** | **2.71** | **0.007** |
| Former smoker - Current some day smoker | 0.89 (0.38) | 2.32 | 0.020 | 1.09 (0.46) | 2.37 | 0.018 | 0.6 (0.57) | 1.05 | 0.293 |
| Former smoker - Current daily smoker | **1 (0.24)** | **4.14** | **0.000** | 0.49 (0.35) | 1.40 | 0.162 | 0.7 (0.37) | 1.89 | 0.058 |
| Current some day smoker - Current daily smoker | 0.11 (0.41) | 0.27 | 0.788 | -0.6 (0.52) | -1.16 | 0.244 | 0.1 (0.61) | 0.17 | 0.866 |
| **Alcohol Status** | **F=48.02** |  | **< .001** | F=15.93 |  | 0.256 | F=7.99 |  | 0.765 |
| Lifetime Abstainer - Former drinker | **1.06 (0.32)** | **3.32** | **0.001** | **1.28 (0.43)** | **2.94** | **0.003** | 0.28 (0.51) | 0.54 | 0.589 |
| Lifetime Abstainer - Current drinker | **0.98 (0.29)** | **3.43** | **0.001** | **1.07 (0.35)** | **3.11** | **0.002** | 0.59 (0.46) | 1.29 | 0.199 |
| Former drinker - Current drinker | -0.08 (0.21) | -0.39 | 0.695 | -0.2 (0.31) | -0.66 | 0.509 | 0.32 (0.31) | 1.02 | 0.310 |

Group x time interactions (F value, significance set at *p*<0.001) and pairwise contrasts (expressed as odds ratio with standard errors, OR (SE), z-ratio, significance set at *p*<0.01) are shown for meditation, yoga, and guided imagery/progressive relaxation by sociodemographic and health group. Results are exploratory and were not reported in the main paper, therefore they have not been FDR adjusted. Source: NHIS Data 2002-2022.

### Table S6. Chi Square results comparing total population subgroup prevalence with meditation, yoga, and guided imagery/progressive relaxation subgroup prevalence (Full Data)

|  | **US adult population** | **Meditation users** | | **Yoga users** | | **Guided Imagery / Progressive Relaxation users** | |
| --- | --- | --- | --- | --- | --- | --- | --- |
| **Characteristic** | **%** | **%** | **Chi-square** | **%** | **Chi-square** | **%** | **Chi-square** |
| **Age, years** |  |  |  |  |  |  |  |
| 18-24 | 12.88 | 12.21 *** | 28.59 [-0.02, -0.01] | 13.50 *** | 21.29 [0.01, 0.02] | 10.21 *** | 105.69 [-0.02, -0.01] |
| 25-34 | 18.26 | 21.23 *** | 434.47 [0.05, 0.06] | 25.56 *** | 2369.32 [0.12, 0.13] | 19.53 *** | 18.06 [0, 0.01] |
| 35-44 | 21.91 | 25.31 *** | 498.68 [0.05, 0.06] | 21.17 *** | 19.64 [-0.02, -0.01] | 19.45 *** | 59.03 [-0.01, -0.01] |
| 45-64 | 29.92 | 28.07 *** | 116.09 [-0.03, -0.02] | 28.47 *** | 61.55 [-0.02, -0.01] | 33.56 *** | 107.48 [0.01, 0.02] |
| 65 or above | 17.03 | 13.18 *** | 722.46 [-0.08, -0.07] | 11.30 *** | 1372.26 [-0.1, -0.09] | 17.24 ^ns^ | 0.53 [0, 0] |
| **Gender** |  |  |  |  |  |  |  |
| Male | 51.36 | 39.30 *** | 4197.49 [-0.14, -0.13] | 29.81 *** | 11697.29 [-0.22, -0.21] | 33.93 *** | 2060.76 [-0.06, -0.05] |
| Female | 48.64 | 60.70 *** | 4197.5 [0.13, 0.14] | 70.19 *** | 11697.29 [0.21, 0.22] | 66.07 *** | 2060.76 [0.05, 0.06] |
| **Ethnicity** |  |  |  |  |  |  |  |
| White | 62.54 | 66.69 *** | 523.35 [0.04, 0.05] | 68.17 *** | 832.55 [0.05, 0.06] | 71.95 *** | 634.89 [0.03, 0.03] |
| Hispanic | 17.15 | 12.28 *** | 1140.74 [-0.09, -0.08] | 11.60 *** | 1282.97 [-0.09, -0.09] | 10.47 *** | 518.14 [-0.04, -0.03] |
| Black | 11.46 | 11.40 ^ns^ | 0.21 [-0.01, 0] | 8.84 *** | 402.44 [-0.07, -0.05] | 9.30 *** | 76.62 [-0.02, -0.01] |
| Asian | 6.06 | 6.09 ^ns^ | 0.12 [-0.01, 0.01] | 8.39 *** | 640.96 [0.1, 0.12] | 4.06 *** | 115 [-0.03, -0.02] |
| Others | 2.80 | 3.54 *** | 152.1 [0.06, 0.09] | 3.00 ** | 9.1 [0.01, 0.03] | 4.21 *** | 127.54 [0.03, 0.05] |
| **Relationship status** |  |  |  |  |  |  |  |
| In a Relationship | 60.42 | 58.88 *** | 71.85 [-0.02, -0.01] | 61.65 *** | 39.16 [0.01, 0.02] | 59.03 *** | 13.71 [-0.01, 0] |
| Not in a Relationship | 39.58 | 41.12 *** | 71.85 [0.01, 0.02] | 38.35 *** | 39.16 [-0.02, -0.01] | 40.97 *** | 13.71 [0, 0.01] |
| **Education** |  |  |  |  |  |  |  |
| Less than high school | 10.60 | 4.42 *** | 2597.61 [-0.16, -0.16] | 2.58 *** | 3724.05 [-0.19, -0.18] | 3.63 *** | 832.1 [-0.06, -0.05] |
| High school, some college | 56.55 | 47.00 *** | 2698.64 [-0.11, -0.1] | 41.25 *** | 6049.27 [-0.16, -0.15] | 43.30 *** | 1213.8 [-0.04, -0.04] |
| Bachelor | 20.33 | 27.19 *** | 2214.1 [0.12, 0.13] | 32.39 *** | 6152.08 [0.19, 0.21] | 28.64 *** | 737.34 [0.04, 0.04] |
| Master or higher | 12.52 | 21.39 *** | 5983.18 [0.25, 0.26] | 23.78 *** | 8583.79 [0.29, 0.31] | 24.42 *** | 2325.59 [0.08, 0.09] |
| **Region** |  |  |  |  |  |  |  |
| West | 23.79 | 28.99 *** | 1107.02 [0.08, 0.09] | 27.04 *** | 369.78 [0.04, 0.05] | 30.69 *** | 449.98 [0.03, 0.03] |
| Northeast | 17.43 | 17.33 ^ns^ | 0.44 [-0.01, 0] | 19.44 *** | 177.72 [0.03, 0.04] | 16.83 ^ns^ | 4.09 [-0.01, 0] |
| Midwest | 20.77 | 20.79 ^ns^ | 0.02 [0, 0.01] | 22.14 *** | 71.55 [0.02, 0.03] | 22.55 *** | 32.33 [0.01, 0.01] |
| South | 38.01 | 32.88 *** | 790.31 [-0.06, -0.05] | 31.37 *** | 1146.23 [-0.07, -0.06] | 29.93 *** | 465.28 [-0.03, -0.02] |

| **Health status** |  |  |  |  |  |  |  |
| --- | --- | --- | --- | --- | --- | --- | --- |
| Excellent | 21.73 | 22.53 *** | 27.27 [0.01, 0.02] | 31.00 *** | 3355.31 [0.14, 0.15] | 22.28 ^ns^ | 2.95 [0, 0.01] |
| Very good | 34.50 | 39.23 *** | 723.23 [0.05, 0.06] | 41.54 *** | 1394.23 [0.07, 0.08] | 36.41 *** | 27.21 [0, 0.01] |
| Good | 29.46 | 26.57 *** | 285.25 [-0.04, -0.03] | 21.42 *** | 1872.73 [-0.1, -0.09] | 29.11 ^ns^ | 0.94 [0, 0] |
| Fair | 11.04 | 9.37 *** | 197.87 [-0.05, -0.04] | 5.19 *** | 1980.67 [-0.14, -0.13] | 10.07 *** | 16.15 [-0.01, 0] |
| Poor | 3.27 | 2.30 *** | 202.47 [-0.09, -0.07] | 0.84 *** | 1015.12 [-0.17, -0.16] | 2.13 *** | 67.11 [-0.03, -0.02] |
| **Saw Mental Health Professional?** |  |  |  |  |  |  |  |
| Yes | 12.66 | 26.26 *** | 15291.23 [0.42, 0.44] | 22.73 *** | 6642.03 [0.25, 0.27] | 34.94 *** | 8538.04 [0.17, 0.18] |
| No | 87.34 | 73.74 *** | 15291.21 [-0.44, -0.42] | 77.27 *** | 6642.03 [-0.27, -0.25] | 65.06 *** | 8538.04 [-0.18, -0.17] |
| **Psychological Distress (K6)** |  |  |  |  |  |  |  |
| No/Mild distress | 82.92 | 75.40 *** | 3115.9 [-0.16, -0.15] | 80.96 *** | 171.59 [-0.04, -0.03] | 68.36 *** | 2664.11 [-0.09, -0.08] |
| Moderate distress | 12.12 | 16.95 *** | 1699.2 [0.13, 0.14] | 13.97 *** | 203.76 [0.04, 0.05] | 21.34 *** | 1414.86 [0.07, 0.07] |
| Severe distress | 4.95 | 7.65 *** | 1253.35 [0.17, 0.19] | 5.07 ^ns^ | 1.72 [0, 0.01] | 10.30 *** | 1107.76 [0.09, 0.1] |
| **Physical Activity** |  |  |  |  |  |  |  |
| Inactive | 61.99 | 49.34 *** | 5032.2 [-0.16, -0.15] | 35.88 *** | 19252.06 [-0.29, -0.28] | 47.30 *** | 1565.08 [-0.05, -0.05] |
| Yearly Exercise | 1.45 | 1.92 *** | 122.3 [0.08, 0.12] | 1.65 *** | 17.78 [0.02, 0.05] | 1.54 ^ns^ | 0.97 [-0.01, 0.02] |
| Monthly Exercise | 6.15 | 7.70 *** | 313.55 [0.07, 0.09] | 8.62 *** | 708.15 [0.1, 0.12] | 7.81 *** | 81.91 [0.02, 0.03] |
| Weekly Exercise | 27.24 | 37.39 *** | 3945.08 [0.14, 0.15] | 49.59 *** | 17690.03 [0.3, 0.32] | 39.59 *** | 1327.77 [0.05, 0.05] |
| Daily Exercise | 3.17 | 3.65 *** | 54.02 [0.03, 0.06] | 4.27 *** | 259.32 [0.08, 0.11] | 3.76 *** | 18.92 [0.01, 0.02] |
| **Weight status** |  |  |  |  |  |  |  |
| Healthy Weight | 31.32 | 35.70 *** | 652.09 [0.05, 0.06] | 44.70 *** | 5453.49 [0.15, 0.16] | 36.27 *** | 193.64 [0.02, 0.02] |
| Underweight | 1.68 | 1.76 ^ns^ | 2.98 [0, 0.03] | 1.96 *** | 29.31 [0.03, 0.06] | 2.02 *** | 11.79 [0.01, 0.03] |
| Overweight | 33.76 | 32.24 *** | 73.25 [-0.02, -0.01] | 32.15 *** | 71.49 [-0.02, -0.01] | 30.60 *** | 74.99 [-0.01, -0.01] |
| Obese | 33.25 | 30.29 *** | 278.68 [-0.04, -0.03] | 21.19 *** | 3934.59 [-0.13, -0.12] | 31.11 *** | 34.5 [-0.01, 0] |
| **Smoking Status** |  |  |  |  |  |  |  |
| Non-Smoker | 66.00 | 65.79 ^ns^ | 1.43 [-0.01, 0] | 72.08 *** | 1004.59 [0.06, 0.07] | 63.63 *** | 42.33 [-0.01, -0.01] |
| Former Smoker | 22.47 | 24.30 *** | 140.17 [0.02, 0.03] | 21.31 *** | 47.12 [-0.02, -0.01] | 25.65 *** | 98.66 [0.01, 0.02] |
| Current, Some Days | 2.69 | 3.10 *** | 48.47 [0.03, 0.06] | 2.67 ^ns^ | 0.1 [-0.01, 0.01] | 3.30 *** | 23.97 [0.01, 0.03] |
| Current, Daily | 8.84 | 6.81 *** | 352.26 [-0.07, -0.06] | 3.95 *** | 1680.59 [-0.14, -0.13] | 7.42 *** | 41.49 [-0.02, -0.01] |
| **Alcohol Status** |  |  |  |  |  |  |  |
| Lifetime Abstainer | 13.38 | 8.73 *** | 1256.14 [-0.11, -0.1] | 8.50 *** | 1203.46 [-0.1, -0.09] | 7.15 *** | 550.17 [-0.04, -0.04] |
| Former Drinker | 17.22 | 16.99 ^ns^ | 2.58 [-0.01, 0] | 9.96 *** | 2156.67 [-0.12, -0.11] | 15.85 *** | 22.3 [-0.01, 0] |
| Current Drinker | 69.39 | 74.27 *** | 788.03 [0.06, 0.07] | 81.54 *** | 4142.06 [0.13, 0.14] | 77.00 *** | 455.94 [0.02, 0.03] |

Within each sociodemographic and health characteristic subgroup, Chi-square tests of independence (expressed as Chi-square value with lower and upper confidence intervals) compared the percentage of each of the three contemplative practice subgroup populations (% Meditation users, % Yoga users, and % Guided imagery/progressive relaxation users) to non-users in that subgroup. While statistical comparisons were against non-user groups, subpopulation estimates (%) and differences between US population and user population estimates (Δ %) are provided as the basis of comparison for ease of interpretation. All estimates are weighted and age-adjusted to standard 2000 US population^24^. Significance was set at false discovery rate (FDR) adjusted *p*<0.05 using the Benjamini-Hochberg method. Significance ***: p < 0.001; **: p < 0.01; *: p < 0.05; ^ns^: not significant at p < 0.05. Source: NHIS Data 2002-2022.
